# Unexpected rearrangements and a novel synthesis of 1,1-dichloro-1-alkenones from 1,1,1-trifluoroalkanones with aluminium trichloride

**DOI:** 10.3762/bjoc.17.36

**Published:** 2021-02-10

**Authors:** Beatrice Lansbergen, Catherine S Meister, Michael C McLeod

**Affiliations:** 1 Bayer AG, Research & Development Crop Science, Industriepark Höchst, 65926 Frankfurt, Germany

**Keywords:** aluminium trichloride, dichloroalkenes, Friedel–Crafts alkylation, rearrangement, trifluoroalkanes

## Abstract

A novel reactivity of 1,1,1-trifluoroalkanones is reported, where the reaction with AlCl_3_ results in the formation of 1,1-dichloro-1-alkenones. The reaction scope was found to be broad, with various chain lengths and aryl substituents tolerated. For substrates containing an electron-rich aromatic ring, further reactions take place, resulting in bicyclic and/or rearrangement products.

## Introduction

**Figure 1:**
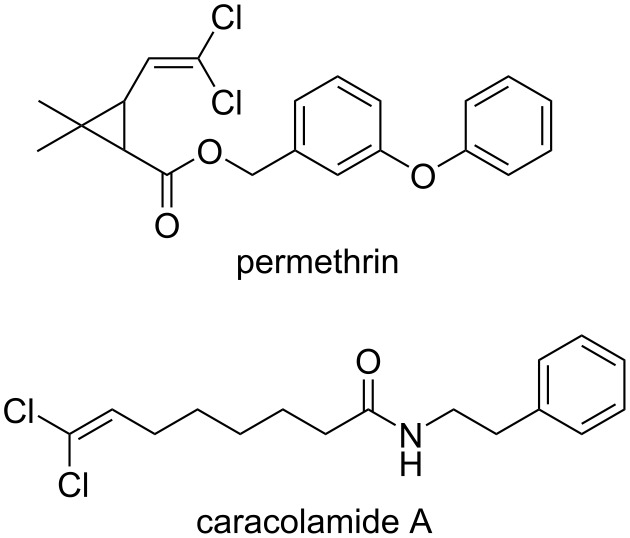
Examples of biologically active 1,1-dichloro-1-alkenes.

1,1-Dichloro-1-alkenes are valuable synthetic intermediates and have been employed in Pd-mediated cross couplings of one or both chlorine atoms [1–7], carbonylation reactions [8], and C–H bond alkynylations of heteroarenes [9]. The 1,1-dichloro-1-alkenyl moiety is found in a number of pyrethroid insecticides including permethrin and marine natural products such as caracolamide A [10] (Figure 1).

**Figure 2:**
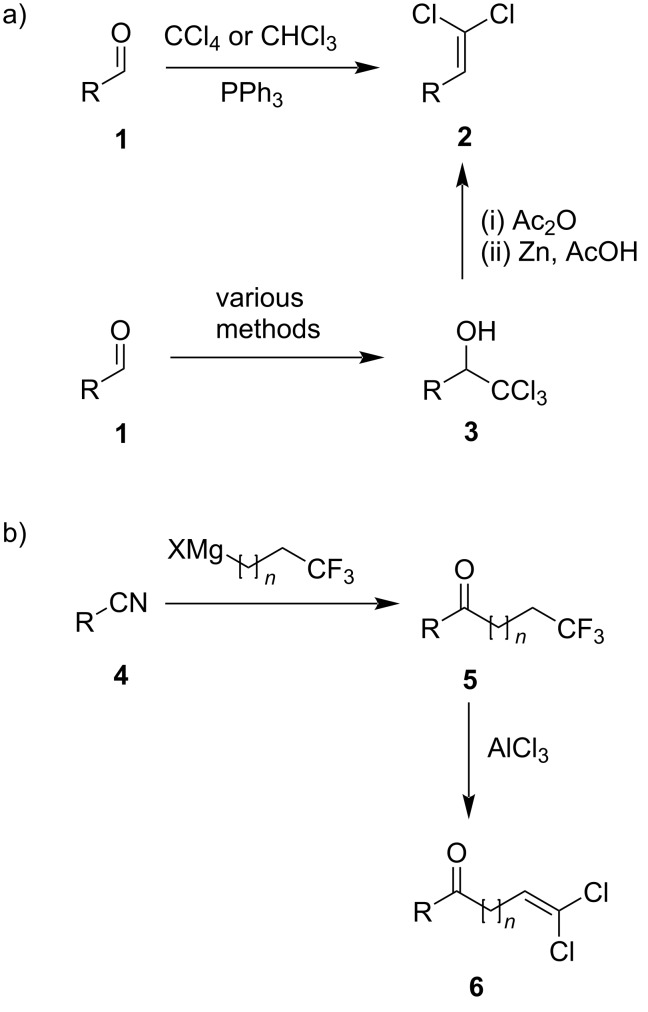
a) Common methods for the preparation of 1,1-dichloro-1-alkenes from aldehydes. b) This work: a two-step synthesis of 1,1-dichloro-1-alkenones from nitriles via 1,1,1-trifluoroalkanones.

1,1-Dichloro-1-alkenes **2** are commonly prepared from the corresponding aldehydes **1** in one step with PPh_3_ and CHCl_3_ [11], CCl_4_ [12], or with the phosphonate reagent LiCCl_2_-P(O)(OEt)_2_ [13–14] (Figure 2a). Alternatively, the aldehydes are converted to trichloromethyl carbinols **3** using various methodologies [15–17], followed by acetylation and elimination to provide the desired dichloroalkenes [18–20]. The preparation of 1,1-dichloro-1-alkenes from hydrazones [21] and from 2,2,2-trichloroethyl carbonates [22] has also been reported. Internal difluoroalkanes have been used to generate chloroalkene products using AlEt_2_Cl [23–24]. In this article we describe the AlCl_3_-mediated formation of 1,1-dichloro-1-alkenones **6** from 1,1,1-trifluoroalkanones **5**, which in turn are accessed by the Grignard addition of 1,1,1-trifluoroalkylmagnesium halides to nitriles (Figure 2b). It is worth noting that the 1,1,1-trifluoroalkyl halides (*n* = 1, 2, 3) are commercially available, whereas the corresponding 1,1-dichloroalkenyl halides (or 1,1,1-trichloroalkyl halides) are not. This methodology therefore represents a potentially useful access to the 1,1-dichloro-1-alkenone moiety.

## Findings

**Scheme 1:**
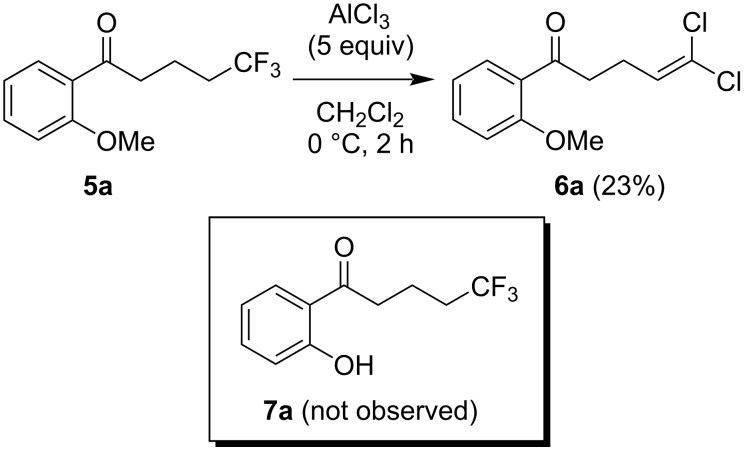
The initial attempt for the conversion of 1,1,1-trifluoroalkanone **5a** to 1,1-dichloroalkenone **6a**.

Under initial conditions, AlCl_3_ was shown to invoke the conversion of 1,1,1-trifluoroalkanone **5a** [25] to 1,1-dichloro-1-alkenone **6a** in 23% yield, without cleavage of the methyl ether [26] (Scheme 1). A similar AlCl_3_-promoted conversion of vinylic trifluoromethyl groups to 1,1-dichloroalkenes has previously been reported [27–28]. Additionally, 1,1-dichloroalkenes have also been prepared by the elimination of HCl from trichloroalkanes [29]. To the best of our knowledge, the direct conversion of an aliphatic CF_3_ group to a dichlorovinyl moiety has not been previously reported.

Encouraged by these initial findings, we performed a screen of reaction conditions using the 3-methoxy derivative **5b** (Table 1). During this optimisation process, two things became quickly apparent: (1) the dichloroalkenones **6** are generally volatile and should be handled carefully (water bath <30 °C) and (2) under certain conditions, the trichloroalkanone **8b** could also be isolated from the reactions. While the conversion of an aromatic trifluoromethyl to a trichloromethyl group with AlCl_3_ has been previously reported [30–32], investigations into the analogous transformation of aliphatic CF_3_ groups has been limited to adamantly-trifluoromethyl moieties [33–34].

**Table 1:** Optimisation of the reaction conditions for the conversion of 1,1,1-trifluoroalkanone **5b** to 1,1-dichloroalkenone **6b**.

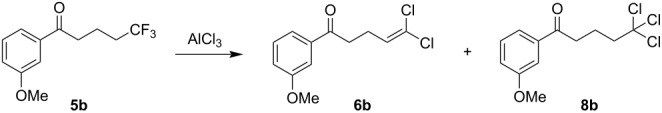					

entry	equiv AlCl_3_	solvent	temp (°C)	time (h)	ratio^a ^**5b**:**6b**:**8b**

1	5	CH_2_Cl_2_	0	1.5	0.07:1:0.15
2	5	CH_2_Cl_2_	0	1	0.45:1:0.35
3	3	CH_2_Cl_2_	0–rt	18	9.7:1:0.7
4	1	CH_2_Cl_2_	0–rt	18	n.r.^b^
5	5	PhMe	0–rt	18	complex mixture
6	5	THF	0–rt	18	n.r.^b^
7	5	DMF	0–rt	18	n.r.^b^
8	5	MeCN	0–rt	18	n.r.^b^
9	5	DCE	0	2	0.29:1:0.19
10	5	CH_2_Cl_2_	rt	2	0.05:1:0.05 (78%)^c^
11	5	CH_2_Cl_2_	40	1	complex mixture

^a^Determined by ^1^H NMR analysis; ^b^n.r. = no reaction; ^c^isolated yield of **6b**.

An NMR analysis of the crude product mixture obtained using our original reaction conditions (5 equiv AlCl_3_, CH_2_Cl_2_, 0 °C) showed that, while dichloroalkenone **6b** was the major product, a small amount of starting material **5b** was still present along with significant quantities of the trichloroalkanone **8b** (Table 1, entry 1). With a shorter reaction time of 1 h, greater quantities of the trifluoroalkanone starting material **5b** and trichloroalkanone **8b** were observed (Table 1, entry 2), suggesting that compound **8b** is an intermediate structure in the formation of dichloroalkenone **6b**. Attempts to reduce the number of equivalents of AlCl_3_ in the reaction were unsuccessful: only unreacted starting material was observed when 1 equivalent was employed, and 3 equivalents only gave low conversion (Table 1, entries 3 and 4). Replacing dichloromethane with 1,2-dichloroethane as solvent had a negligible effect on the reaction, however, all other solvents that were investigated failed to produce any desired dichloroalkenone product, with only unreacted **5b** being observed in most cases (Table 1, entries 5–9). By increasing the temperature to 40 °C, a full conversion of the starting material was observed, along with a decreased amount of the trichloroalkanone **8b**, but at the expense of the formation of a number of other unidentified byproducts (Table 1, entry 11). At room temperature, the right balance of starting material conversion and minimisation of byproduct formation was reached, so that dichloroalkenone **6b** could be isolated in 78% yield (Table 1, entry 10).

**Scheme 2:**
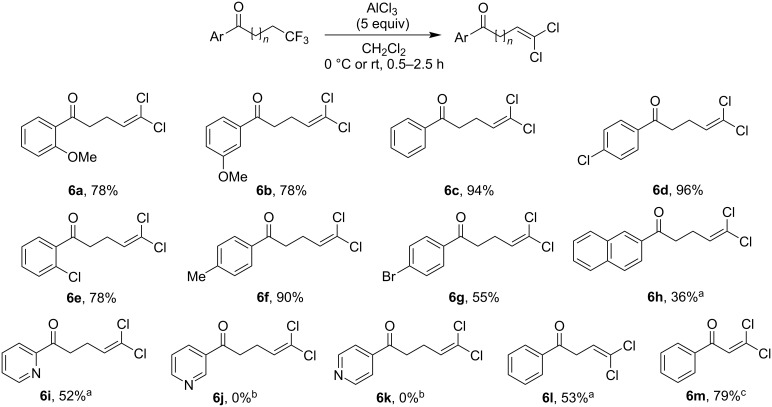
Substrate scope for the reaction of 1,1,1 trifluoroalkanones with AlCl_3_. ^a^Purification (normal or reversed-phase chromatography) required. ^b^Complex mixture. ^c^5 Days.

With an optimised set of conditions established for this reaction, we turned our attention to investigating the substrate scope (Scheme 2). The required trifluoroalkanone starting materials were prepared in one step from the corresponding benzonitrile by reaction with the required trifluoroalkylmagnesium iodide (see Supporting Information File 1).

The substrate scope of the reaction proved quite broad; all phenyl-containing substrates **5a**–**g** could be transformed into the desired products **6a**–**g**. Using these optimised conditions, the isolated yield of our original dichloroalkene **6a** (Scheme 1) could be increased from 23% to 78%. Naphthalene **6h** and 2-pyridine **6i** were also successfully obtained, however, the reactions with the 3- and 4-pyridines **5j** and **5k** resulted in complex mixtures from which no desired product, nor starting material, could be observed. The scope of this chemistry also extends to substrates with a shorter trifluoroalkanone chain, allowing the preparation of 1,1-dichlorobutenone **6l** and 1,1-dichloropropenone **6m**, although the latter compound required a considerably longer reaction time (5 days). For some of the above reactions, the crude product obtained after work-up was sufficiently clean (>90% purity as determined by ^1^H NMR) that no further purification was necessary. The other products needed to be purified by either normal and/or reversed-phase chromatography, resulting in significantly lower yields, in part due to the volatile nature of the products.

**Scheme 3:**
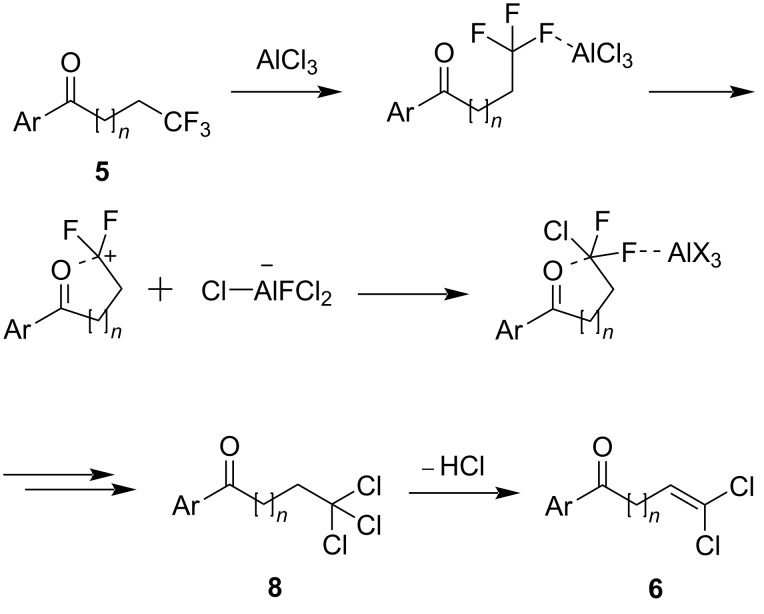
Proposed mechanism for the formation of dichloroalkene **6** from trifluoroalkanone **5**.

The longer reaction time of trifluoropentanone derivative **5m** compared to the longer chain substrates (e.g., **5c** and **5l**) could be explained mechanistically by the formation of a terminal carbocation upon the extraction of a fluorine atom by the aluminium species (Scheme 3). This carbocation could be stabilised to varying degrees by the ketone moiety, depending on the alkyl chain length. For longer chains where *n* = 1 or 2, this stabilisation would occur via a favoured 5- or 6-ring intermediate, respectively, while for shorter chains (*n* = 0), an unfavoured 4-membered ring would be required.

**Scheme 4:**
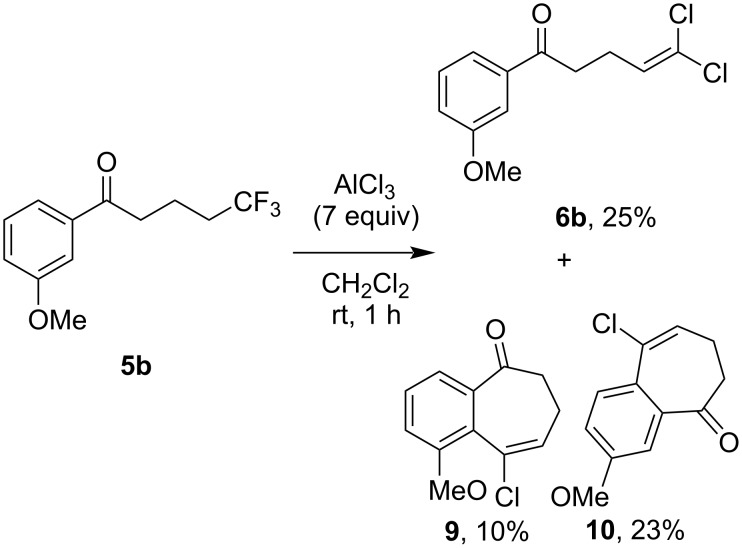
Formation of bicyclic ketones **9** and **10** from 1,1,1-trifluoroalkanone **5b** using 7 equivalents of AlCl_3_.

When the 3-methoxy derivative **5b** was treated with 7 (instead of 5) equivalents of AlCl_3_, a further two products were obtained, whose structures were elucidated as the 8,9-dihydrobenzo[7]annulen-9-ones **9** and **10** (Scheme 4). Presumably, these bicyclic compounds arise via an intramolecular Friedel–Crafts alkylation of **6b**, promoted by the *ortho*/*para*-directing nature of the methoxy substituent, and a subsequent elimination of HCl. No such transformation was observed with the other trifluoroalkanone substrates, including the 2- and 4-methoxy substrates **5a** and **5f**, presumably as only the *meta*-methoxy group is able to sufficiently activate the two positions adjacent to the ketone.

**Scheme 5:**
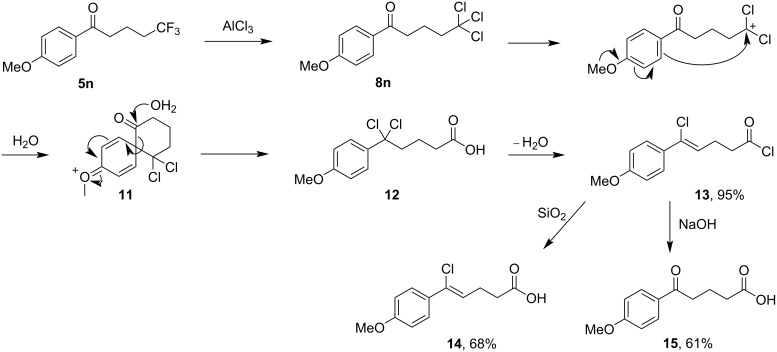
Conversion of 1,1,1-trifluoroalkanone **5n** to acid chloride **13** with AlCl_3_, and possible mechanism.

When the 4-methoxy derivative **5n** was treated with AlCl_3_ using our standard conditions, none of the expected 1,1-dichloroalkenone **6n** was obtained. Instead, the acid chloride **13** was isolated in an excellent yield (Scheme 5). The structure of this product was elucidated using 2D-NMR experiments, with an HMBC cross-peak between the aryl ring and the alkene moiety proving most illuminating (see Supporting Information File 1). The geometry of the double bond was determined from a NOE interaction between the alkene proton and the ring hydrogen atoms. This molecule could not however be detected by mass spectrometry and so it was converted to carboxylic acids **14** and **15** by the treatment with silica gel and NaOH, respectively, in order to confirm its identity. A possible mechanism for the formation of **13** from **5n** could involve the formation of a terminal carbocation from trichloroalkanone **8n**, followed by a favoured 6-*endo*-*trig* cyclisation driven by the 4-methoxy group to form the 6,6-spirocycle **11**. The addition of water and subsequent ring-opening would then form the acid **12**, which upon elimination of water would then provide the observed acid chloride **13**.

We wished to also determine whether the described reactivity would be observed with substrates not containing a ketone linker. Replacing the ketone moiety with an oxygen or sulphur atom proved unsuccessful, with only complex mixtures being obtained the upon treatment with AlCl_3_ (see Supporting Information File 1). This observation suggests that the role of the ketone moiety is not only to stabilise the putative carbocation as discussed earlier, but also to deactivate the aromatic ring to Friedel–Craft-type alkylations.

**Scheme 6:**
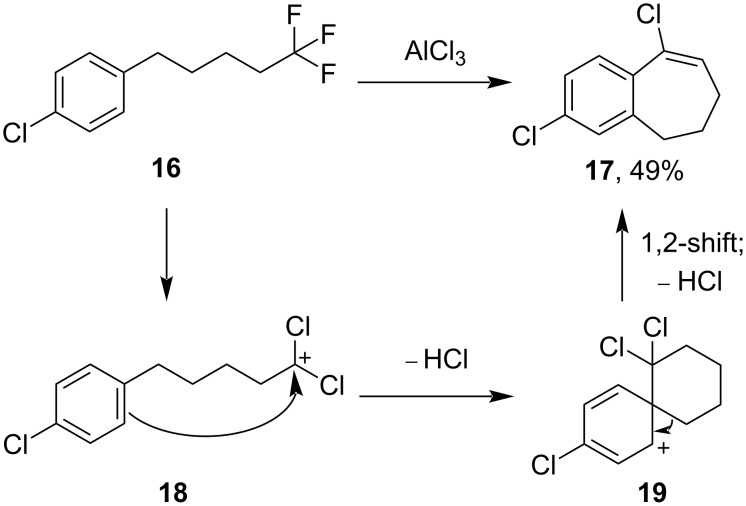
Conversion of 1,1,1-trifluoroalkane **16** to bicycle **17** upon treatment with AlCl_3_, and possible mechanism.

However, when the trifluoropentylbenzene **16** was prepared and subjected to the standard AlCl_3_ conditions (Scheme 6), a single product was isolated from the reaction that, after careful elucidation (see Supporting Information File 1), was identified as the bicycle **17**. This compound could potentially arise via an initial conversion to the dichloroalkyl cation **18**, followed by a ring closure and elimination of HCl to form the 6,6-spirocycle **19**. A 1,2-rearrangement would then produce the observed product **17**. Presumably the analogous dichloroalkenone **6d** does not undergo this cyclisation due to the deactivation of the ring by the ketone towards nucleophilic attack.

## Conclusion

In conclusion, we have shown that 1,1,1-trifluoroalkanones can be converted into 1,1-dichloro-1-alkenes when treated with AlCl_3_, allowing for an alternate and rapid route to this sought-after moiety. With 3- and 4-methoxy-substituted substrates, the initially formed dichloroalkene products are sufficiently electron rich to allow further reactions, giving rise to [6,7]bicyclic ketones **9**, and **10** and the linear acid chloride **13**, respectively. We have also found that when the ketone is not present, the trifluoroalkyl substrate is converted into bicyclic vinyl chloride **17** when treated with AlCl_3_. We hope that this short communication will inspire other researchers to utilise this functional group conversion in future syntheses of 1,1-dichloro-1-alkenes and to further investigate the unexpected reactivity of these compounds.

## Supporting Information

File 1 Detailed experimental procedures and NMR spectra for all compounds referenced.
