# Supplementary material for: Unexpected rearrangements and a novel synthesis of 1,1-dichloro-1-alkenones from 1,1,1-trifluoroalkanones with aluminium trichloride
Source: Beilstein J Org Chem. 2021 Feb 10;17:404–9. doi: 10.3762/bjoc.17.36 (PMC7884876; doi:10.3762/bjoc.17.36)
Supplement: File 1 — Detailed experimental procedures and NMR spectra for all compounds referenced. [file Beilstein_J_Org_Chem-17-404-s001.pdf]

BEILSTEIN JOURNAL OF ORGANIC CHEMISTRY

Supporting Information

for

Unexpected rearrangements and a novel synthesis of 1,1-dichloro-1-alkenones from 1,1,1-trifluoroalkanones with aluminium trichloride

Beatrice Lansbergen, Catherine S. Meister and Michael C. McLeod

Beilstein J. Org. Chem. **2021,** *17,* 404–409. doi:10.3762/bjoc.17.36

Detailed experimental procedures and NMR spectra for all compounds referenced

License and Terms: This is a supporting information file under the terms of the Creative Commons Attribution License (https://creativecommons.org/licenses/by/4.0). Please note that the reuse, redistribution and reproduction in particular requires that the author(s) and source are credited and that individual graphics may be subject to special legal provisions.
The license is subject to the *Beilstein Journal of Organic Chemistry* terms and conditions: (https://www.beilstein-journals.org/bjoc/terms)

Table of contents

1. Reactions of (thio)ethers with $AlCl_3$ S2
2. General Information S2
3. Experimental procedures and spectral data S3
4. ^{1}H- and ^{13}C-NMR spectra, 2D NMR spectra S16

1. Reactions of (thio)ethers with $AlCl_3$

Upon treatment of (thio)ethers **S2**, **S3** and **S4** with $AlCl_3$ under the standard conditions (see below), complex reaction mixtures were obtained, from which no clean products could be isolated (Scheme S1).

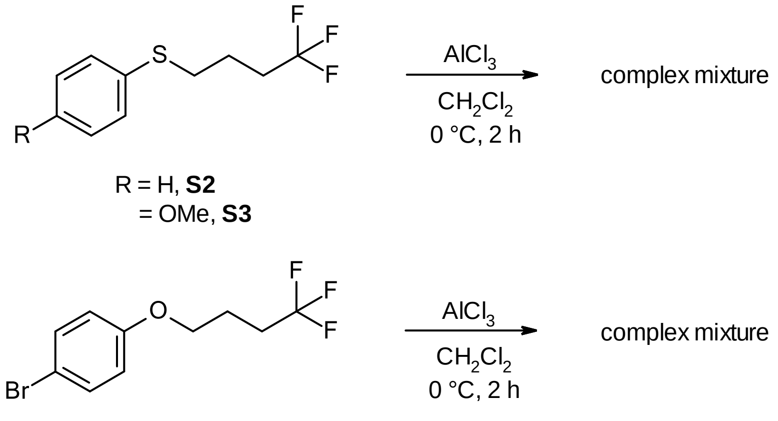

Scheme S1: Reaction of (thio)ethers with $AlCl_3$.

2. General information

All reagents bought from commercial sources were used as received without further purification. Reactions carried out at 0 °C were conducted using a water/ice bath. Anhydrous solvents were purchased from commercial suppliers. $MgSO_4$ was used as the drying reagent to dry organic phases. Normal phase column chromatography was carried out using a Biotage Isolera^{TM} using CHROMABOND flash SiOH columns (Macherey-Nagel) with columns in sizes between 15 g to 40 g. The solvent system was ethyl acetate, heptane and methanol at various gradient systems. Reversed-phase column chromatography was carried out using Biotage^{R} Ultra C_{18} SNAP 12 g column. The solvent system was water and acetonitrile at various gradient systems. If no method of purification is stated, then the product was deemed pure enough for use in subsequent steps without purification. The purity of all compounds was determined by ^{1}H NMR. All 1D and 2D NMR spectra were recorded using a Bruker AVII spectrometer with a Bruker TBI-probe. ^{1}H NMR spectra were recorded at 400 MHz or 600 MHz, ^{13}C NMR spectra were recorded at 100 or 150 MHz and ^{19}F NMR spectra were recorded at 376 MHz. $CDCl_3$ was used as the solvent. All chemical shift values are referenced to TMS and reported in ppm to 1 Hz resolution. LCMS spectra were obtained by an Agilent LC spectrometer with a 3100 Mass Detector (SQD) using electron ionization (EI) technique. High resolution mass spectrometry (HRMS) was obtained by a Waters spectrometer with Q-ToF Premiers using electron spray ionization.

3. Experimental procedures and spectral data

General procedure A for the formation of 1,1,1-trifluoroalkanones **5**

A dry 2-necked round-bottomed flask attached to a condenser was filled with magnesium (8 mmol, 2 equiv) under argon and stirred for 30 minutes. An iodine crystal was added. The mixture was warmed gently and stirred vigorously for 10 minutes. Dry diethyl ether (10 mL) was added followed by the dropwise addition of 1,1,1-trifluoro-4-iodo-butane (4 mmol, 2 equiv) at such a rate as to maintain a gentle reflux. After the addition was complete, the mixture was stirred at reflux for an additional 30 minutes.

This solution of 4,4,4-trifluorobutylmagnesium iodide (10 mL, 2 equiv) was added to a solution of the corresponding benzonitrile (4 mmol, 1 equiv) and CuBr (0.2 mmol, 5.0 mol %) in dry THF (10 mL) at 0 °C under argon. The reaction was heated to 50 °C for 2 h. Upon completion the reaction was cooled to room temperature and quenched with 2M HCl (5 mL). The aqueous layer was extracted with ethyl acetate (3 × 20 mL). The organic layers were combined, dried ($MgSO_4$), filtered, and concentrated. The resulting residue was purified by automated flash column chromatography (40 g silica, 0–100% heptane/ethyl acetate). In some cases further purification by reversed-phase column chromatography was required (12 g silica, 0–100% water/acetonitrile).

5,5,5-Trifluoro-1-(2-methoxyphenyl)pentan-1-one (5a)

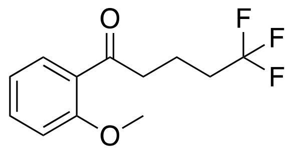

Following general procedure A, reaction of 2-methoxybenzonitrile (**4a**, 666 mg, 5.00 mmol) provided **5a** as yellow oil (290 mg, 24% yield); δ_H (400 MHz, $CDCl_3$) 7.71 (1H, dd, *J* 7.6, 1.6), 7.49-7.45 (1H, m), 7.03-6.99 (2H, m), 3.91 (3H, s), 3.08 (2H, t, *J* 6.8), 2.22-2.15 (2H, m), 2.01-1.95 (2H, m); δ_C (150 MHz, $CDCl_3$) 201.0, 158.6, 133.7, 130.3, 127.9, 127.2 (q, *J* 274.5), 120.7, 111.5, 55.4, 42.2, 33.1 (q, *J* 28.5), 16.8; m/z (ESI+) found $[M+H]^+$ 247.0956. $C_{12}H_{13}F_3O_2^+$ requires 247.0946.

5,5,5-Trifluoro-1-(3-methoxyphenyl)pentan-1-one (5b)

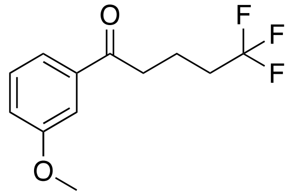

Following general procedure A, reaction of 3-methoxybenzonitrile (**4b**, 2.0 g, 15 mmol) provided **5b** as yellow oil (2.7 g, 72% yield); δ_H (400 MHz, $CDCl_3$) 7.53 (1H, d, *J* 7.6), 7.49-7.48 (1H, m), 7.38 (1H, dd, *J* 8.0, 8.0), 7.12 (1H, dd, *J* 8.0, 1.6), 3.86 (3H, s), 3.07 (2H, t, *J* 6.4), 2.25-2.18 (2H, m), 2.06-2.01 (2H, m);

δ_C (150 MHz, $CDCl_3$) 198.5, 159.9, 138.0, 129.9, 127.1 (q, *J* 274.5), 120.6, 119.7, 112.3, 55.5, 36.9, 33.0 (q, *J* 28.5), 16.5; m/z (ESI+) found $[M+H]^+$ 247.0947. $C_{12}H_{13}F_3O_2^+$ requires 247.0946.

5,5,5-Trifluoro-1-phenyl-pentan-1-one (5c)

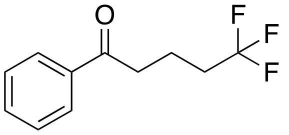

Following general procedure A, reaction of benzonitrile (**4c**, 5.0 g, 48 mmol) provided **5c** as white crystals (340 mg, 3% yield) after purification by reverse phase column chromatography; δ_H (600 MHz, $CDCl_3$) 7.96 (2H, d, *J* 7.2), 7.61-7.54 (1H, m), 7.48 (2H, dd, *J* 7.8, 7.8), 3.09 (2H, t, *J* 7.2), 2.26-2.18 (2H, m), 2.06-2.01 (2H, m); δ_C (150 MHz, $CDCl_3$) 198.6, 136.7, 133.3, 128.7, 128.1, 128.0, 127.1 (q, *J* 274.5), 36.9, 33.0 (q, *J* 28.5), 16.5; m/z (ESI+) 217.1 $[M+H]^+$. No high resolution MS could be obtained for this compound.

1-(4-Chlorophenyl)-5,5,5-trifluoro-pentan-1-one (5d)

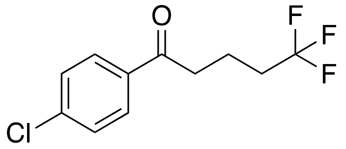

Following general procedure A, reaction of 4-chlorobenzonitrile (**4d**, 550 mg, 4.00 mmol) provided **5d** as pale yellow oil (866 mg, 86% yield); δ_H (600 MHz, $CDCl_3$) 7.90-7.88 (2H, m), 7.47-7.43 (2H, m), 3.05 (2H, t, *J* 7.2), 2.25-2.17 (2H, m), 2.08-2.00 (2H, m); δ_C (150 MHz, $CDCl_3$) 197.4, 139.8, 135.0, 129.3, 129.2, 127.1 (q, *J* 274.5), 36.9, 32.9 (q, *J* 28.5), 16.4; m/z (EI+) 250.1 $[M]^+$. No high resolution MS could be obtained for this compound.

1-(2-Chlorophenyl)-5,5,5-trifluoro-pentan-1-one (5e)

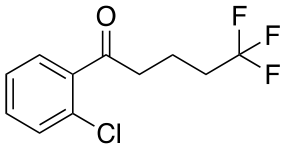

Following general procedure A, reaction of 2-chlorobenzonitrile (**4e**, 687 mg, 5.00 mmol) provided **5e** as clear oil (115 mg, 9% yield) after purification by reverse phase column chromatography; δ_H (600 MHz, $CDCl_3$) 7.46 (1H, dd, *J* 7.8, 1.8), 7.43-7.40 (2H, m), 7.35-7.32 (1H, m), 3.05 (2H, t, *J* 7.2), 2.25-2.17 (2H, m), 2.05-1.99 (2H, m); δ_C (150 MHz, $CDCl_3$) 201.9, 139.0, 131.9, 130.8, 130.6, 128.7, 127.0, 127.0 (q, *J* 274.5), 41.2, 32.8 (*J* 28.5), 16.5. m/z (ESI+) found $[M+H]^+$ 251.0455. $C_{11}H_{10}ClF_3O^+$ requires 251.0451.

5,5,5-Trifluoro-1-(*p*-tolyl)pentan-1-one (5f)

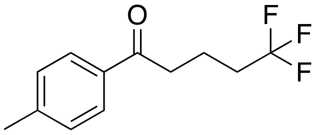

Following general procedure A, reaction of 4-methylbenzonitrile (**4f**, 486 mg, 4.00 mmol) provided **5f** as yellow oil (713 mg, 77% yield); δ_H (600 MHz, $CDCl_3$) 7.85 (2H, d, *J* 8.4), 7.26 (2H, d, *J* 8.4), 3.05 (2H, t, *J* 7.2), 2.41 (3H, s), 2.25-2.17 (2H, m), 2.05-1.98 (2H, m); δ_C (150 MHz, $CDCl_3$) 198.3, 144.1, 132.1, 129.39, 128.8, 127.2 (q, *J* 274.5), 36.8, 33.0 (q, *J* 28.5), 21.7, 16.6; m/z (ESI+) found $[M+H]^+$ 231.0994. $C_{12}H_{13}F_3O^+$ requires 231.0997.

1-(4-Bromophenyl)-5,5,5-trifluoro-pentan-1-one (5g)

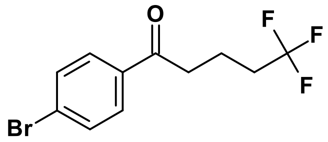

Following general procedure A, reaction of 4-bromobenzonitrile (**4g**, 4.00 g, 22.0 mmol) provided **5g** as pale yellow oil (5.20 g, 80% yield); δ_H (400 MHz, $CDCl_3$) 7.83-7.80 (2H, m), 7.63-7.30 (2H, m), 3.05 (2H, t), 2.28-2.15 (2H, m), 2.06-1.99 (2H, m); δ_C (150 MHz, $CDCl_3$) 197.6, 135.3, 132.0, 129.5, 128.5, 127.1 (q, *J* 275), 36.7, 32.9 (q, *J* 29), 16.4; m/z (ESI+) found $[M+H]^+$ 294.9957. $C_{11}H_{11}OF_3Br^+$ requires 294.9945.

5,5,5-Trifluoro-1-(2-naphthyl)pentan-1-one (5h)

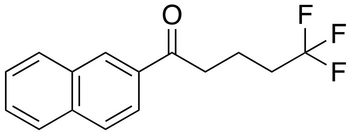

Following general procedure A, reaction of naphthalene-2-carbonitrile (**4h**, 612 mg, 4.00 mmol) provided **5h** as clear oil (340 mg, 32% yield) after purification by reverse phase column chromatography; δ_H (600 MHz, $CDCl_3$) 8.47 (1H, s), 8.03-8.02 (1H, m), 7.80 (1H, d, *J* 7.8), 7.92-7.88 (2H, m), 7.62-7.57 (2H, m), 3.23 (2H, t, *J* 7.2), 2.31-2.23 (2H, m), 2.12-2.07 (2H, m); δ_C (150 MHz, $CDCl_3$) 198.6, 135.7, 134.0, 132.5, 129.6, 129.6, 128.6, 127.8, 127.1 (q, *J* 274.5), 126.9, 123.7, 36.9, 33.0 (q, *J* 28.5), 16.6; m/z (ESI+) found $[M+H]^+$ 267.1004. $C_{15}H_{13}F_3O^+$ requires 267.0997.

5,5,5-Trifluoro-1-(2-pyridyl)pentan-1-one (5i)

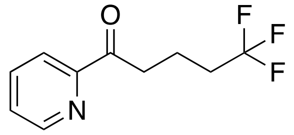

Following general procedure A and column chromatography using 0–100% heptane/ethyl acetate + 0–20% methanol gradient system, the reaction of pyridine-2-carbonitrile (**4i**, 416 mg, 4.00 mmol) provided **5i** as yellow oil (367 mg, 42% yield); δ_H (600 MHz, $CDCl_3$) 8.68 (1H, d, *J* 7.8), 8.05 (1H, d, *J* 7.8), 7.85 (1H, ddd, *J* 7.8, 7.8, 1.8), 7.49 (1H, ddd, *J* 7.8, 4.8, 1.2), 3.33 (2H, t, *J* 7.2), 2.26-2.18 (2H, m), 2.05-2.00 (2H, m); δ_C (150 MHz, $CDCl_3$) 200.5, 153.1, 149.0, 137.0, 127.3, 127.1 (q, *J* 274.5), 121.7, 36.2, 33.0 (q, *J* 28.5), 16.4; m/z (ESI+) found $[M+H]^+$ 218.0803. $C_{10}H_{10}F_3NO^+$ requires 218.0793.

5,5,5-Trifluoro-1-(3-pyridyl)pentan-1-one (5j)

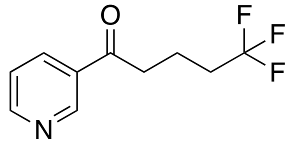

Following general procedure A and column chromatography using 0–100% heptane/ethyl acetate + 0–20% methanol gradient system, the reaction of pyridine-3-carbonitrile (**4j**, 416 mg, 4.00 mmol) provided **5j** as yellow oil (71 mg, 8% yield); δ_H (600 MHz, $CDCl_3$) 9.19 (1H, s), 8.82 (1H, d, *J* 3.6), 8.23 (1H, ddd, *J* 1.8, 2.4, 7.8), 7.44 (1H, dd, *J* 4.8, 7.8), 3.11 (2H, t, *J* 6.60), 2.28-2.20 (2H, m), 2.09-2.04 (2H, m); δ_C (150 MHz, $CDCl_3$) 197.4, 171.2, 159.9, 153.7, 149.5, 135.3, 127.0 (q, *J* 274.5), 123.8, 37.1, 32.9 (q, *J* 28.5), 16.1; m/z (ESI+) found $[M+H]^+$ 218.0803. $C_{10}H_{10}F_3NO^+$ requires 218.0793.

5,5,5-Trifluoro-1-(4-pyridyl)pentan-1-one (5k)

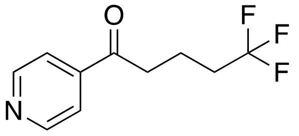

Following general procedure A and column chromatography using 0–100% heptane/ethyl acetate + 0–20% methanol gradient system, the reaction of pyridine-4-carbonitrile (**4k**, 416 mg, 4.00 mmol) provided **5k** as clear oil (64 mg, 30% yield); δ_H (600 MHz, $CDCl_3$) 8.83 (2H, d, *J* 6.6), 7.72 (2H, d, *J* 6.0), 3.10 (3H, t, *J* 7.2), 2.27-2.19 (2H, m), 2.07-2.01 (2H, m); δ_C (150 MHz, $CDCl_3$) 198.0, 151.1, 142.3, 126.9 (q, *J* 274.5), 120.8, 37.1, 32.7 (q, *J* 28.5), 16.1; m/z (ESI+) found $[M+H]^+$ 218.0799. $C_{10}H_{10}F_3NO^+$ requires 218.0793.

4,4,4-Trifluoro-1-phenyl-butan-1-one (5l)

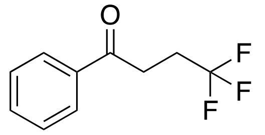

Following general procedure A, reaction of benzonitrile (**4l**, 750 mg, 7.27 mmol) with 1,1,1-trifluoro-3-iodopropane (1.71 mL, 14.5 mmol) provided **5l** as brown oil (337 mg, 23% yield); δ_H (600 MHz, $CDCl_3$) 7.97 (2H, d, *J* 7.20), 7.60 (1H, dd, *J* 7.20), 7.50-7.47 (2H, m), 3.72-3.25 (2H, m), 2.64-2.55 (2H, m); δ_C (150 MHz, $CDCl_3$) 196.4, 136.1, 133.6, 129.1, 128.6, 128.0, 127.2 (q, *J* 274.5), 31.32, 28.3 (q, *J* 28.5); m/z (ESI+) 202.1 $[M+H]^+$. No high resolution MS could be obtained for this compound.

5,5,5-Trifluoro-1-(4-methoxyphenyl)pentan-1-one (5n)

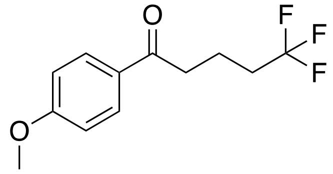

Following general procedure A, reaction of 4-methoxybenzonitrile (**4m**, 4.0 g, 30.0 mmol) provided **5m** as pale yellow oil (6.6 g, 89% yield); δ_H (600 MHz, $CDCl_3$) 7.95-7.92 (2H, m), 6.95-6.93 (2H, m), 3.87 (3H, s), 3.03 (2H, t, *J* 7.2), 2.25-2.18 (2H, m), 2.04-1.99 (2H, m); δ_C (150 MHz, $CDCl_3$) 197.2, 163.6, 130.2, 130.0, 127.2 (q, *J* 274.5), 114.0, 55.5, 36.5, 33.1 (q, *J* 28.5), 16.7; m/z (ESI+) found $[M+H]^+$ 247.0942. $C_{12}H_{13}F_3O_2^+$ requires 247.0946.

General procedure B for the preparation of 1,1-dichloro-1-alkenes

The corresponding 1,1,1-trifluoroalkanone (200 mg, 1.00 equiv) was dissolved in CH_2Cl_2 (3 mL) in a Radley tube under argon. To this solution was added $AlCl_3$ (542 mg, 5.00 equiv) at 0 °C. The solution was stirred at room temperature for 0.5–2.5 h unless stated otherwise. Upon completion of the reaction (monitored by LCMS) the reaction was quenched with 2M HCl and extracted with CH_2Cl_2 (3 × 10 mL). The combined organic layers were dried ($MgSO_4$), filtered, and concentrated. If required, the resulting residue was purified by either flash column chromatography (15 g silica, 0–100% heptane/ethyl acetate) or reversed-phase column chromatography (12 g silica, 0–100% water/acetonitrile).

5,5-Dichloro-1-(2-methoxyphenyl)pent-4-en-1-one (6a)

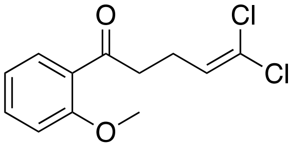

Following general procedure B, reaction of **5a** (200 mg, 0.81 mmol) (1 h) provided **6a** as pale yellow oil (164 mg, 78% yield) with no purification required; δ_H (600 MHz, $CDCl_3$); 7.72 (1H, dd, *J* 7.8, 1.2), 7.49-7.46 (1H, m), 7.01 (1H, dd, *J* 7.8, 7.8), 6.97 (1H, d, *J* 8.4), 5.97 (1H, t, *J* 7.2), 3.92 (3H, s), 3.10 (2H, t, *J* 7.2), 2.56 (2H, dt, *J* 7.2, 7.2); δ_C (150 MHz, $CDCl_3$) 200.6, 158.7, 133.8, 130.4, 129.1, 127.7, 120.7, 120.5, 111.6, 55.5, 41.9, 24.5; m/z (EI+) 259.3 $[M+H]^+$. No high resolution MS could be obtained for this compound.

5,5-Dichloro-1-(3-methoxyphenyl)pent-4-en-1-one (6b)

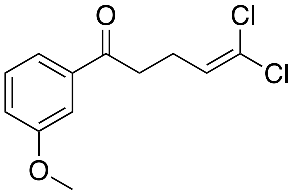

Following general procedure B, reaction of **5b** (244 mg, 0.99 mmol) (1 h) provided **6b** as dark green oil (201 mg, 78% yield) with no purification required; δ_H (600 MHz, $CDCl_3$) 7.53 (1H, d, *J* 7.2), 7.49-7.48 (1H, m), 7.38 (1H, dd, *J* 7.8, 7.8) 7.12 (1H, dd, *J* 7.8, 2.4), 5.99 (1H, t, *J* 7.2), 3.86 (3H, s), 3.10 (2H, t, *J* 7.2), 2.60 (2H, dt, *J* 7.2, 7.2); δ_C (150 MHz, $CDCl_3$) 198.2, 159.9, 137.9, 129.7, 128.5, 121.2, 120.6, 119.8, 112.3, 55.5, 36.8, 24.1; m/z (ESI+) found $[M+H]^+$ 259.0302. $C_{12}H_{12}Cl_2O_2^+$ requires 259.0293.

5,5,5-Trichloro-1-(3-methoxyphenyl)pentan-1-one (8b)

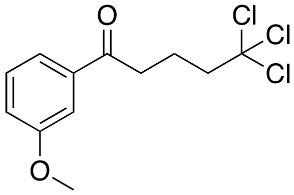

Following General Procedure B, reaction **5b** (200 mg, 0.81 mmol) (0 °C, 1.5 h) provided **8b** as clear oil (56 mg, 23% yield) after purification by reverse phase chromatography; δ_H (600 MHz, $CDCl_3$) 7.54 (1H, d, *J* 7.8), 7.50 (1H, s), 7.39 (1H, dd, *J* 7.8, 7.8), 7.13 (1H, dd, *J* 7.8, 1.8), 3.87 (3H, s), 3.11 (2H, t, *J* 7.2), 2.79-2.82 (2H, m), 2.24-2.26 (2H, m); δ_C (150 MHz, $CDCl_3$) 198.5, 159.9, 138.0, 129.7, 120.5, 119.7, 116.3, 112.3, 99.6, 55.5, 54.3, 36.8, 21.0; m/z (ESI+) found $[M+H]^+$ 295.0068. $C_{12}H_{13}Cl_3O_2^+$ requires 295.0059.

5,5-Dichloro-1-phenyl-pent-4-en-1-one (6c)

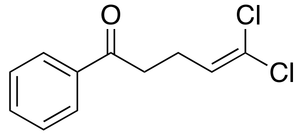

Following general procedure B, reaction of **5c** (170 mg, 0.78 mmol) (40 °C, 1 h) provided **6c** as yellow oil (170 mg, 94% yield) with no purification required; δ_H (600 MHz, $CDCl_3$) 7.96 (2H, d, *J* 7.8), 7.59-7.57 (1H, m), 7.49-7.46 (2H, m), 6.00 (1H, t, *J* 7.2), 3.12 (2H, t, *J* 7.2), 2.61 (2H, dt, *J* 7.2, 7.2); δ_C (150 MHz, $CDCl_3$) 198.7, 136.7, 133.3, 128.7, 128.5, 128.0, 121.1, 36.7, 24.0; m/z (EI+) 229.3 $[M+H]^+$. No high resolution MS could be obtained for this compound.

5,5-Dichloro-1-(4-chlorophenyl)pent-4-en-1-one (6d)

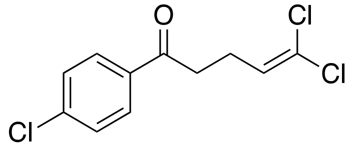

Following general procedure B, reaction of **5d** (200 mg, 0.79 mmol) (1 h) provided **6d** as pale yellow oil (202 mg, 96% yield) with no purification required; δ_H (600 MHz, $CDCl_3$) 7.90-7.89 (2H, m), 7.45 (2H, m), 5.99 (1H, t, *J* 7.2), 3.08 (2H, t, *J* 7.2), 2.60 (2H, dt, *J* 7.2, 7.2); δ_C (150 MHz, $CDCl_3$) 197.4, 139.8, 136.5, 129.4, 129.0, 128.3, 121.4, 36.7, 24.0; m/z (ESI+) found $[M+H]^+$ 262.9814. $C_{11}H_9Cl_3O^+$ requires 262.9797.

5,5-Dichloro-1-(2-chlorophenyl)pent-4-en-1-one (6e)

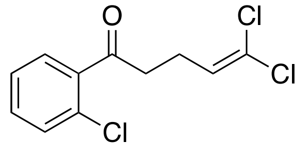

Following general procedure B, reaction of **5e** (115 mg, 0.45 mmol) (40 min) provided **6e** as clear oil (92 mg, 78% yield) with no purification required; δ_H (600 MHz, $CDCl_3$) 7.47 (1H, dd, *J* 7.8, 1.2), 7.43-7.38 (2H, m), 7.35-7.32 (1H, m), 5.96 (1H, t, *J* 7.2), 3.09 (2H, t, *J* 7.2), 2.59 (2H, dt, *J* 7.2, 7.2); δ_C (150 MHz, $CDCl_3$) 201.6, 138.9, 132.0, 131.0, 130.6, 129.0, 128.0, 127.0, 121.4, 40.9, 24.6. m/z (ESI+) found $[M+H]^+$ 262.9799. $C_{11}H_9Cl_3O^+$ requires 262.9797.

5,5-Dichloro-1-(*p*-tolyl)pent-4-en-1-one (6f)

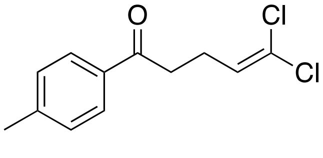

Following general procedure B, reaction of **5f** (200 mg, 0.86 mmol) (1 h) provided **6f** as pale yellow oil (190 mg, 90% yield) with no purification required; δ_H (600 MHz, $CDCl_3$) 7.85 (2H, d, *J* 8.4), 7.27 (2H, d, *J* 8.4), 6.00 (1H, t, *J* 7.2), 3.09 (2H, t, *J* 7.2), 2.60 (2H, dt, *J* 7.2, 7.2), 2.42 (3H, s); δ_C (150 MHz, $CDCl_3$) 198.3, 144.1, 134.1, 129.4, 128.6, 128.1, 121.0, 36.6, 24.1, 21.7; m/z (ESI+) found $[M+H]^+$ 243.0354. $C_{12}H_{12}Cl_2O^+$ requires 243.0343.

5,5-Dichloro-1-(4-bromophenyl)pent-4-en-1-one (6g)

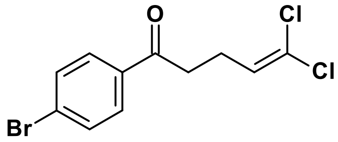

Following general procedure B, reaction of **5g** (250 mg, 0.85 mmol) (0 °C, 1 h) provided **6g** as colourless solid (145 mg, 55% yield) after purification by reverse phase column chromatography; δ_H (600 MHz, $CDCl_3$) 7.82-7.80 (2H, m), 7.62-7.60 (2H, m), 5.98 (2H, t), 3.08 (2H, t), 2.61-2.58 (2H, m); δ_C (150 MHz, $CDCl_3$) 197.3, 135.2, 132.2, 129.7, 128.5, 128.3, 121.3, 36.6, 24.0; m/z (ESI+) found $[M+H]^+$ 306.9247. $C_{11}H_{10}OCl_2Br^+$ requires 306.9292.

5,5-Dichloro-1-(2-naphthyl)pent-4-en-1-one (6h)

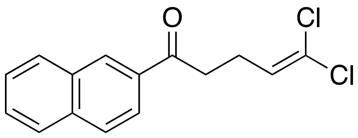

Following general procedure B, reaction of **5h** (140 mg, 0.52 mmol) (0 °C, 1 h) provided **6h** as pale yellow oil (27 mg, 36% yield) after purification of half the crude residue by reverse phase column chromatography; δ_H (600 MHz, $CDCl_3$) 8.47 (1H, s), 8.02 (1H, dd, *J* 7.8, 1.8), 7.96 (1H, d, *J* 7.8), 7.90 (1H, d, *J* 8.4), 7.88 (1H, d, *J* 8.4), 7.61 (1H, dd, *J* 8.4, 8.4), 7.56 (1H, dd, *J* 8.4, 8.4), 6.05 (1H, t, *J* 7.2), 3.25 (2H, t, 7.2), 2.66 (2H, dt, *J* 7.2, 7.2); δ_C (150 MHz, $CDCl_3$) 198.3, 135.7, 133.9, 132.5, 129.8, 129.7, 128.6, 127.8, 126.9, 123.7, 121.2, 36.9, 24.2; m/z (ESI+) found $[M+H]^+$ 279.0349. $C_{15}H_{12}Cl_2O^+$ requires 279.0343.

5,5-Dichloro-1-(2-pyridyl)pent-4-en-1-one (6i)

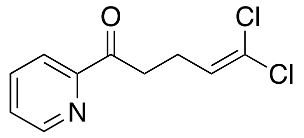

Following general procedure B, reaction of **5i** (200 mg, 0.92 mmol) (0 °C, 1 h) provided **6i** as yellow oil (110 mg, 52% yield) after purification by reverse phase column chromatography; δ_H (600 MHz, $CDCl_3$) 8.68 (1H, d, *J* 7.2), 8.04 (1H, d, *J* 7.2), 7.85 (1H, ddd, *J* 7.2, 4.8, 1.2), 7.49 (1H, ddd, *J* 7.2, 4.8, 1.2), 6.00 (1H, t, *J* 7.2), 3.37 (2H, t, *J* 7.2), 2.60 (2H, dt, *J* 7.2, 7.2); δ_C (150 MHz, $CDCl_3$) 198.4, 151.1, 147.1, 135.1, 126.9, 125.5, 119.9, 119.0/114.5, 51.6, 34.2, 22.1; m/z (ESI+) found $[M+H]^+$ 230.0133. $C_{10}H_9Cl_2NO^+$ requires 230.0139.

4,4-Dichloro-1-phenyl-but-3-en-1-one (6l)

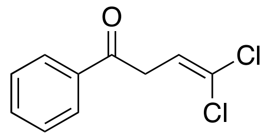

Following general procedure B, reaction of **5l** (200 mg, 0.98 mmol) (rt, 1 h) provided **6l** as yellow oil (113 mg, 53% yield) after purification by flash column chromatography; δ_H (600 MHz, $CDCl_3$) 7.97 (2H, dd, *J* 7.2, 1.2), 7.67-7.59 (1H, m), 7.51-7.46 (2H, m), 6.35 (1H, t, *J* 6.60), 3.90 (2H, d, *J* 6.60); δ_C (150 MHz, $CDCl_3$) 195.0, 136.1, 133.6, 128.8, 128.2, 122.7, 122.5, 39.2; m/z (CI+NH_3) 214.8 $[M+H]^+$, 231.8 $[M+NH_4]^+$. The spectroscopic data is consistent with those in the literature.[1]

3,3-Dichloro-1-phenyl-prop-2-ene-1-one (6m)

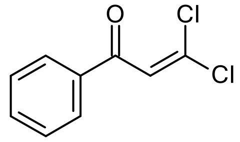

Following general procedure B, reaction of 3,3,3-trifluoro-1-phenyl-1-propanone (300 mg, 1.59 mmol) with 10.0 equiv of $AlCl_3$ (5 d) provided **6m** as brown oil (252 mg, 79% yield) with no purification required; δ_H (600 MHz, $CDCl_3$) 7.96-7.91 (2H, m), 7.65-7.59 (1H, m), 7.52-7.48 (2H, m), 7.27 (1H, s), δ_C (150 MHz, $CDCl_3$) 186.6, 136.9, 135.5, 133.7, 128.9, 128.9, 124.1; m/z 201.0 $[M+H]^+$. The spectroscopic data is consistent with those in the literature.[2]

Preparation of dihydrobenzo[7]annulenones **9** and **10**

5,5,5-Trifluoro-1-(3-methoxyphenyl)pentan-1-one (**5b**, 200 mg, 0.81 mmol, 1.00 equiv) was dissolved in CH_2Cl_2 (3 mL) in a Radley tube under argon. To this solution was added $AlCl_3$ (758 mg, 5.68 mmol, 7.00 equiv) at 0 °C. The solution was stirred at 0 °C for 4 h and warmed to room temperature overnight. The reaction was quenched with 2M HCl and extracted with CH_2Cl_2 (3 × 10 mL). The combined organic layers were dried ($MgSO_4$), filtered, and concentrated. The resulting residue was purified by C18 reversed-phase column chromatography(0–100% water/acetonitrile) to obtain products **9** and **10**.

5-Chloro-4-methoxy-7,8-dihydrobenzo[7]annulen-9-one (9)

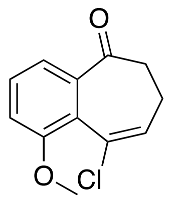

Yellow oil (18 mg, 10% yield); δ_H (600 MHz, $CDCl_3$) 7.41 (1H, dd, *J* 8.4), 7.11-7.07 (2H, m), 6.45 (1H, t, *J* 7.8), 3.93 (3H, s), 2.87 (2H, t, *J* 6.0), 2.36-2.33 (2H, m); δ_C (150 MHz, $CDCl_3$) 205.0, 156.9, 140.1, 130.7, 130.6, 129.1, 123.1, 119.4, 114.9, 56.3, 47.3, 22.6; m/z (ESI+) 223.1 [MH^+]. No high resolution MS could be obtained for this compound.

5-Chloro-2-methoxy-7,8-dihydrobenzo[7]annulen-9-one (10)

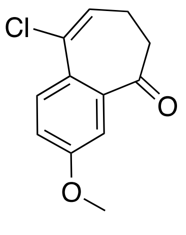

Yellow oil (41 mg, 23% yield); δ_H (600 MHz, $CDCl_3$) 7.67 (1H, d, *J* 9.0), 7.20 (1H, s), 7.09 (1H, dd, *J* 9.0, 3.0), 6.47 (1H, t, *J* 10.2), 3.87 (3H, s), 2.94-2.92 (2H, m), 2.38-2.35 (2H, m); δ_C (150 MHz, $CDCl_3$) 203.8, 159.7, 138.9, 131.9, 131.8, 130.8, 127.3, 118.8, 114.7, 56.3, 46.2, 22.1; m/z (ESI+) 223.1 [MH^+]. No high resolution MS could be obtained for this compound.

Reactions of *p*-methoxy derivative **5m** to obtain **13, 14**, and **15**

(*Z*)-5-Chloro-5-(4-methoxyphenyl)pent-4-enoyl chloride (13)

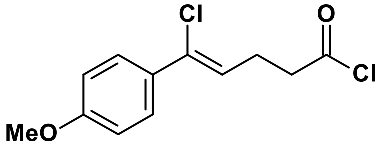

Following general procedure B, reaction of 5,5,5-trifluoro-1-(4-methoxyphenyl)pentan-1-one (**5m**, 200 mg, 0.81 mmol) (rt, 2.5 h) provided **13** as dark oil (190 mg, 95% yield) with no purification required; δ_H (600 MHz, $CDCl_3$) 7.48 (2H, d, *J* 9.0), 6.87 (2H, d, *J* 9.0), 6.01 (1H, t, *J* 7.2), 3.82 (3H, s), 3.10 (2H, t, *J* 7.2), 2.75 (2H, dt, *J* 7.2, 7.2); δ_C (150 MHz, $CDCl_3$) 173.2, 160.1, 135.3, 130.2, 127.8, 121.4, 113.7, 55.4, 45.8, 25.1. m/z (ESI+) shows only mass of corresponding acid: found 241.0642. $C_{12}H_{14}ClO_3^+$ requires 241.0631.

(*Z*)-5-Chloro-5-(4-methoxyphenyl)pent-4-enoic acid (14)

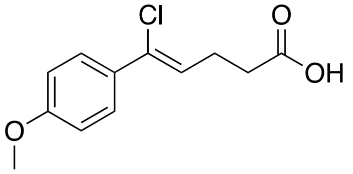

Upon purification of 50% of the above reaction by silica gel flash column chromatography the carboxylic acid derivative was formed and isolated as white solid (67 mg, 68% yield); δ_H (600 MHz, $CDCl_3$) 7.49 (2H, d, *J* 9.0), 6.86 (2H, d, *J* 9.0), 6.05 (1H, t, *J* 7.2), 3.82 (3H, s), 2.69 (2H, dt, *J* 7.2, 7.2), 2.57 (2H, t, *J* 7.2); δ_C (150 MHz, $CDCl_3$) 178.3, 159.1, 134.1, 130.6, 127.8, 123.3, 113.6, 55.4, 32.8, 24.7; m/z (ESI+) found $[M-H]^-$ 239.0471. $C_{12}H_{12}Cl_2O_2^-$ requires 239.0475.

5-(4-Methoxyphenyl)-5-oxo-pentanoic acid (15)

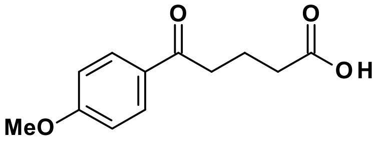

The remaining 50% of (*Z*)-5-chloro-5-(4-methoxyphenyl)pent-4-enoyl chloride (**13**) was suspended in 6M NaOH (3 mL) and stirred at rt overnight. The mixture was acidified with 2M HCl, then extracted with EtOAc (× 3). The combined organics were dried ($MgSO_4$) and concentrated. The residue was purified by silica gel chromatography to afford **15** as a colourless solid (55 mg, 61%). δ_H (400 MHz, $CDCl_3$) 7.96-7.94 (2H, m), 6.95-6.92 (2H, m), 3.87 (3H, s), 3.03 (2H, t), 2.50 (2H, t), 2.08 (2H, quintet); δ_C (100 MHz, $CDCl_3$) 198.0, 178.3, 163.5, 130.3, 129.9, 113.8, 55.5, 37.0, 33.0, 19.2. The spectroscopic data is consistent with those in the literature.[3]

Reaction of (trifluoropentyl)benzene **16** to obtain **17**

1-(4-Chlorophenyl)-5,5,5-trifluoro-pentan-1-ol (S-1)

1-(4-Chlorophenyl)-5,5,5-trifluoro-pentan-1-one (570 mg, 2.27 mmol, 1.00 equiv) was dissolved in methanol (25 mL) and to this solution was added sodium borohydride (103 mg, 2.73 mmol, 1.20 equiv) under argon. The mixture was stirred at room temperature for 3 h and completion determined by LCMS. Then, water was added to the resulting mixture. Methanol was removed in vacuo and the remaining aqueous layer was extracted with ethyl acetate (3 × 50 mL). The organic layer was washed with water (20 mL) and brine (20 mL), then dried ($MgSO_4$), filtered, and concentrated. The desired product was obtained as yellow oil without the need for purification.

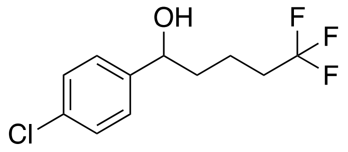

Yellow oil (523 mg, 91% yield); δ_H (600 MHz, $CDCl_3$) 7.32 (2H, d, *J* 8.4), 7.26 (2H, d, *J* 7.2), 4.66-4.64 (1H, m), 2.13-2.05 (2H, m), 1.83-1.76 (2H, m), 1.75-1.66 (2H, m); δ_C (150 MHz, $CDCl_3$) 142.7, 133.5, 128.8, 127.1 (q, *J* 274.5), 126.9, 73.6, 37.7, 33.5 (q, *J* 28.5), 18.4; m/z (ESI+) found [MH^+-H_2O] 235.0494. $C_{11}H_{12}ClF_3O^+$-H_2O requires 235.0501.

1-Chloro-4-(5,5,5-trifluoropentyl)benzene (16)

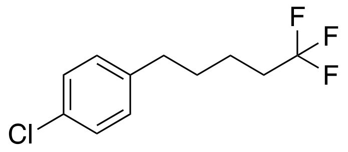

1-(4-Chlorophenyl)-5,5,5-trifluoro-pentan-1-ol (**S-1**, 500 mg, 1.98 mmol, 1.00 equiv) was dissolved in CH_2Cl_2 (30 mL) and to this solution were added trimethylsilane (4.71 mL, 29.7 mmol, 15.0 equiv) and boron trifluoride ethyl etherate (1.00 mL, 7.92 mmol, 4.00 equiv) at 0 °C under argon. The mixture was stirred at room temperature for 4 h and completion monitored by LCMS. To the mixture, water was added (15 mL) and the layers were separated. The organic layer was washed with water and dried ($MgSO_4$), filtered, and concentrated. The resulting residue was purified by flash column chromatography (40 g silica, 0-100% heptane/ethyl acetate) to obtain the desired product as colourless oil (160 mg, 34% yield); δ_H (600 MHz, $CDCl_3$) 7.24 (2H, d, *J* 8.4), 7.09 (2H, d, *J* 8.4), 2.60 (2H, t, *J* 7.8), 2.12-2.04 (2H, m), 1.70-1.63 (2H, m), 1.61-1.54 (2H, m); δ_C (150 MHz, $CDCl_3$) 140.3, 131.7, 129.9, 128.5, 127.2 (q, J 274.5), 34.7, 33.6 (q, *J* 28.5), 30.3, 14.1; m/z (EI+) 236.0 $[M+H]^+$. No high resolution MS could be obtained for this compound.

2,5-Dichloro-8,9-dihydro-7*H*-benzo[7]annulene (17)

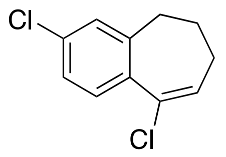

1-Chloro-4-(5,5,5-trifluoropentyl)benzene (**16**, 160 mg, 0.67 mmol, 1.00 equiv) was dissolved in CH_2Cl_2 (3 mL) in a Radley tube under argon. To this solution was added $AlCl_3$ (450 mg, 3.38 mmol, 5.00 equiv) at 0 °C. The solution was stirred at 0 °C for 60 min. Upon completion (LCMS), the reaction was quenched with 2M HCl and extracted with CH_2Cl_2 (3 × 10 mL). The combined organic layers were dried ($MgSO_4$), filtered, and concentrated. The resulting residue was purified by flash column chromatography (15 g silica, 0-100% heptane/ethyl acetate) to afford a yellow oil (70 mg, 49% yield); δ_H (600 MHz, $CDCl_3$) 7.51 (1H, d, *J* 8.4), 7.26 (1H, dd, *J* 8.4, 3.6), 7.19 (1H, d, *J* 2.4), 6.39 (1H, t, *J* 7.2), 2.64 (2H, t, *J* 6.6), 2.13-2.08 (2H, m), 1.99 (2H, dd, *J* 8.4, 7.2); δ_C (150 MHz, $CDCl_3$) 142.7, 136.0, 133.8, 129.7, 129.6, 129.5, 128.6, 126.4, 33.2, 32.2, 26.0; m/z (ESI+) found $[M+H]^+$ 213.0248. $C_{11}H_{10}Cl_2^+$ requires 213.0238.

General procedure C for the formation of ethers and thioethers

Thiophenol or phenol (1.0 equiv) were dissolved in THF or DMF. Potassium carbonate (1.5 equiv) was added to the mixture, followed by 1,1,1-trifluoro-4-iodobutane (1.5 equiv). The reaction was heated to 65 °C until completion as determined by LCMS. The reaction mixture was cooled and filtered. The filtrate was concentrated, and the resulting residue was purified by automated flash column chromatography (40 g silica, 0–100% heptane/ethyl acetate).

4,4,4-Trifluorobutylsulfanylbenzene (S-2)

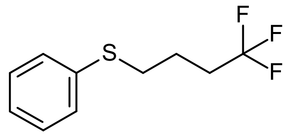

Following general procedure C, the reaction of thiophenol (1.0 g) provided **S-2** as clear oil (1.8 g, 90% yield); δ_H (400 MHz, $CDCl_3$) 7.37-7.19 (5H, m), 2.97 (2H, t, J 8.0), 2.31-2.19 (2H, m), 1.92-1.85 (2H, m), δ_C (150 MHz, $CDCl_3$) 135.3, 130.0, 129.7, 127.0 (q, J 274.5), 126.6, 32.9, 32.5 (q, J 28.5), 21.6, m/z (ESI+) found to oxidise to sulfoxide $[M+H]^+$ 237.0568. $C_{10}H_{12}F_3OS^+$ requires 237.0555.

1-Methoxy-4-(4,4,4-trifluorobutylsulfanyl)benzene (S-3)

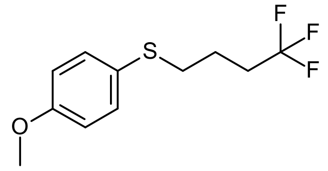

Following general procedure C, reaction of 4-methoxythiophenol (1.0 g) provided **S-3** as yellow oil (1.2 g, 68% yield); δ_H (600 MHz, $CDCl_3$) 7.40-7.35 (2H, m), 6.86-6.83 (2H, m), 3.80 (3H, s), 2.85 (2H, t, J 6.0), 2.27-2.19 (2H, m), 1.81 (2H, t, J 6.0), δ_C (150 MHz, $CDCl_3$) 134.1, 133.8, 127.0 (q, J 274.0), 114.7, 114.6, 55.4, 35.0, 32.4 (q, J 28.5), 21.6, m/z (ESI+) found to oxidise to sulfoxide $[M+H]^+$ 267.0667. $C_{11}H_{14}F_3O_2S^+$ requires 267.0661.

1-Bromo-4-(4,4,4-trifluorobutoxy)benzene (S-4)

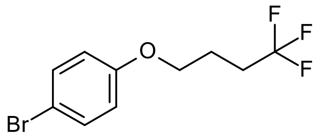

Following general procedure C, reaction of 4-bromophenol (1.0 g) provided **S-4** as clear oil (1.1 g, 68% yield); δ_H (600 MHz, $CDCl_3$) 7.38-7.36 (2H, m), 6.77-6.76 (2H, m), 3.98-3.97 (2H, m), 2.34-2.27 (2H, m), 2.05-2.04 (2H, m), δ_C (150 MHz, $CDCl_3$) 157.7, 132.6, 127.1 (q, J 274.5), 116.4, 113.2, 66.3, 30.7 (q, J 28.5), 22.1, m/z (ESI+) not found.

References:

[1] Guirado. A; Martiz. B; Andreu. R; Bautista. D; Galvez. J. *Tetrahedron* **2007**, 63, 1175-1182.
[2] Ram, R.-N; Soni, V.-K. *J. Org. Chem.* **2015**, 80, 8922-8928.
[3] Hou, R.-S.; Wang, H.-M.; Lin, Y.-C.; Chen, L.-C. *Heterocycles* **2005**, 65, 649-656.

4. ^{1}H- and ^{13}C-NMR spectra, 2D NMR spectra

5,5,5-Trifluoro-1-(2-methoxyphenyl)pentan-1-one (5a)

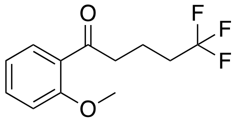

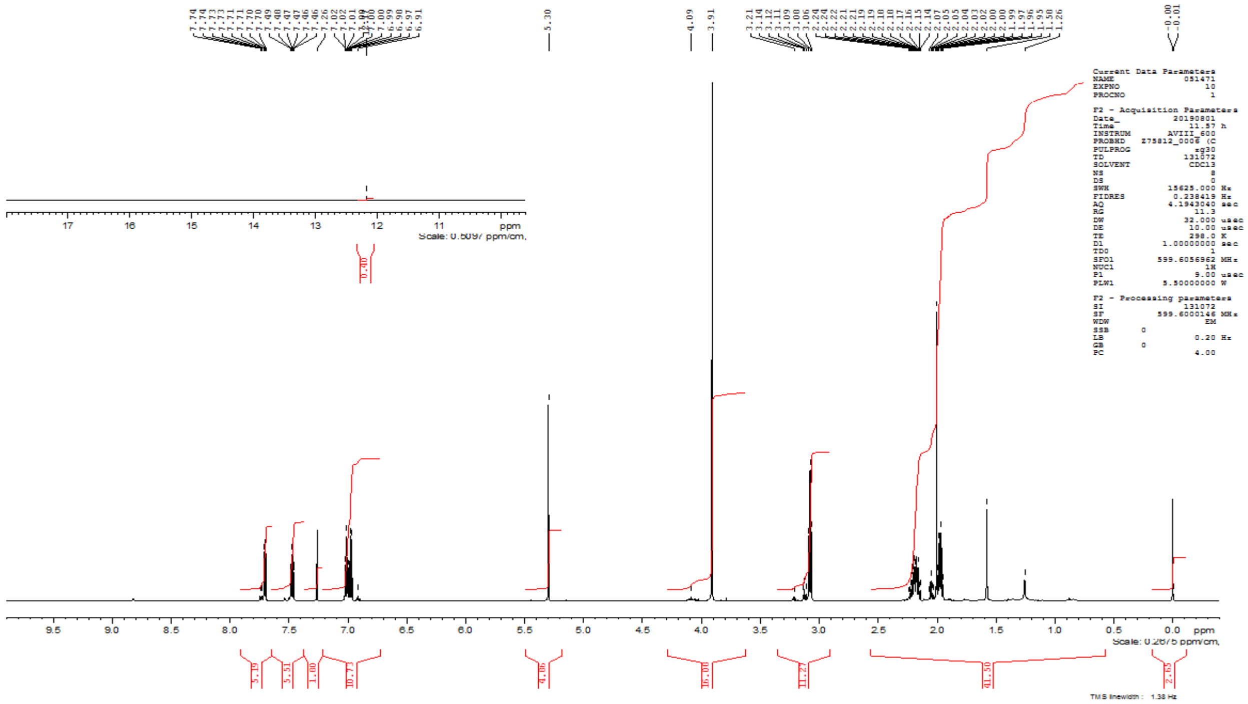

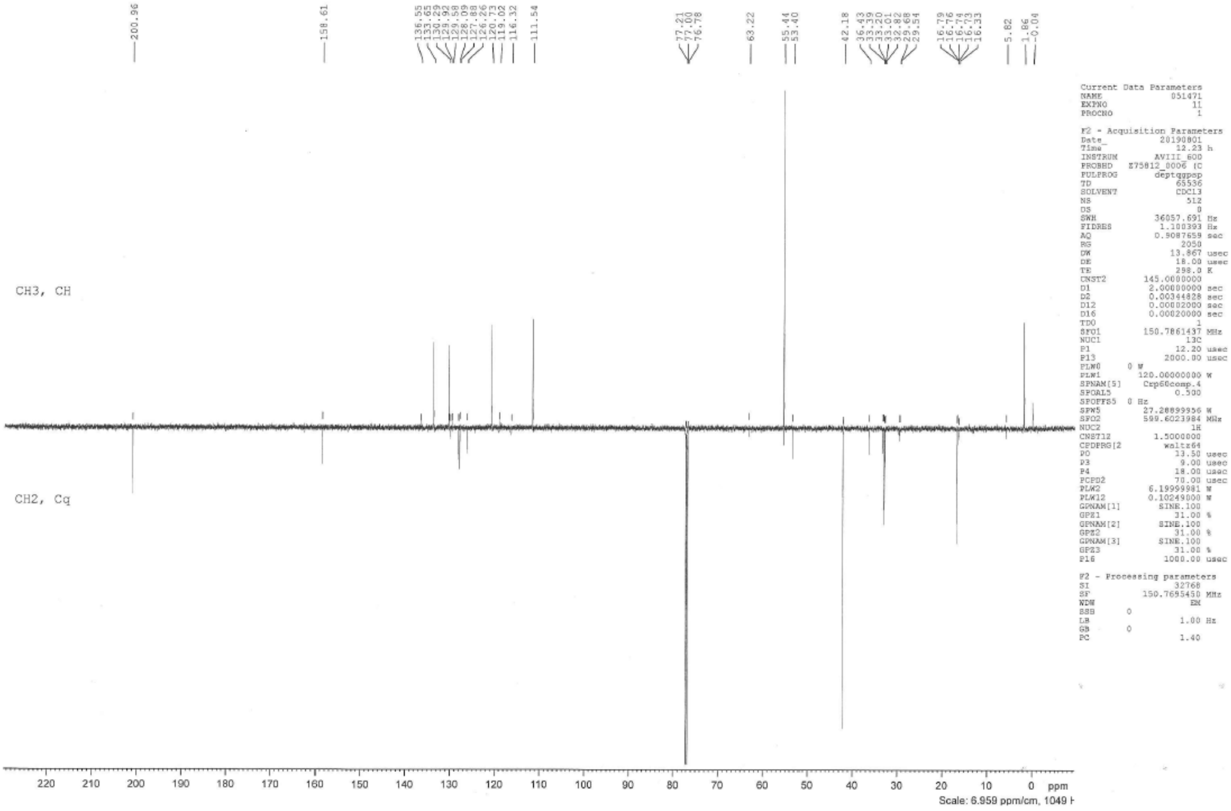

5,5,5-Trifluoro-1-(3-methoxyphenyl)pentan-1-one (5b)

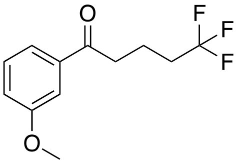

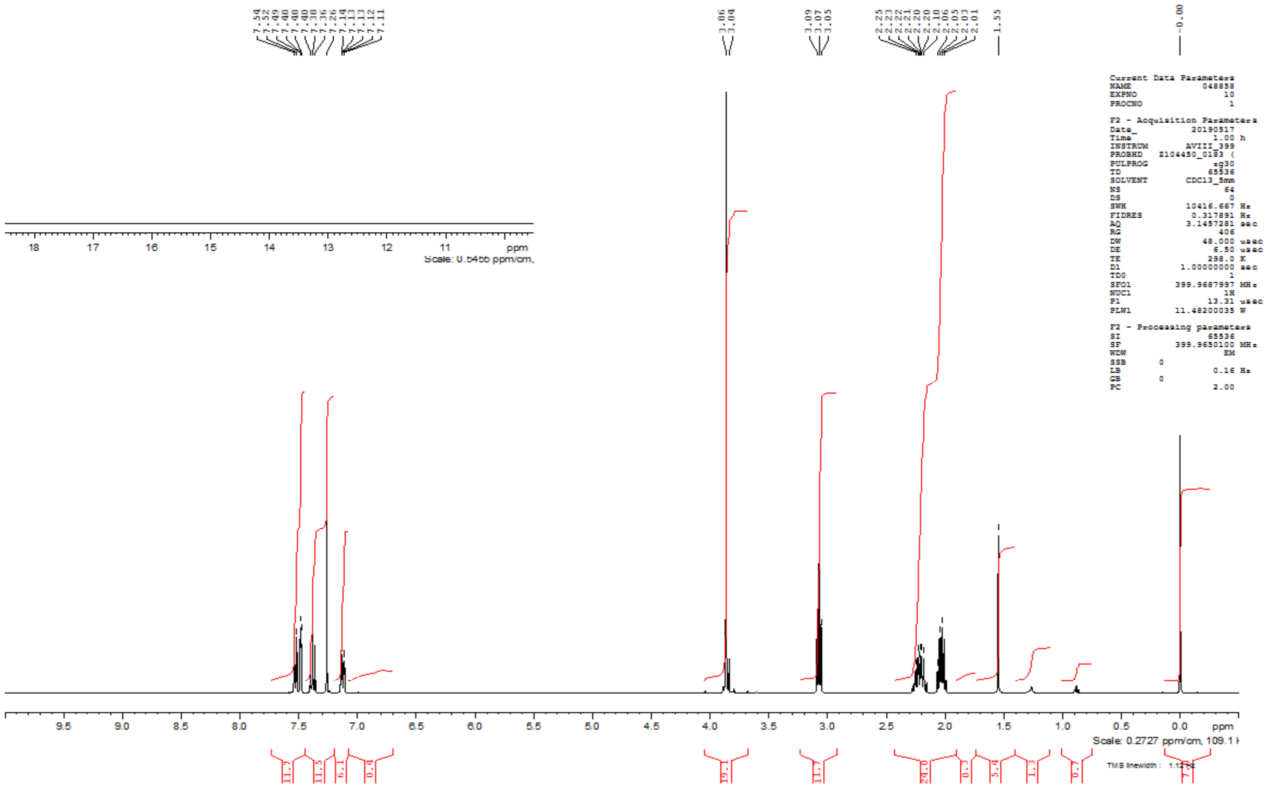

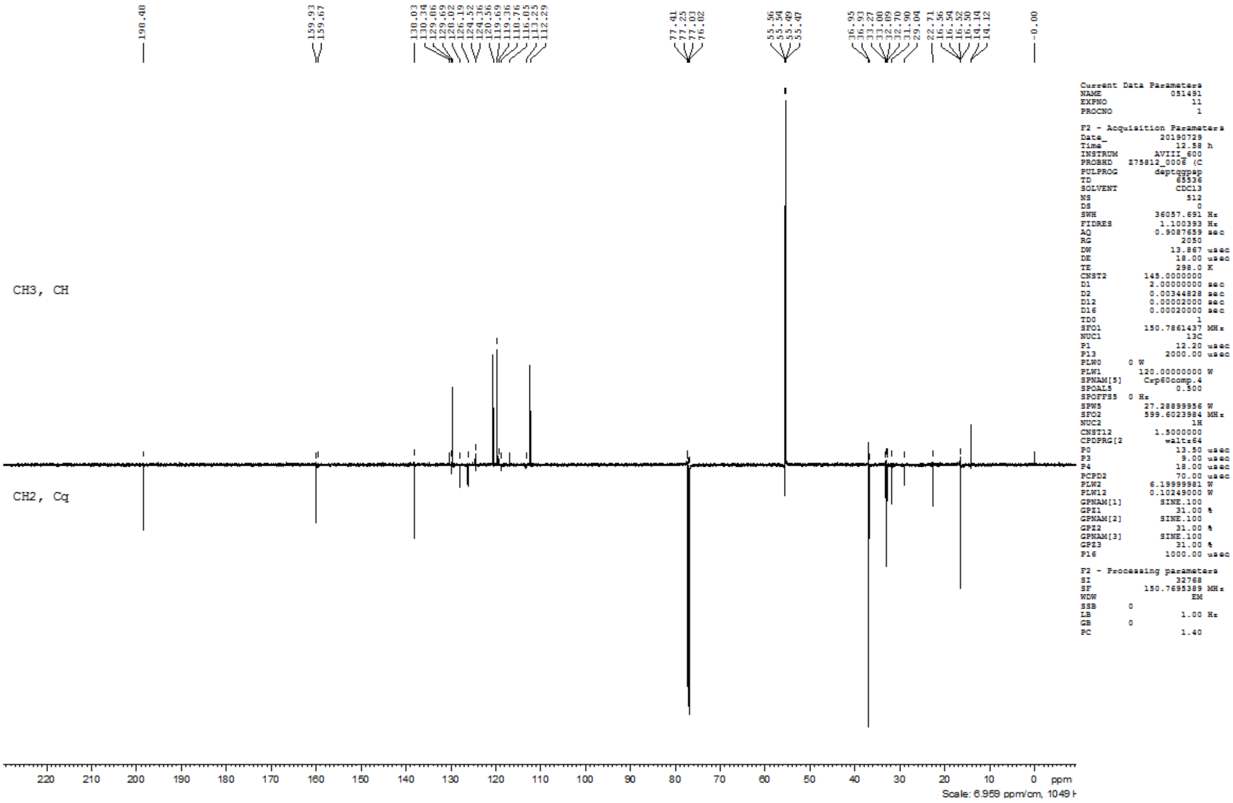

5,5,5-Trifluoro-1-phenyl-pentan-1-one (5c)

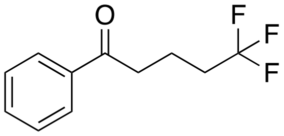

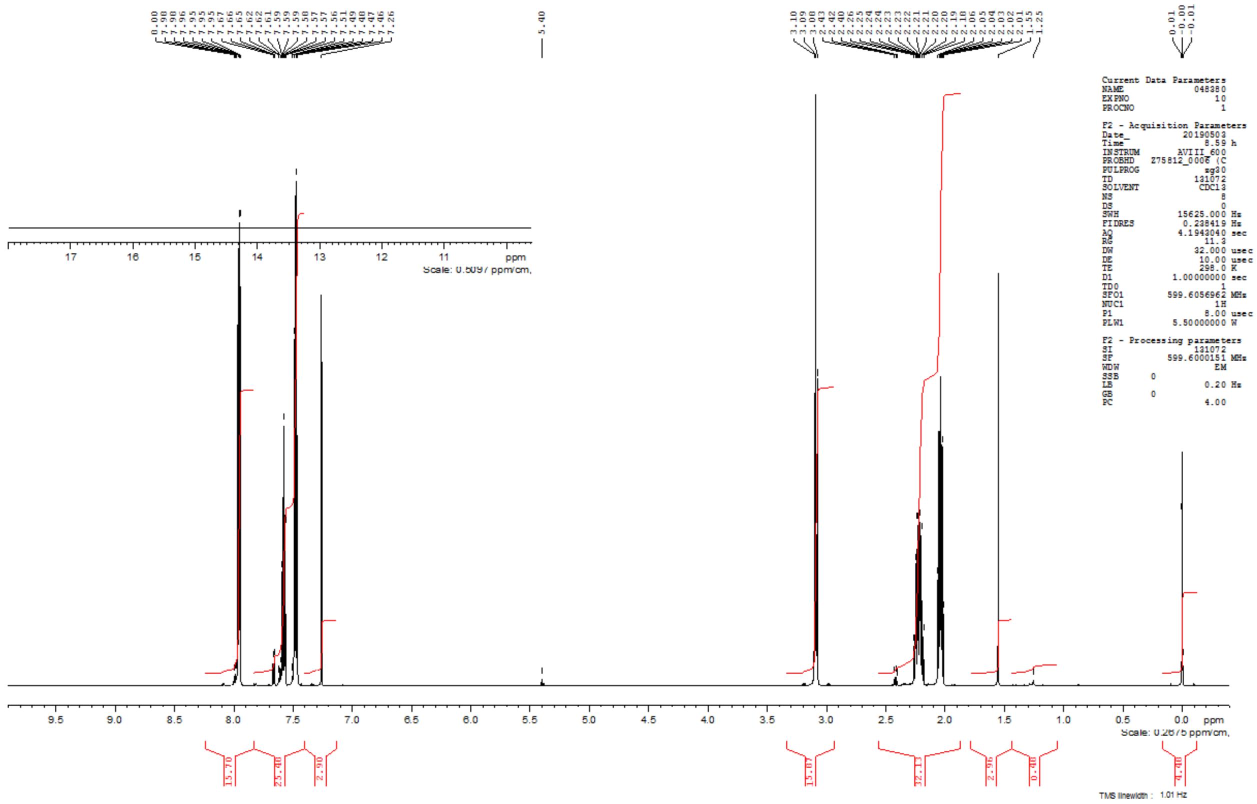

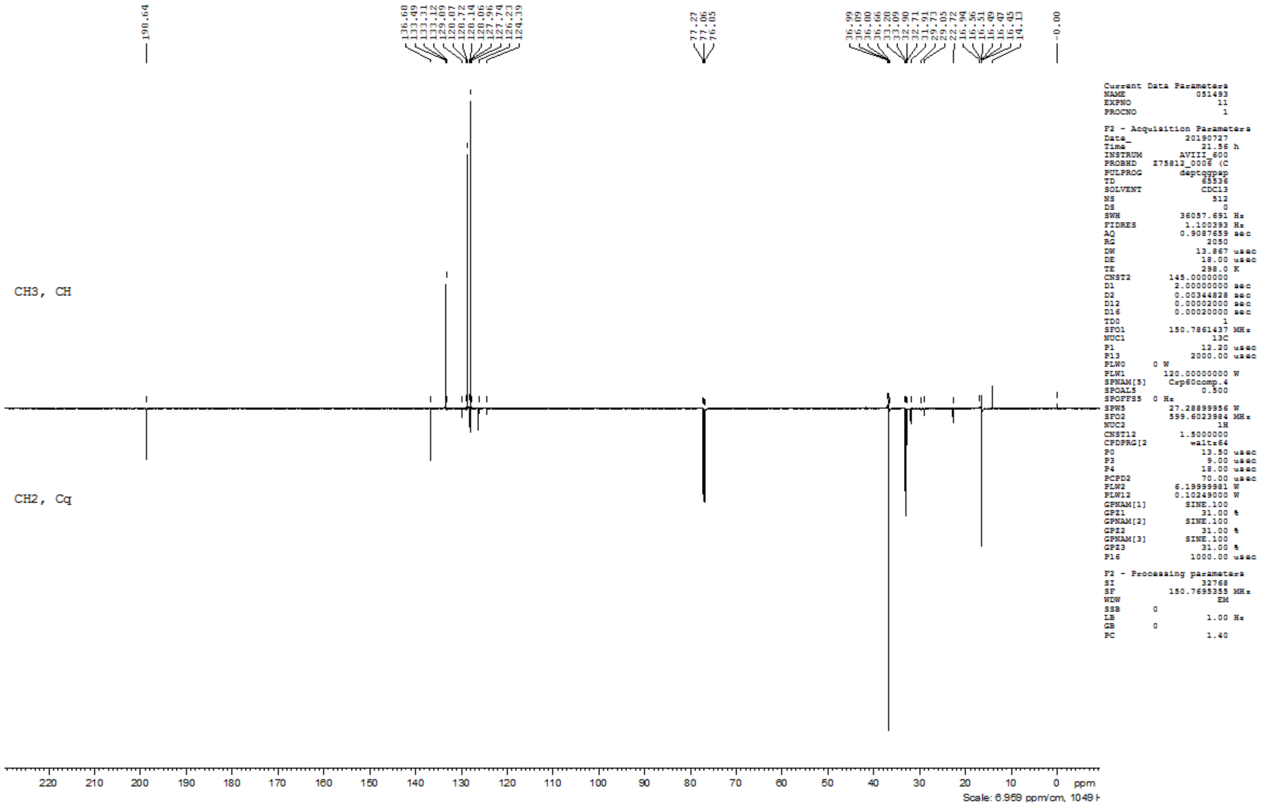

1-(4-Chlorophenyl)-5,5,5-trifluoro-pentan-1-one (5d)

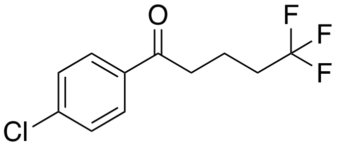

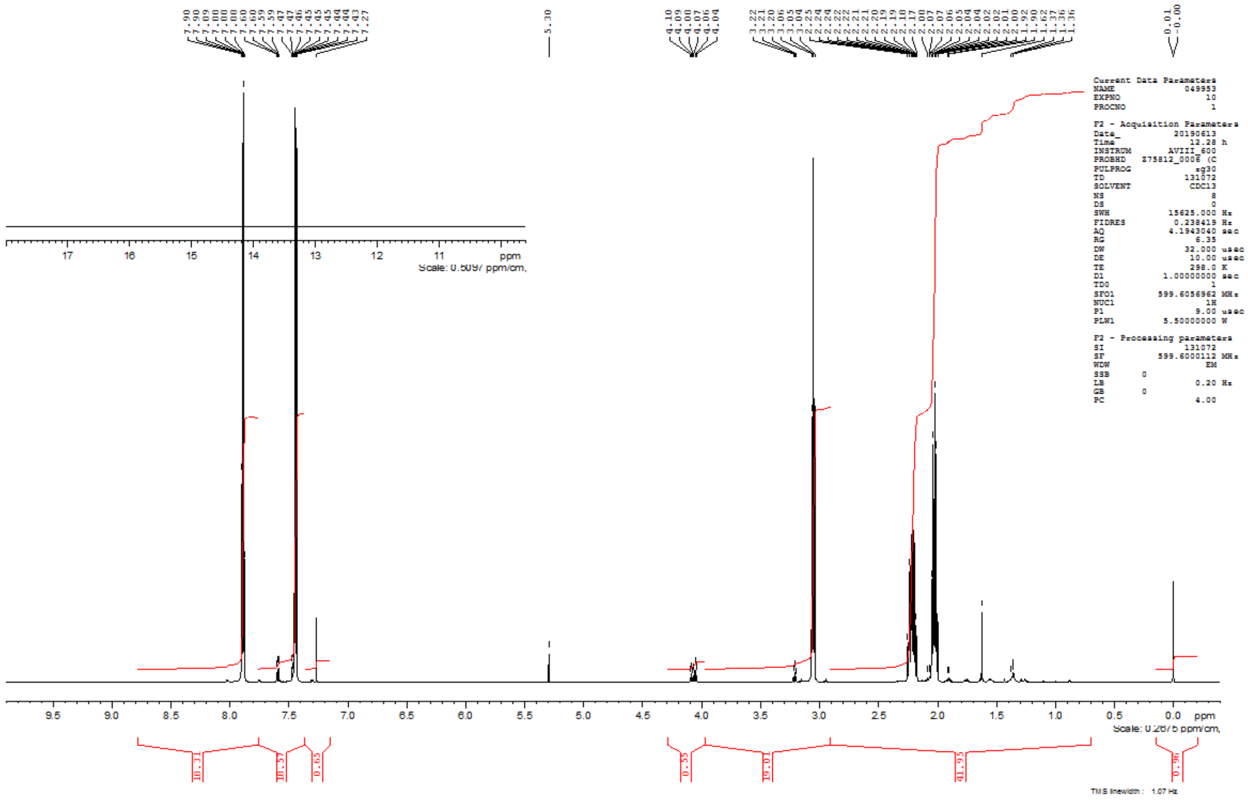

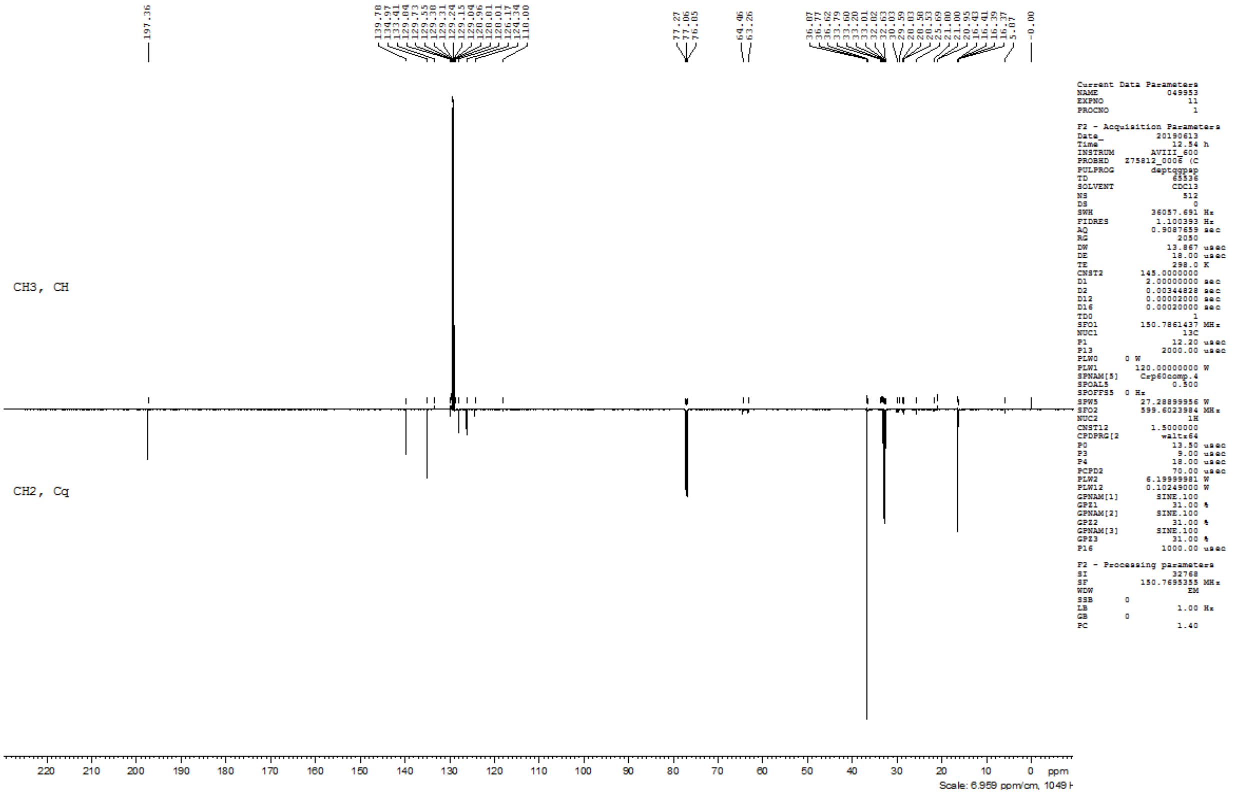

1-(2-Chlorophenyl)-5,5,5-trifluoro-pentan-1-one (5e)

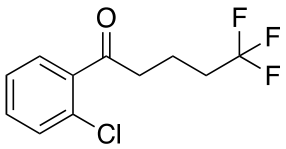

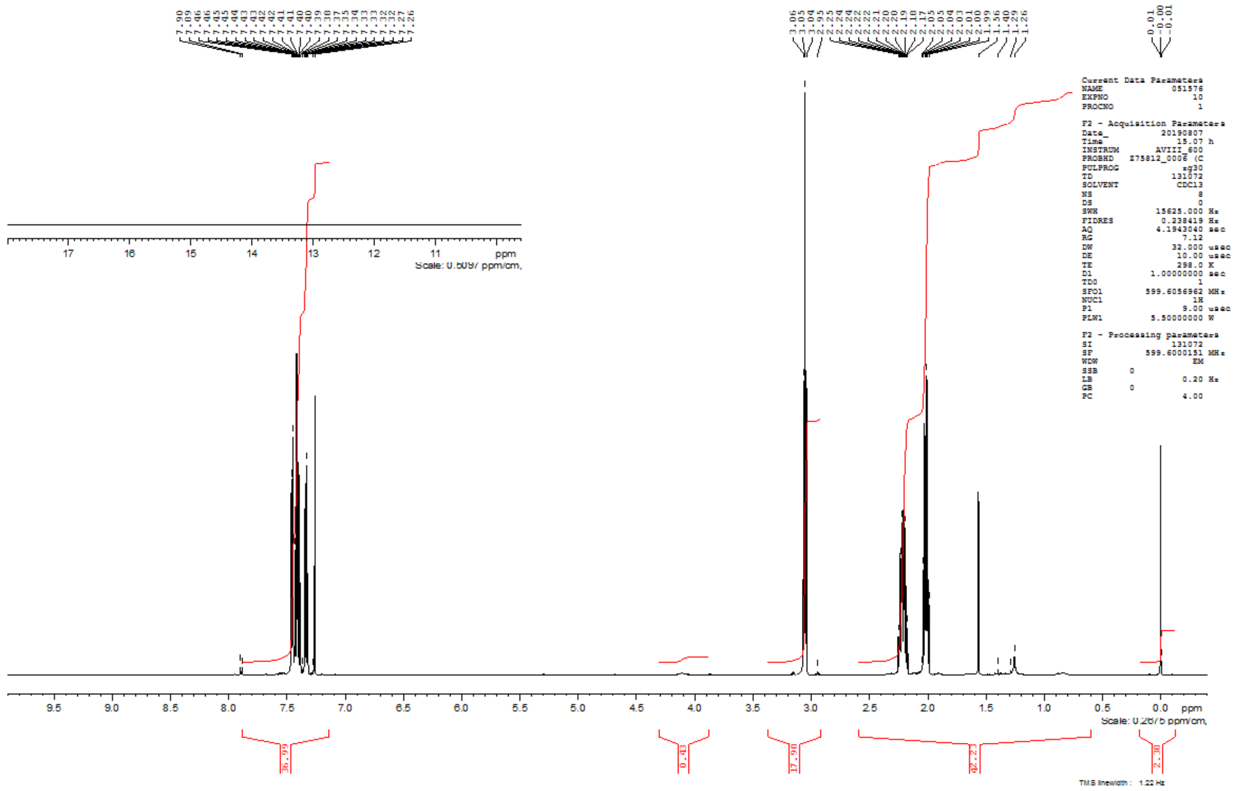

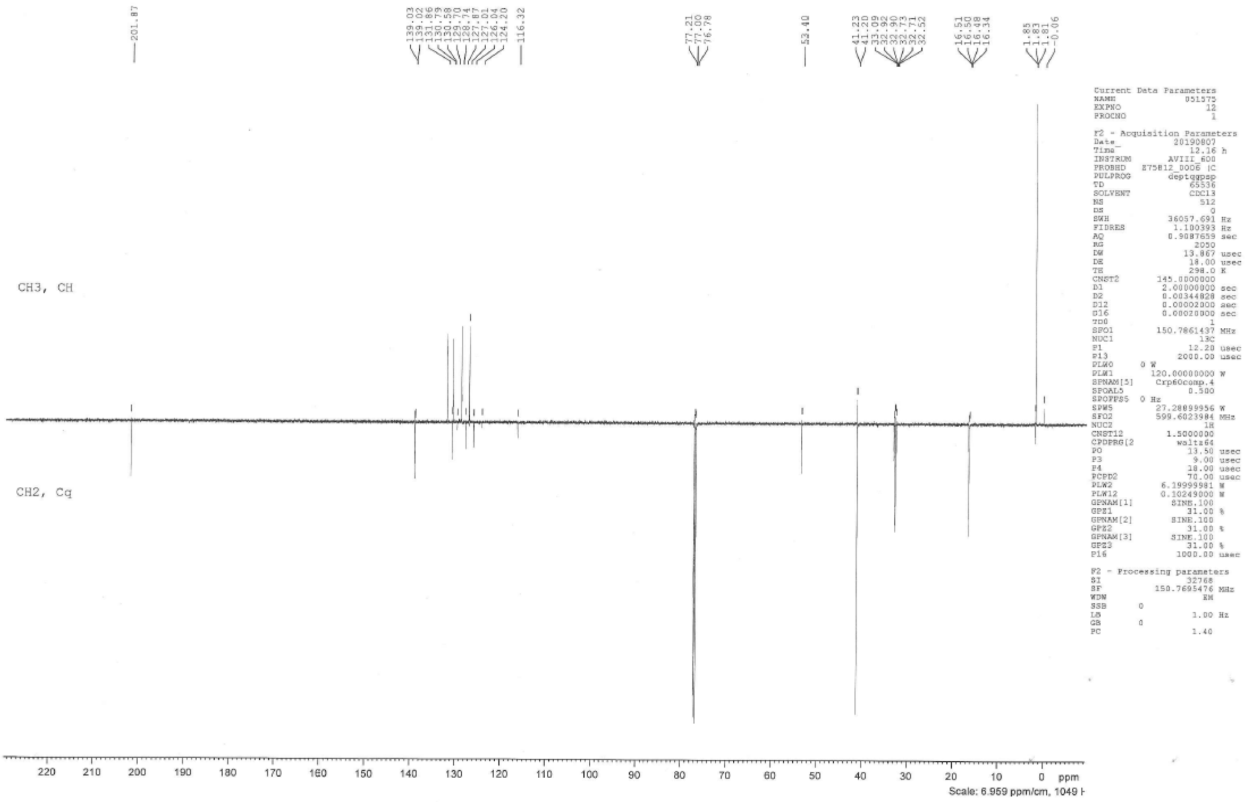

5,5,5-Trifluoro-1-(*p*-tolyl)pentan-1-one (5f)

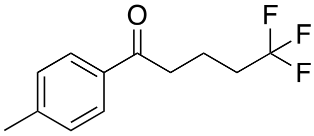

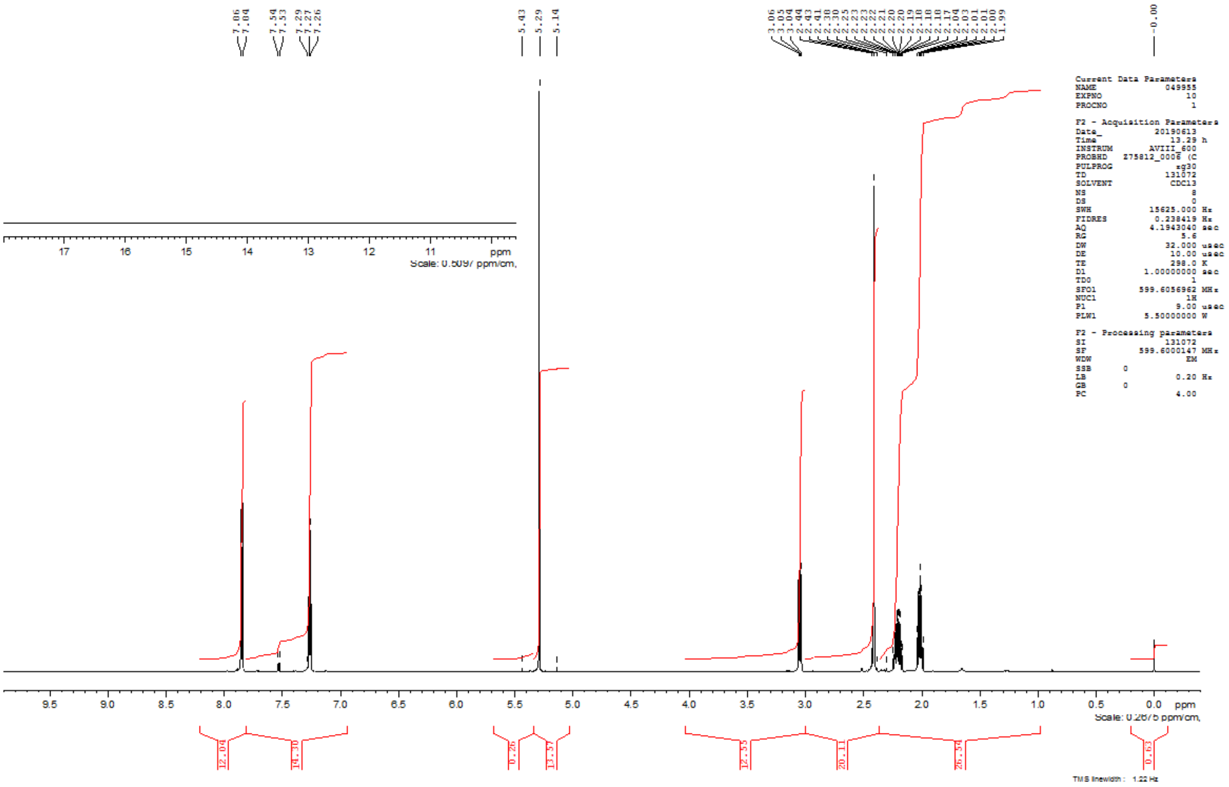

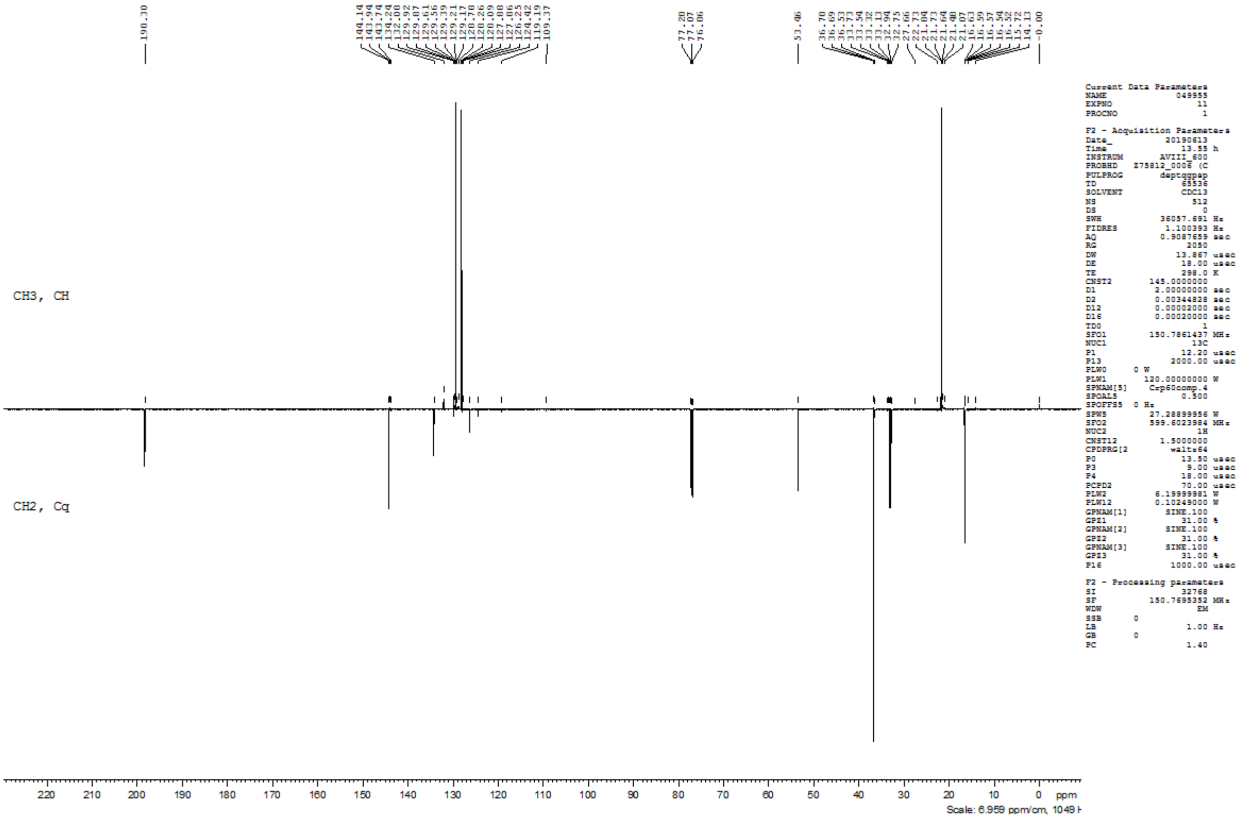

1-(4-Chlorophenyl)-5,5,5-trifluoro-pentan-1-one (5g)

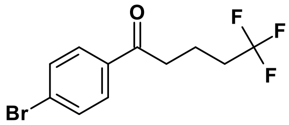

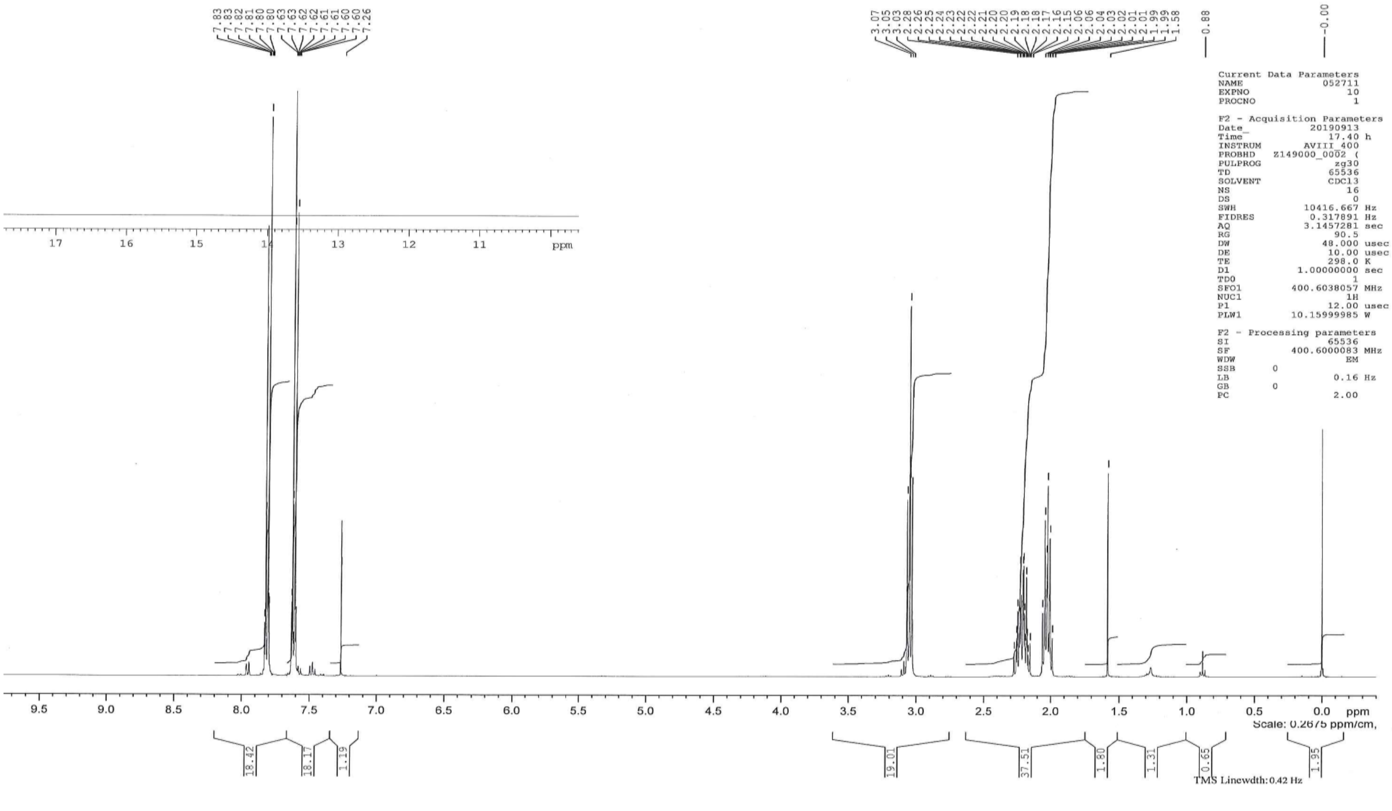

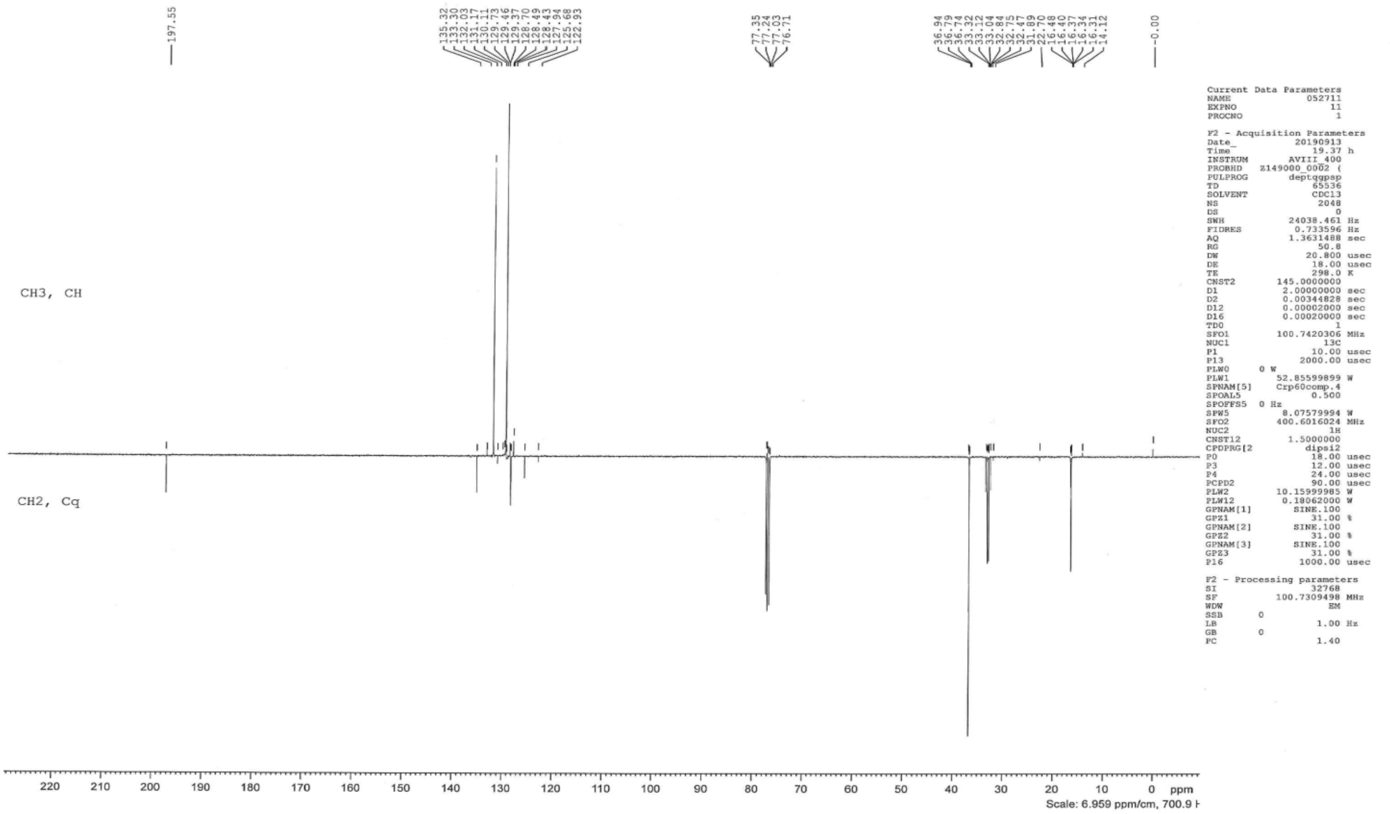

5,5,5-Trifluoro-1-(2-naphthyl)pentan-1-one (5h)

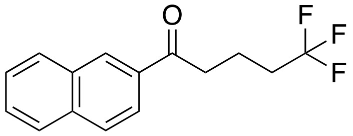

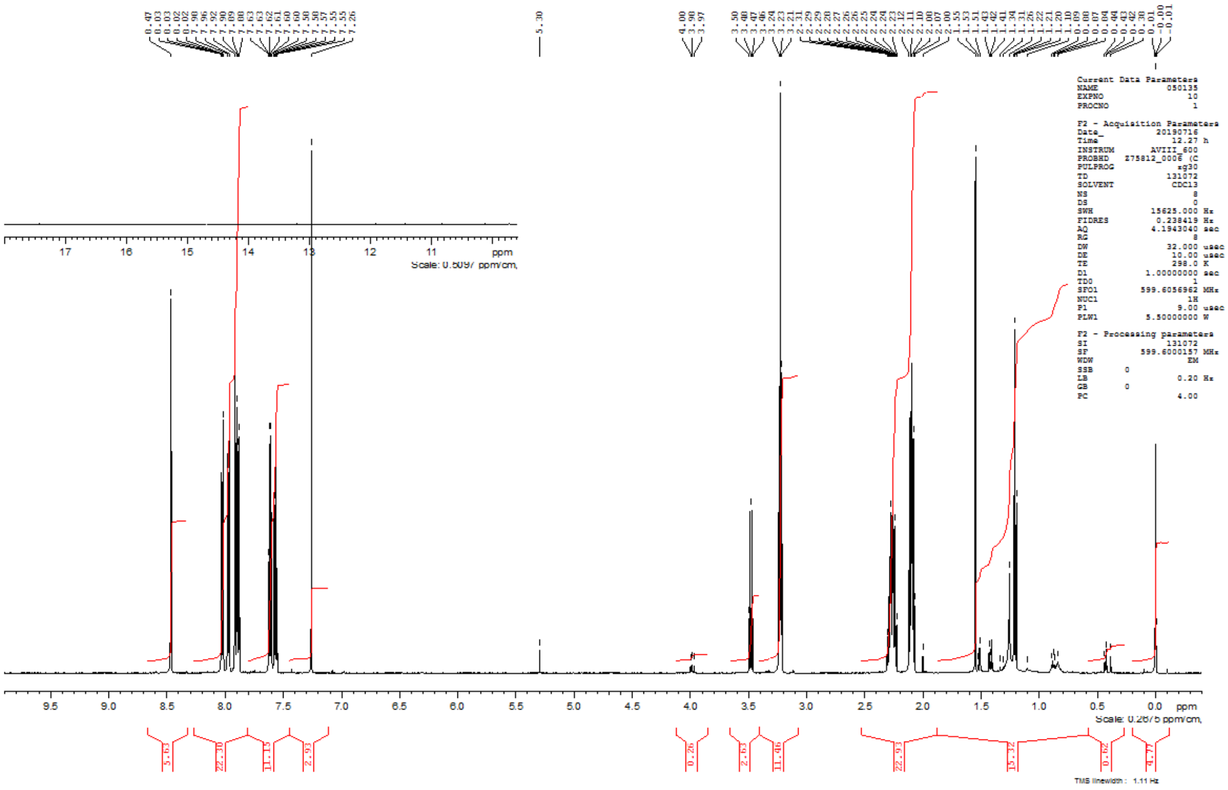

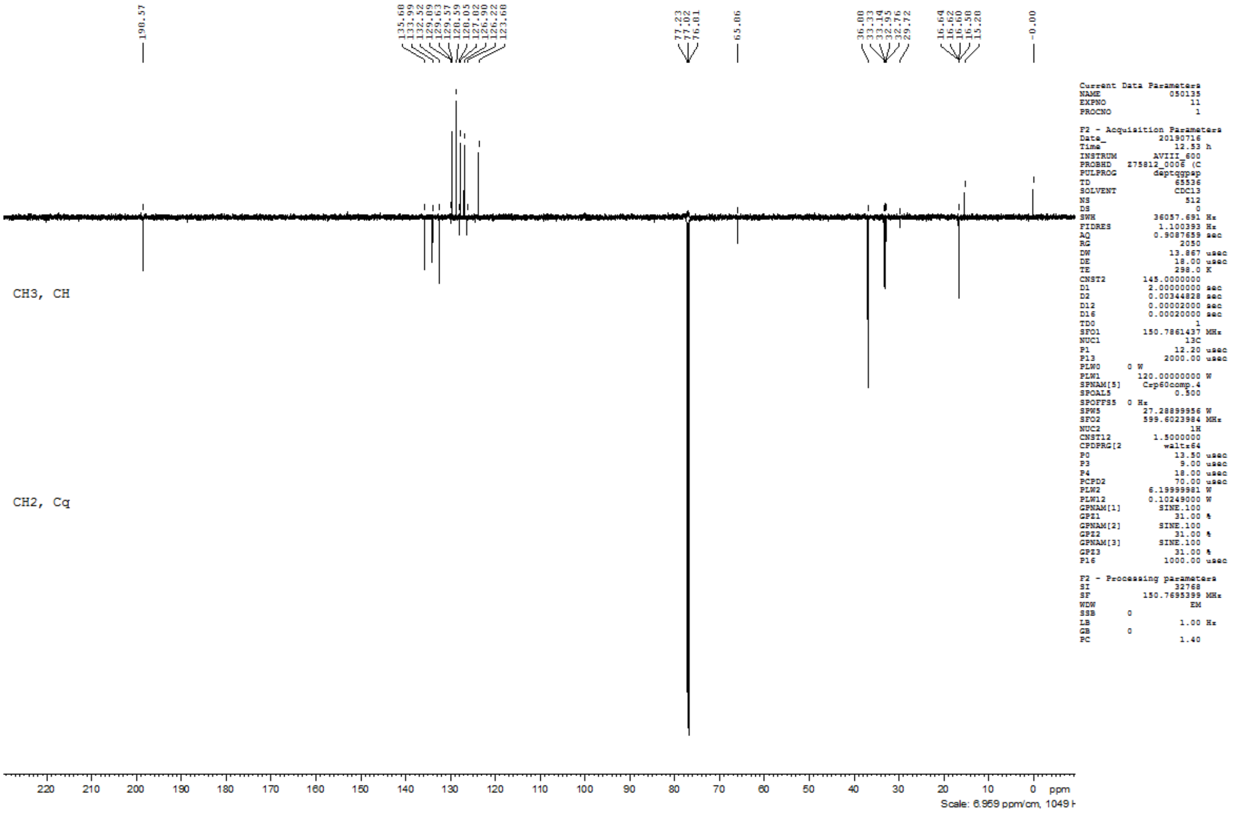

5,5,5-Trifluoro-1-(2-pyridyl)pentan-1-one (5i)

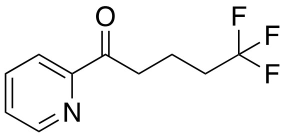

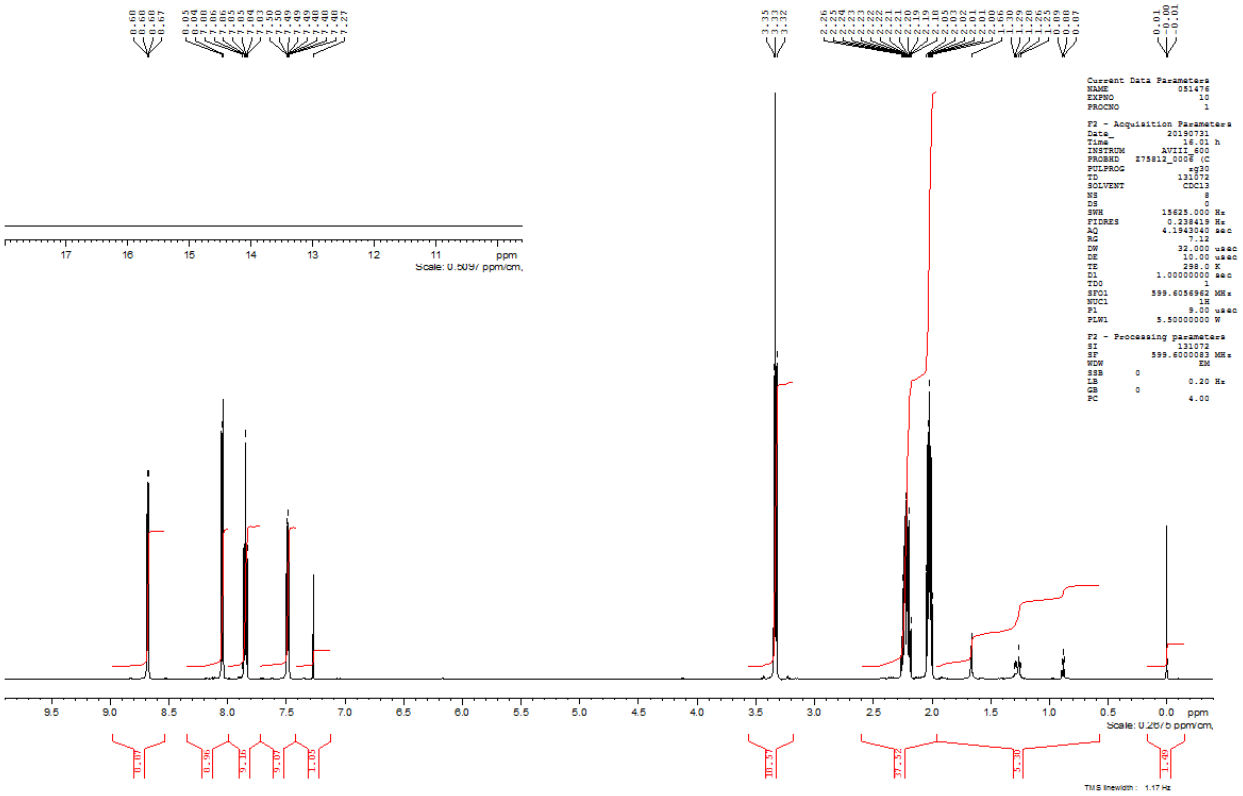

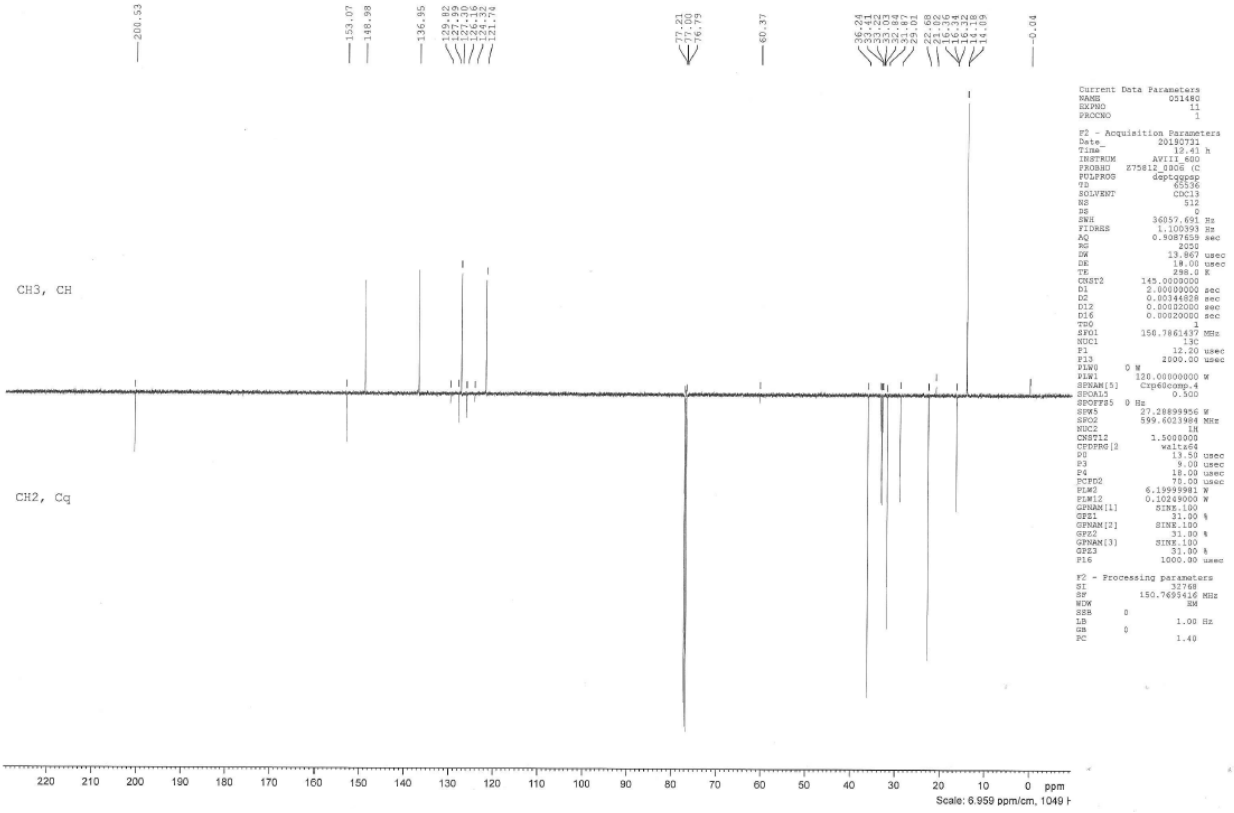

5,5,5-Trifluoro-1-(3-pyridyl)pentan-1-one (5j)

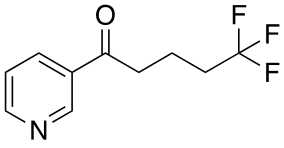

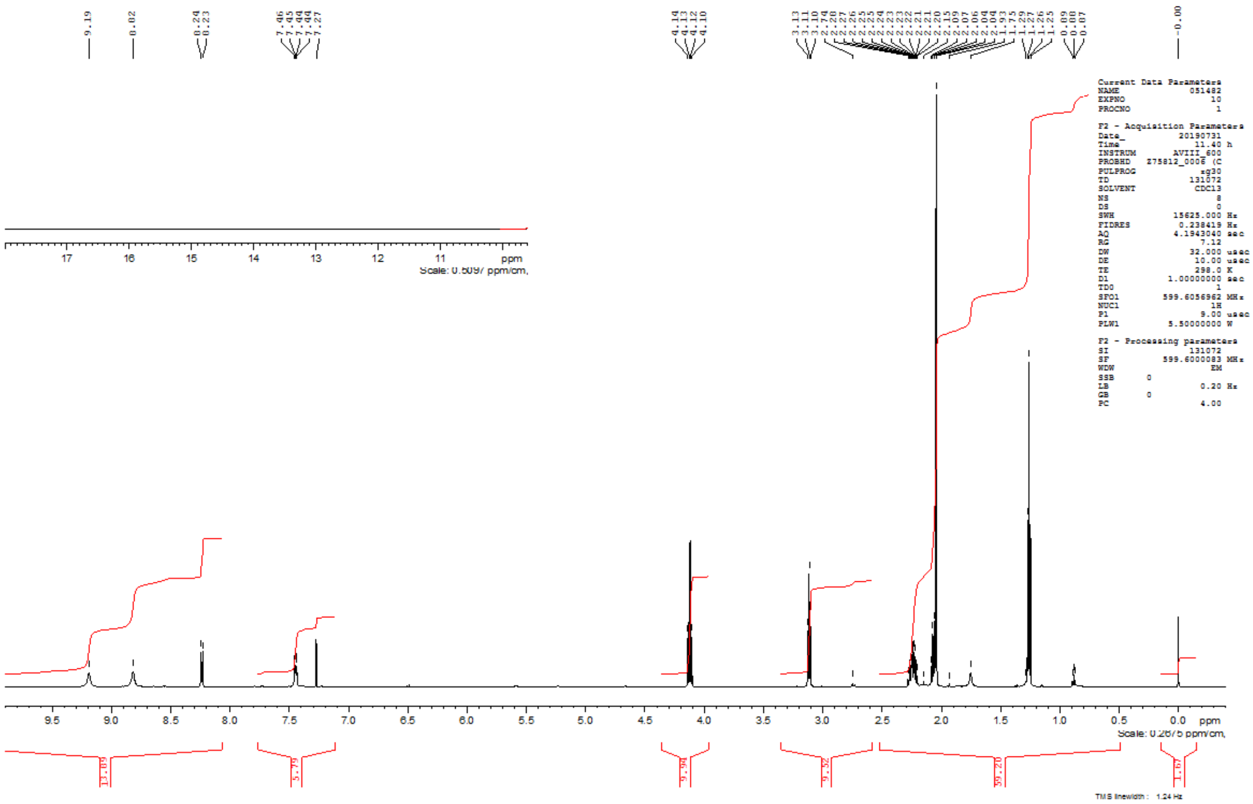

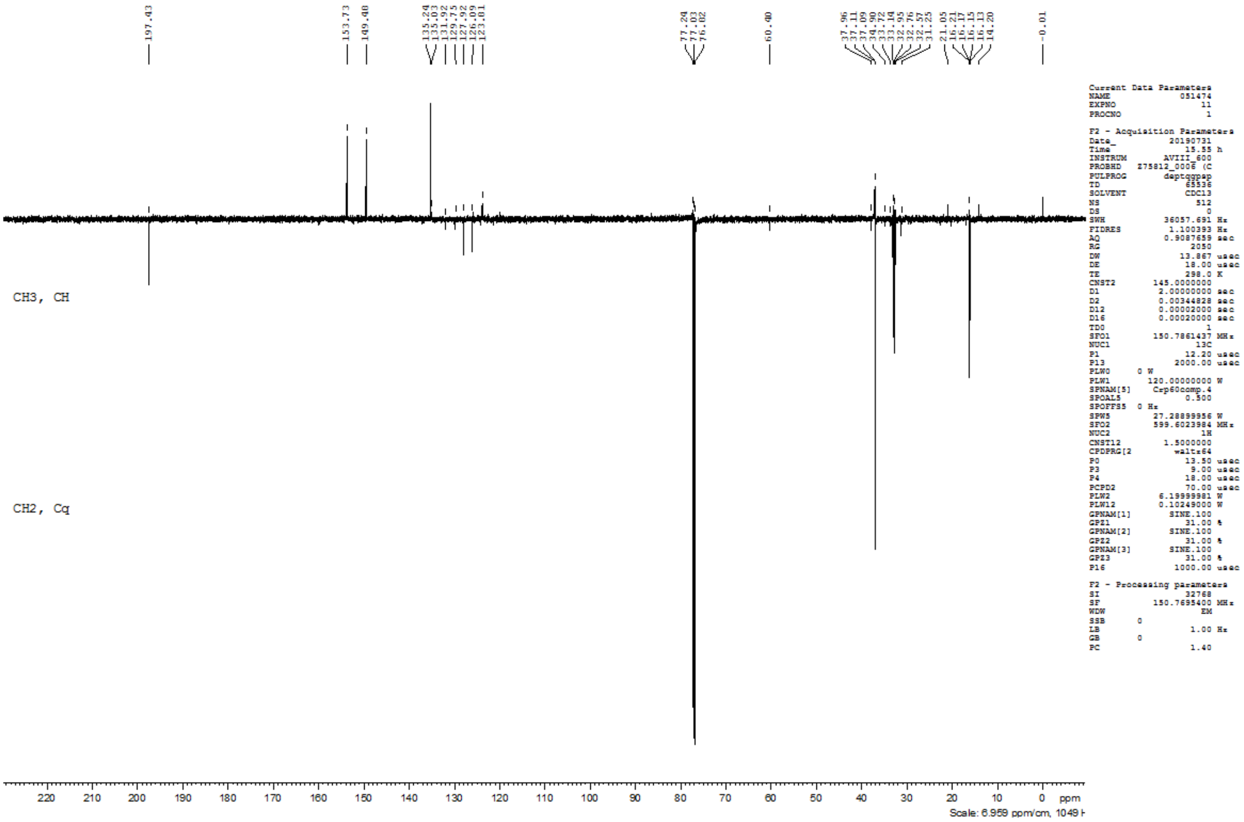

5,5,5-Trifluoro-1-(4-pyridyl)pentan-1-one (5k)

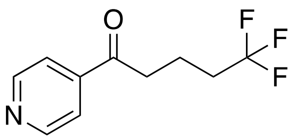

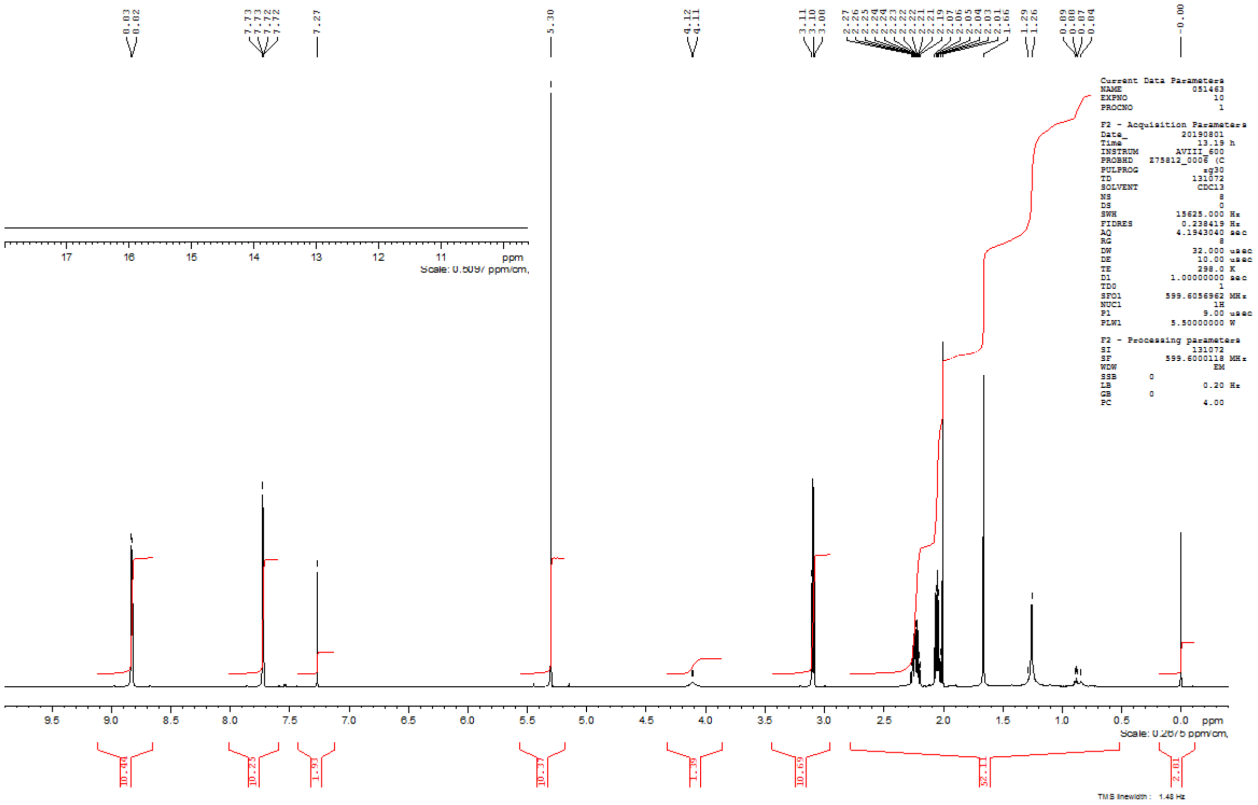

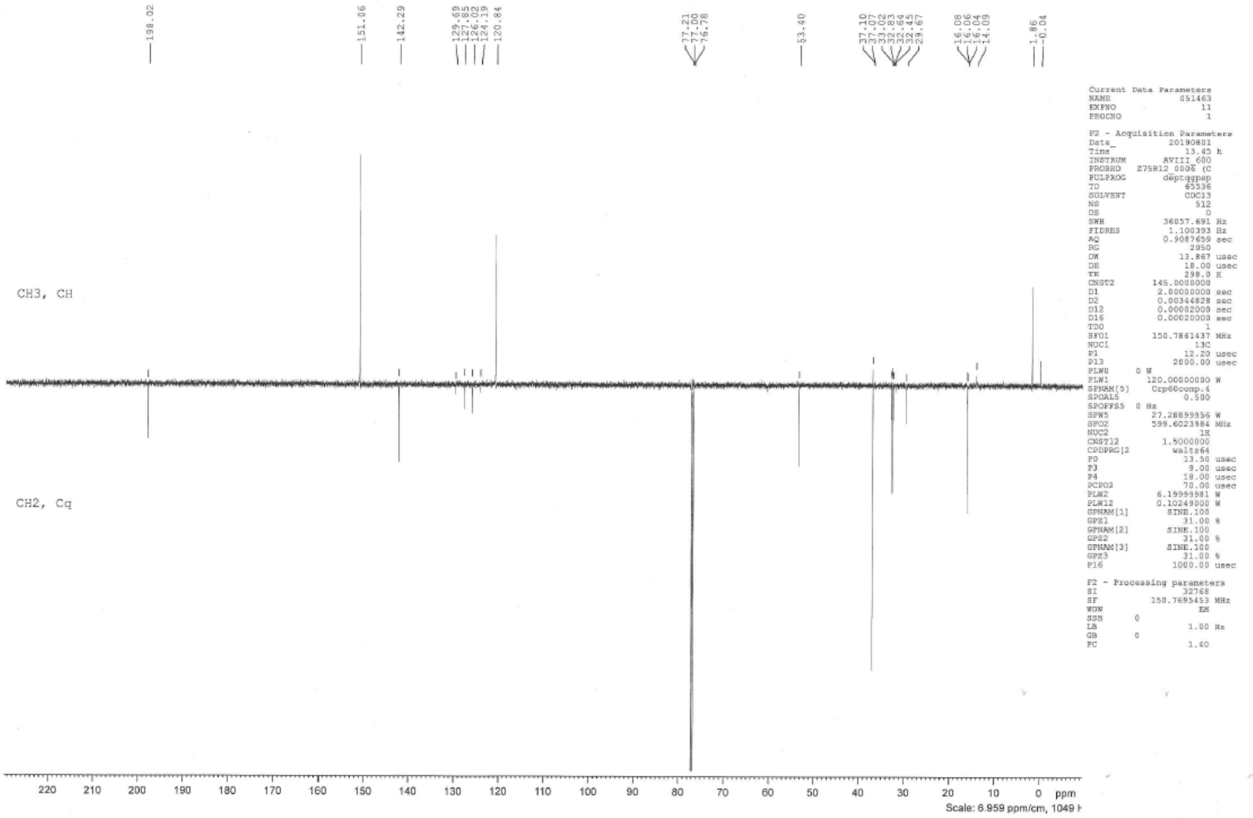

4,4,4-Trifluoro-1-phenyl-butan-1-one (5l)

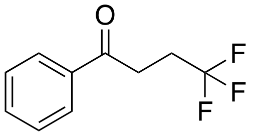

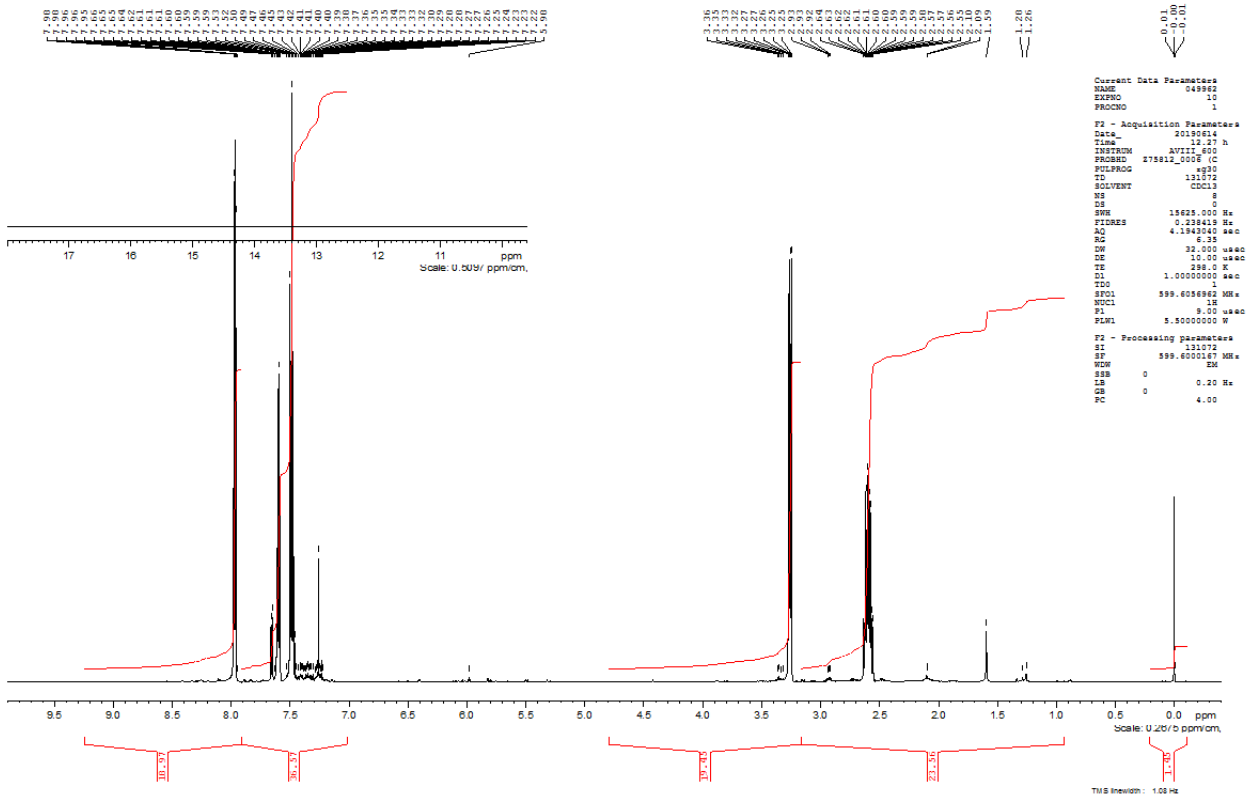

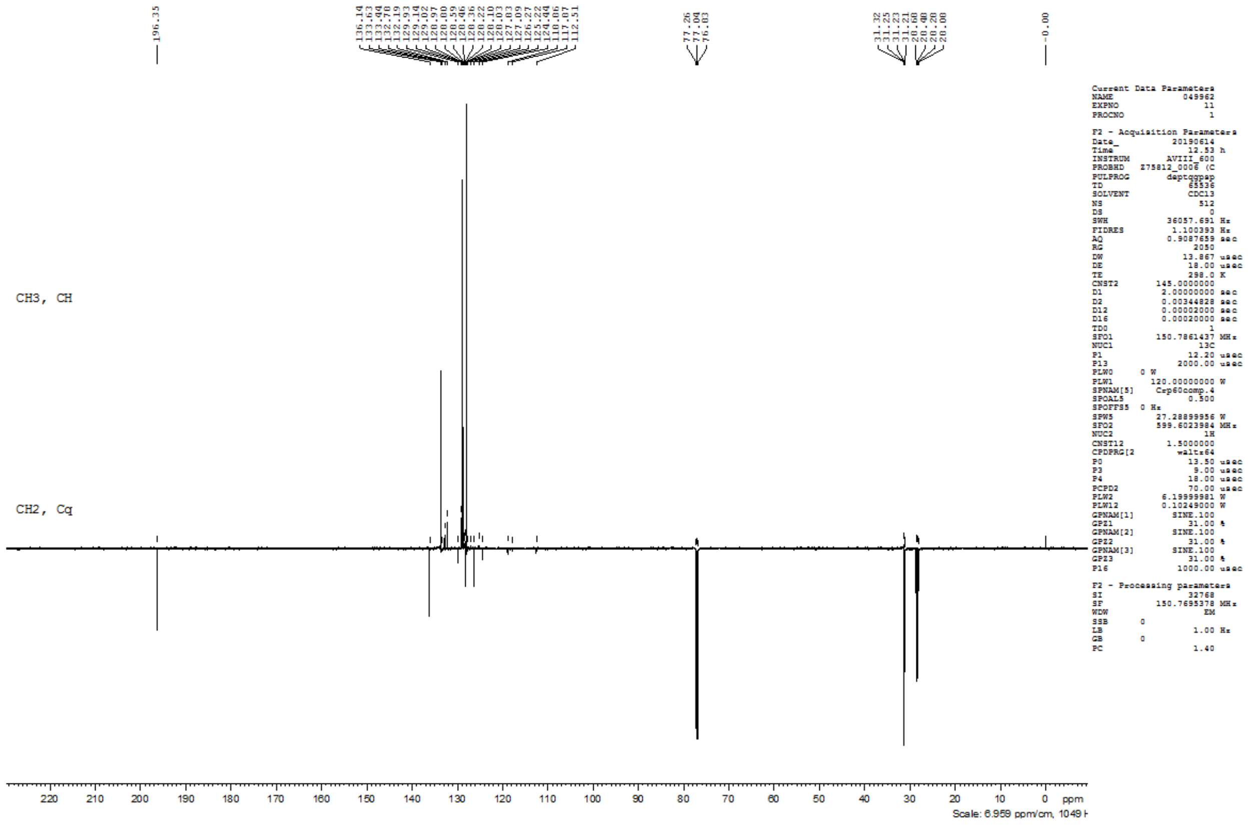

5,5,5-Trifluoro-1-(4-methoxyphenyl)pentan-1-one (5m)

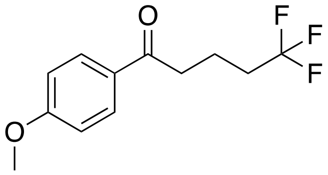

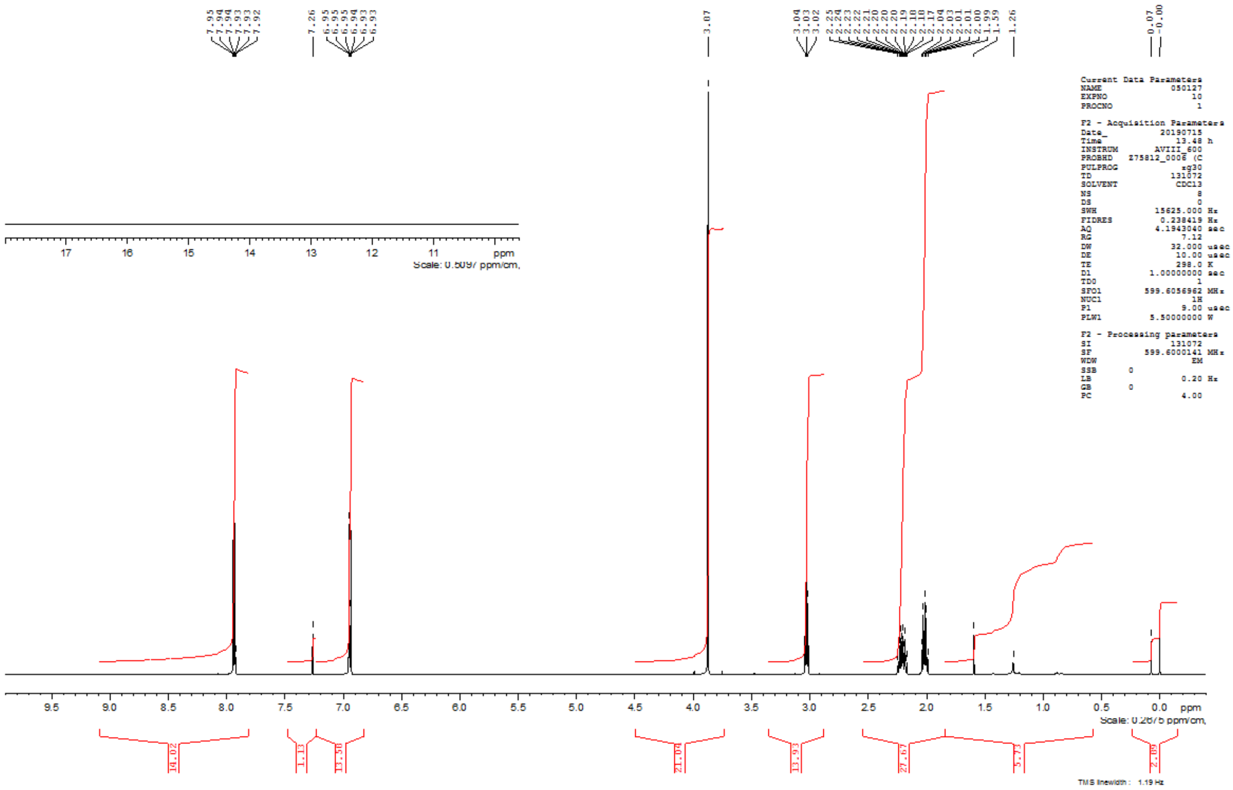

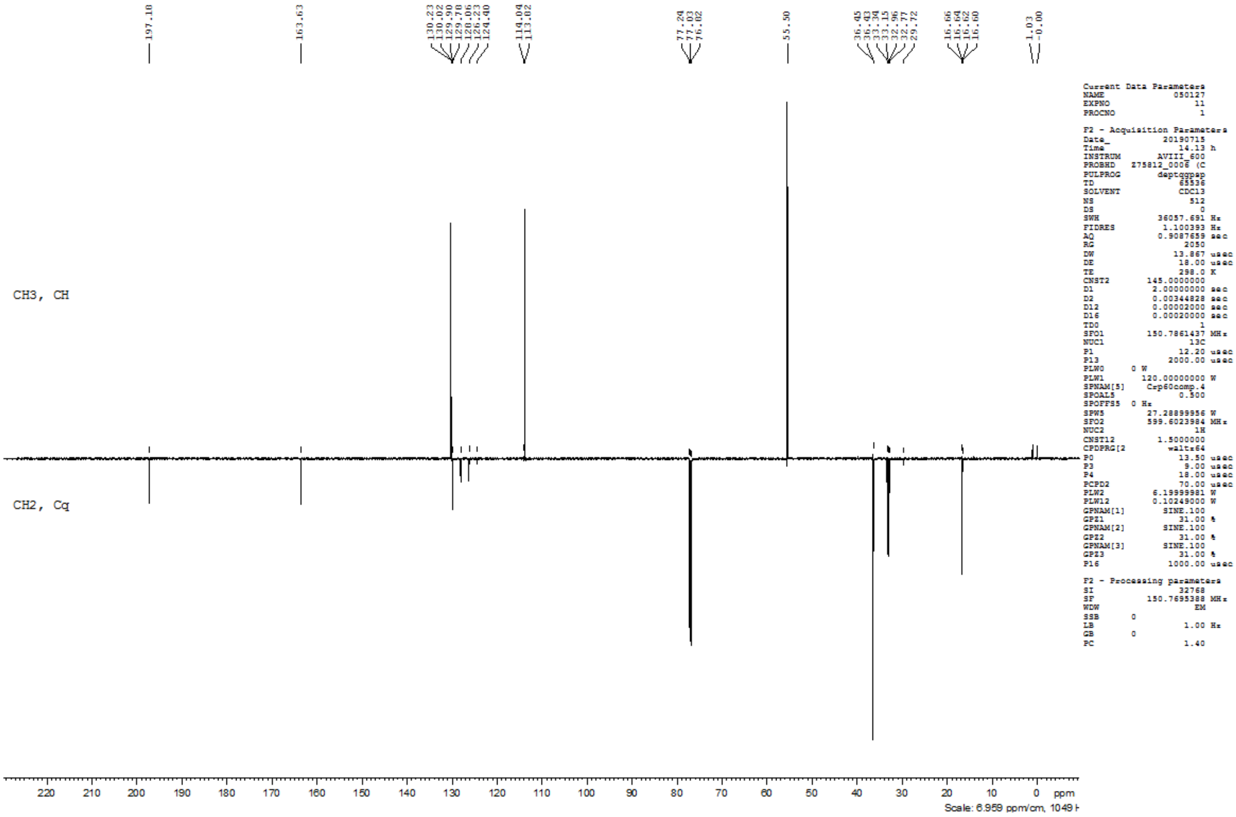

5,5-Dichloro-1-(2-methoxyphenyl)pent-4-en-1-one (6a)

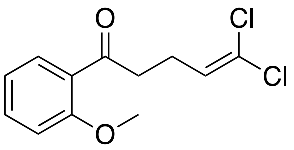

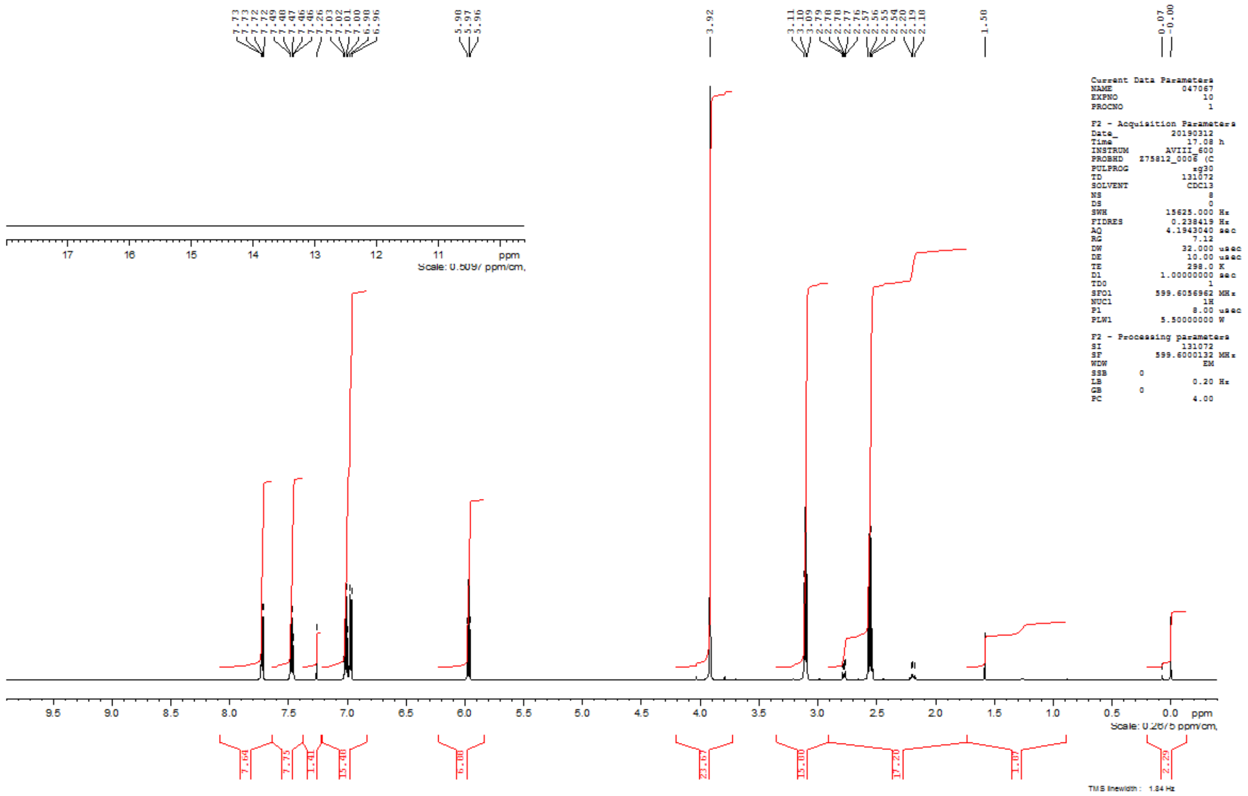

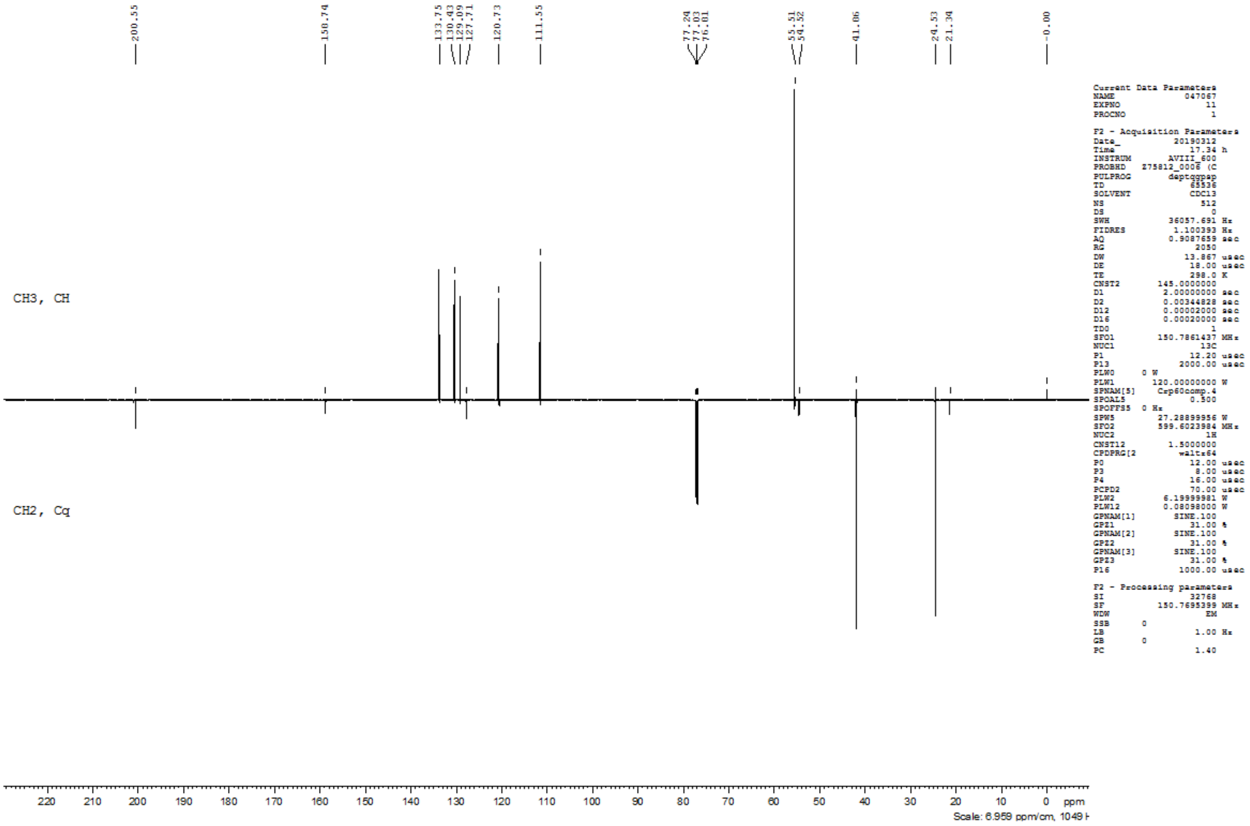

5,5-Dichloro-1-(3-methoxyphenyl)pent-4-en-1-one (6b)

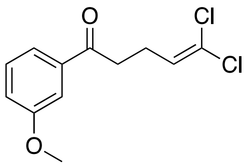

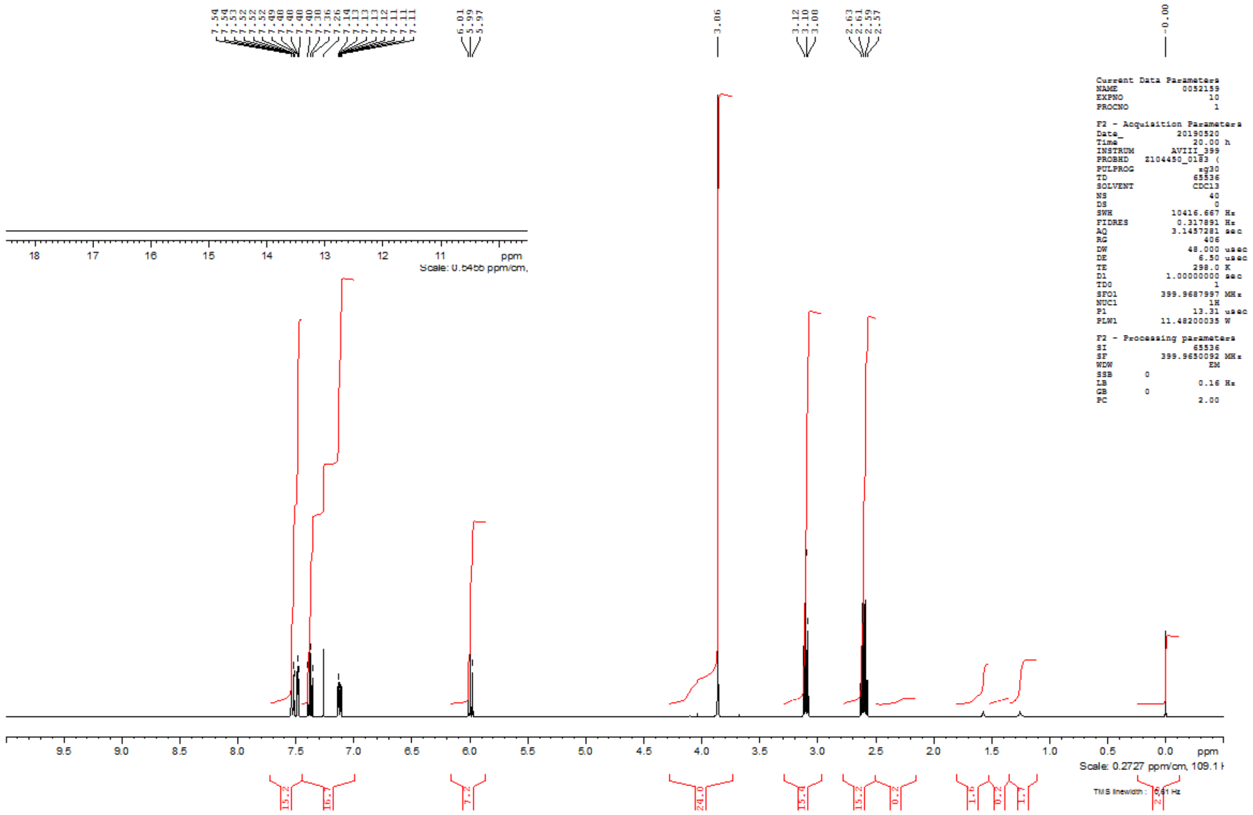

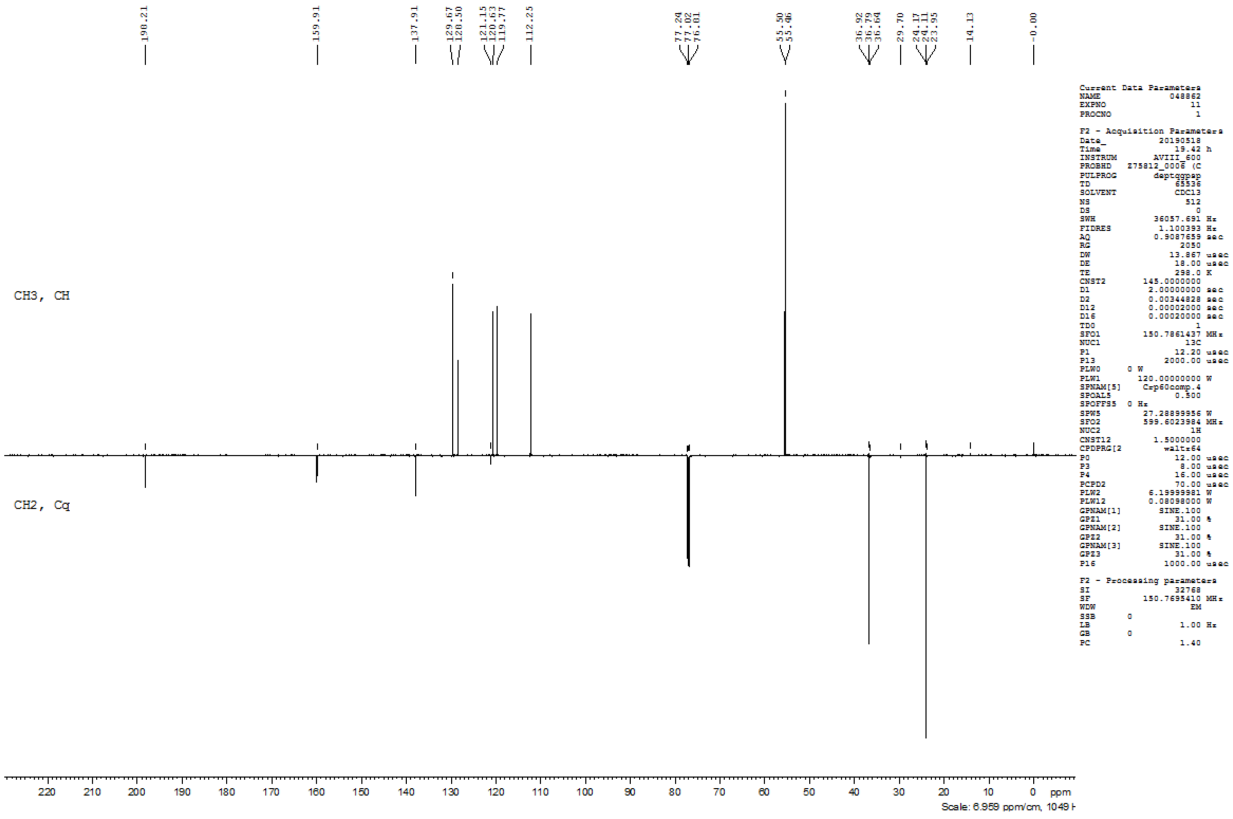

5,5,5-Trichloro-1-(3-methoxyphenyl)pentan-1-one (8b)

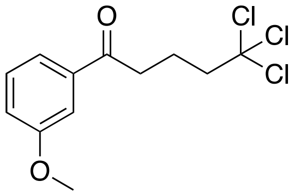

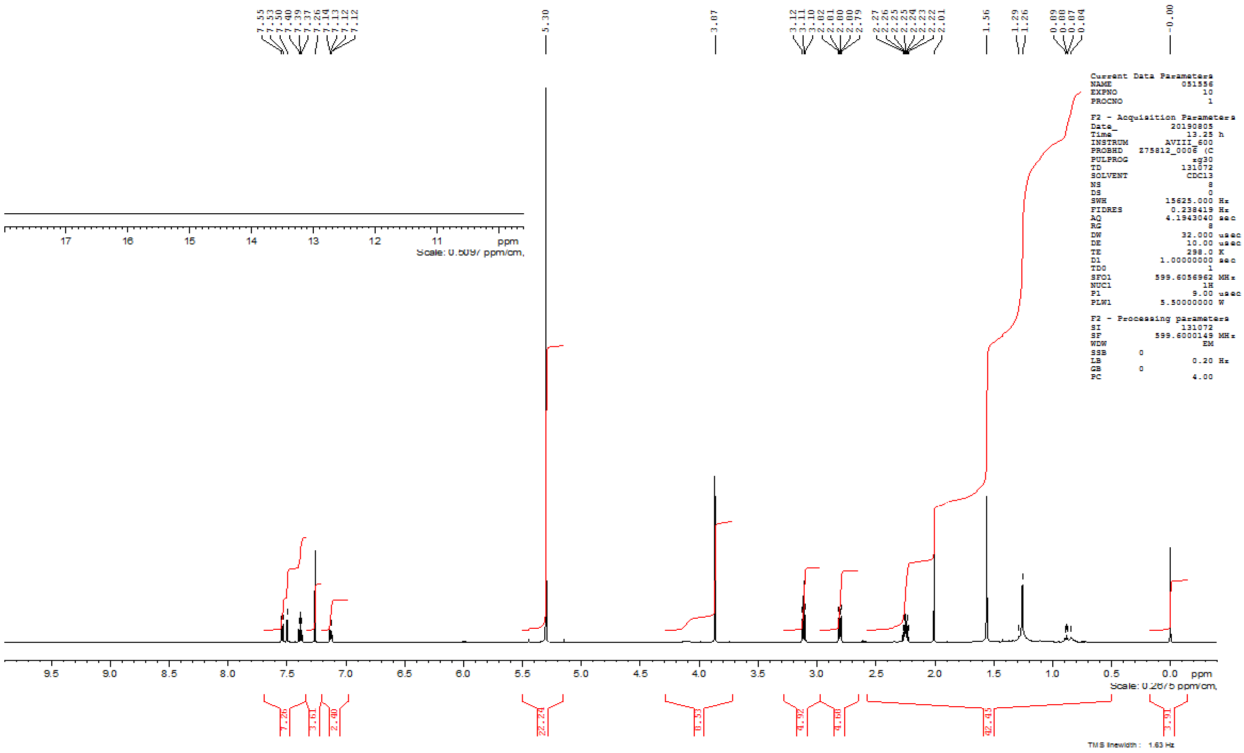

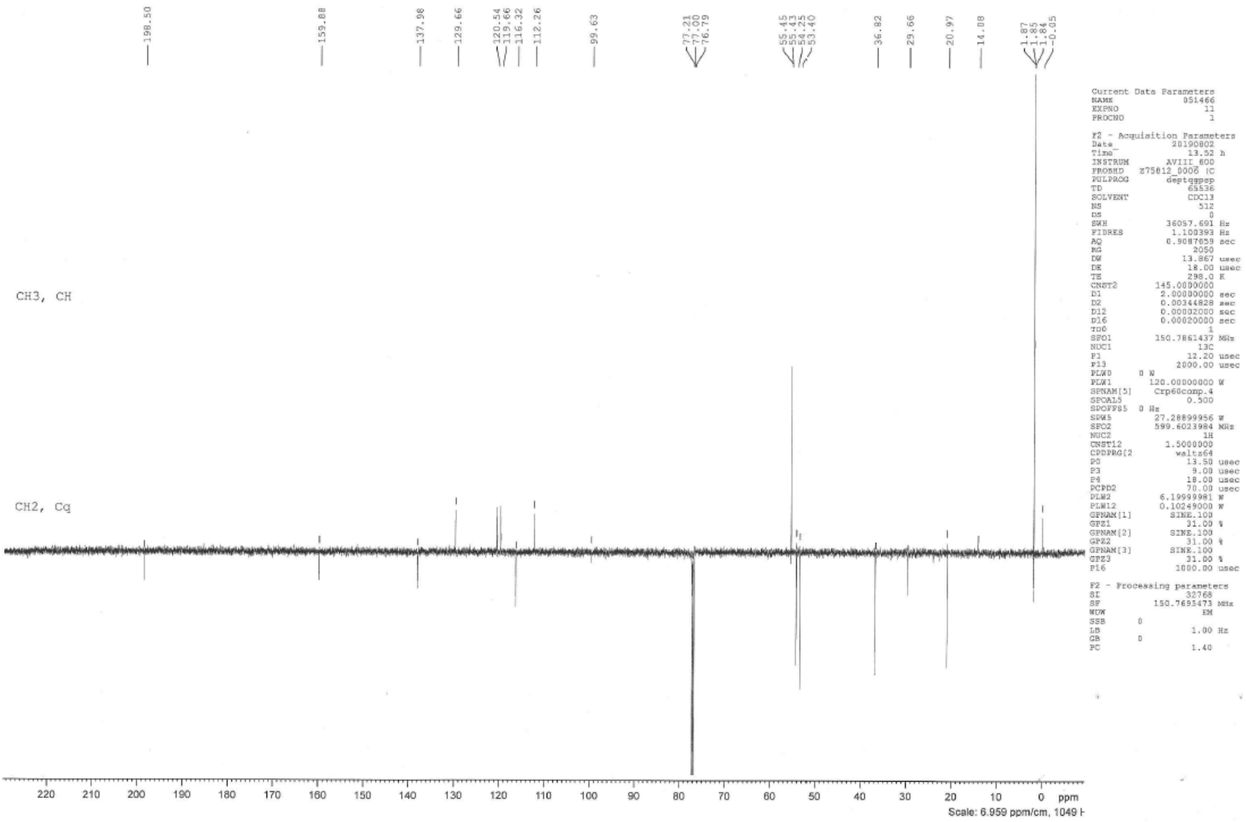

5,5-Dichloro-1-phenyl-pent-4-en-1-one (6c)

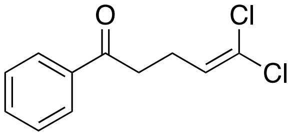

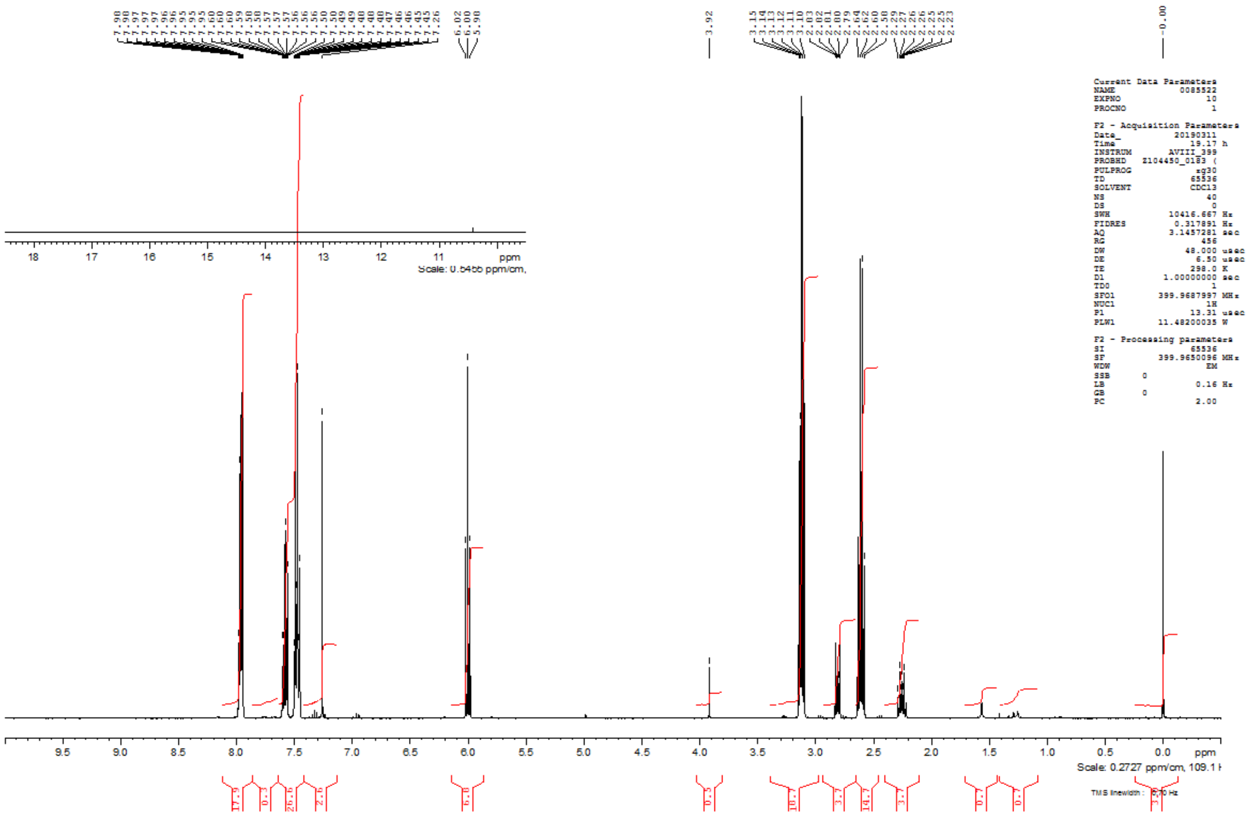

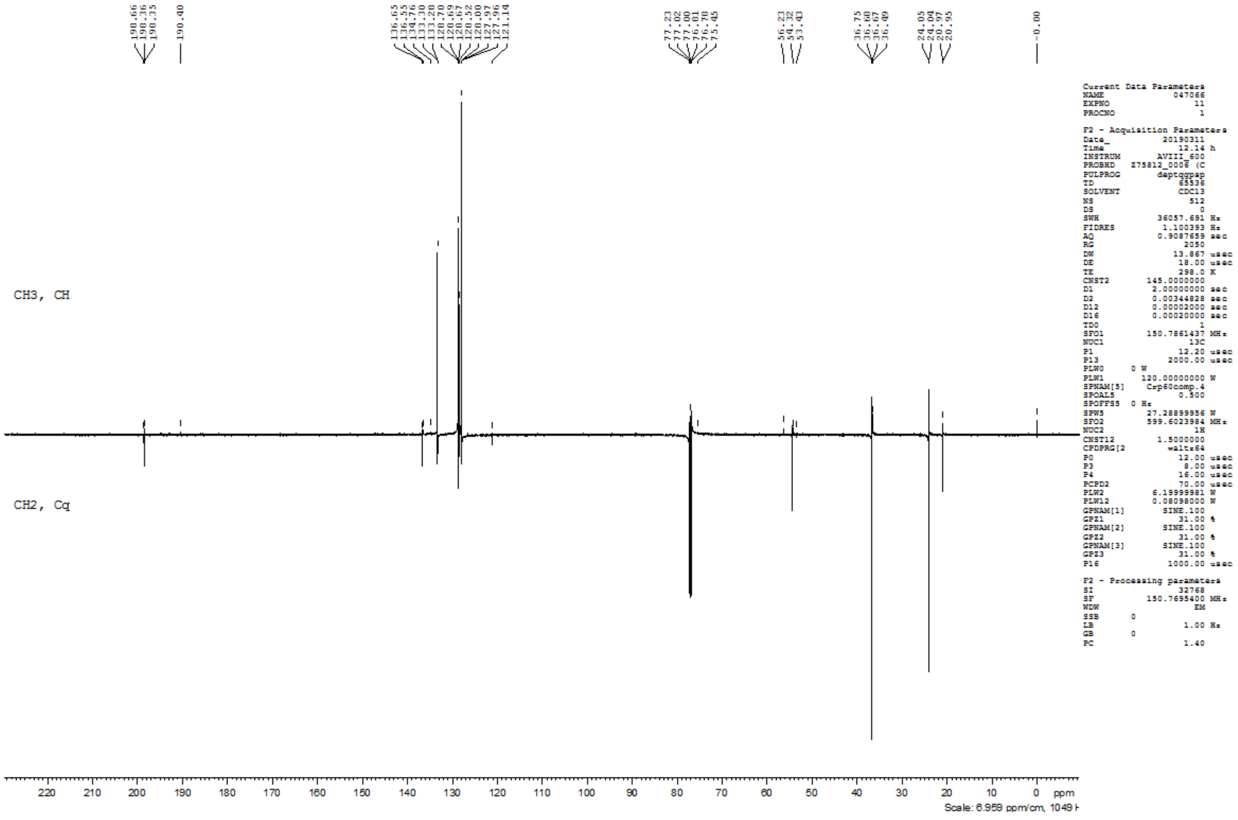

5,5-Dichloro-1-(4-chlorophenyl)pent-4-en-1-one (6d)

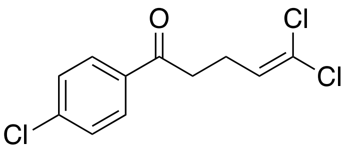

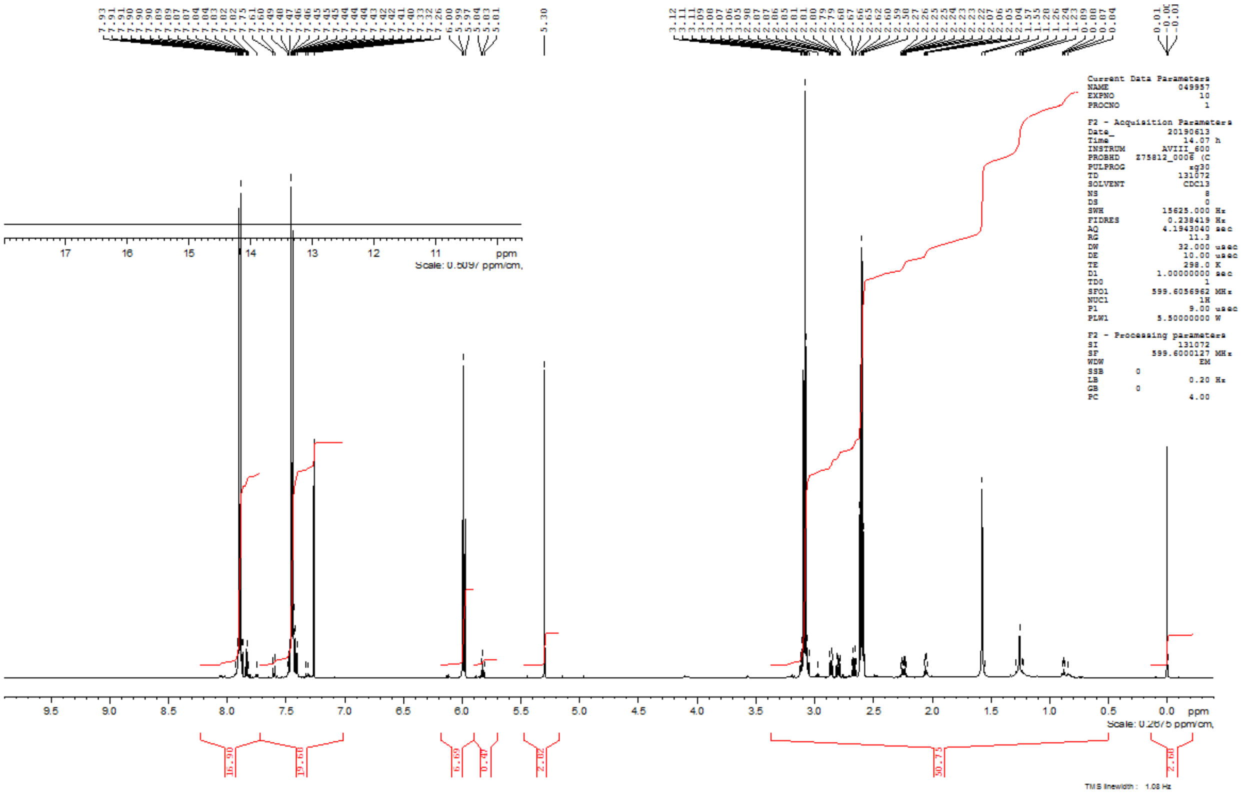

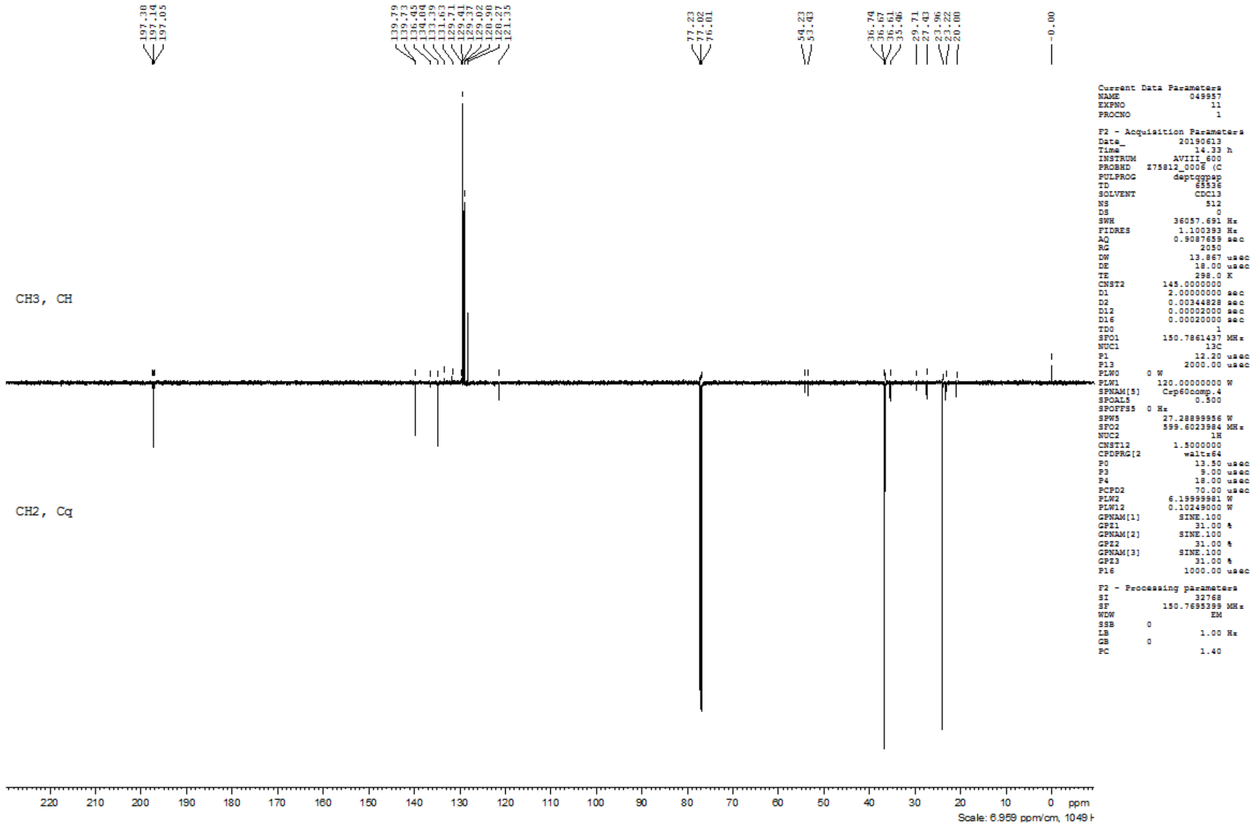

5,5-Dichloro-1-(2-chlorophenyl)pent-4-en-1-one (6e)

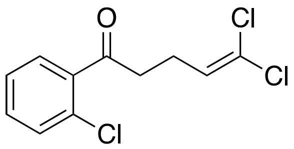

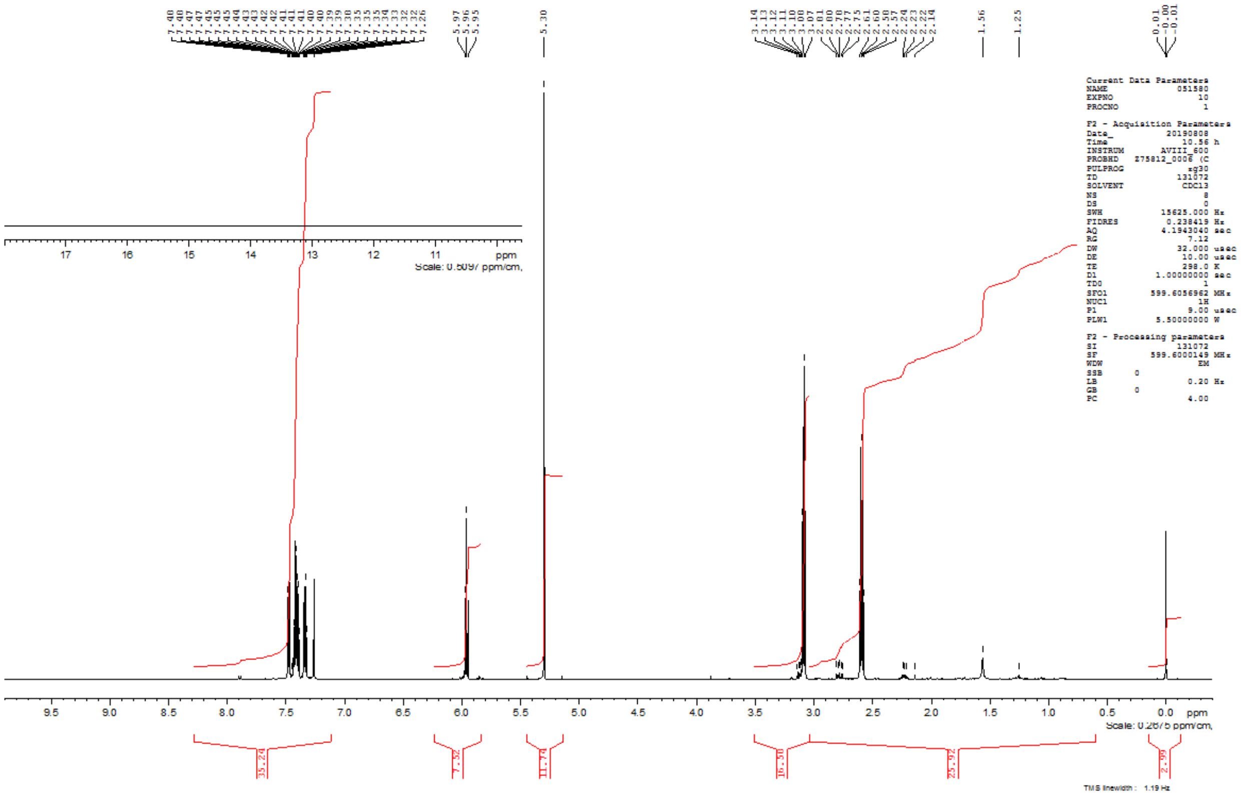

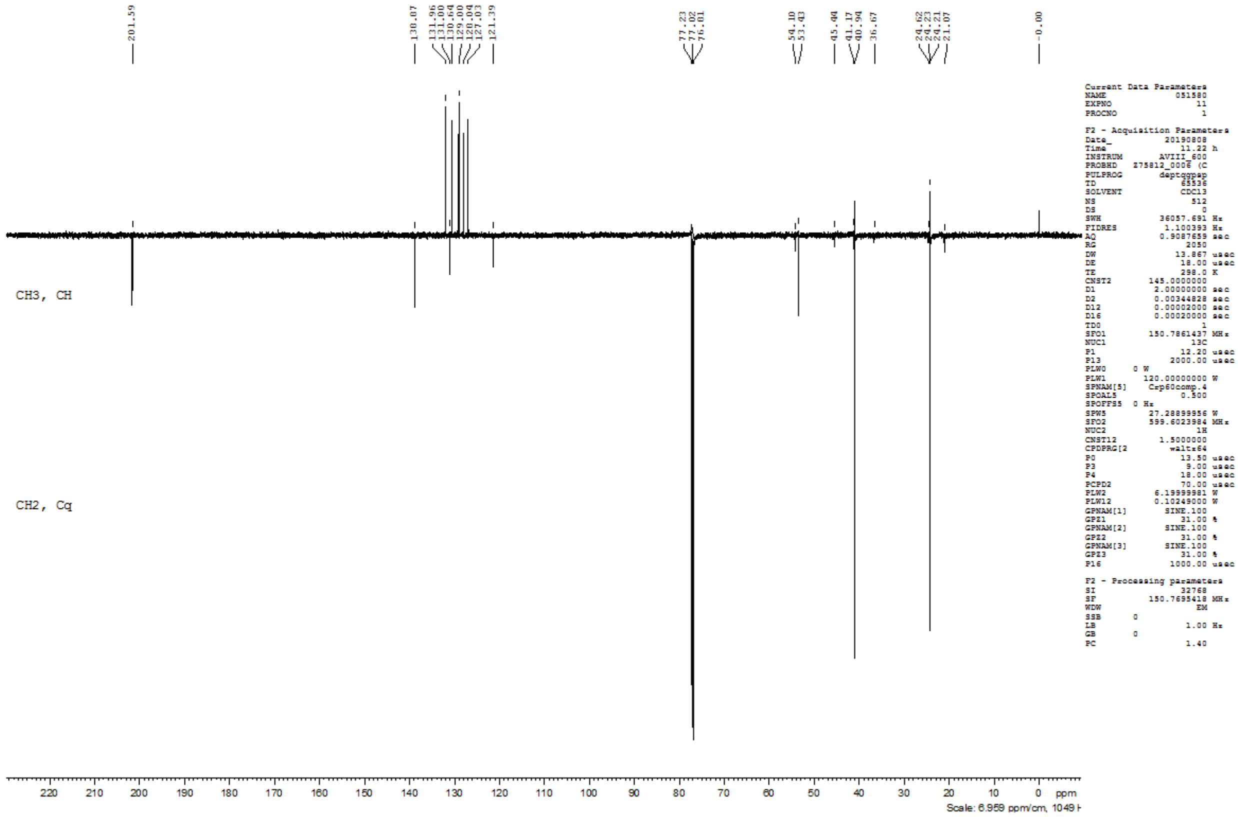

5,5-Dichloro-1-(*p*-tolyl)pent-4-en-1-one (6f)

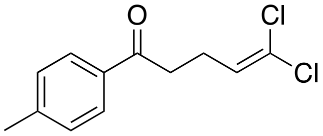

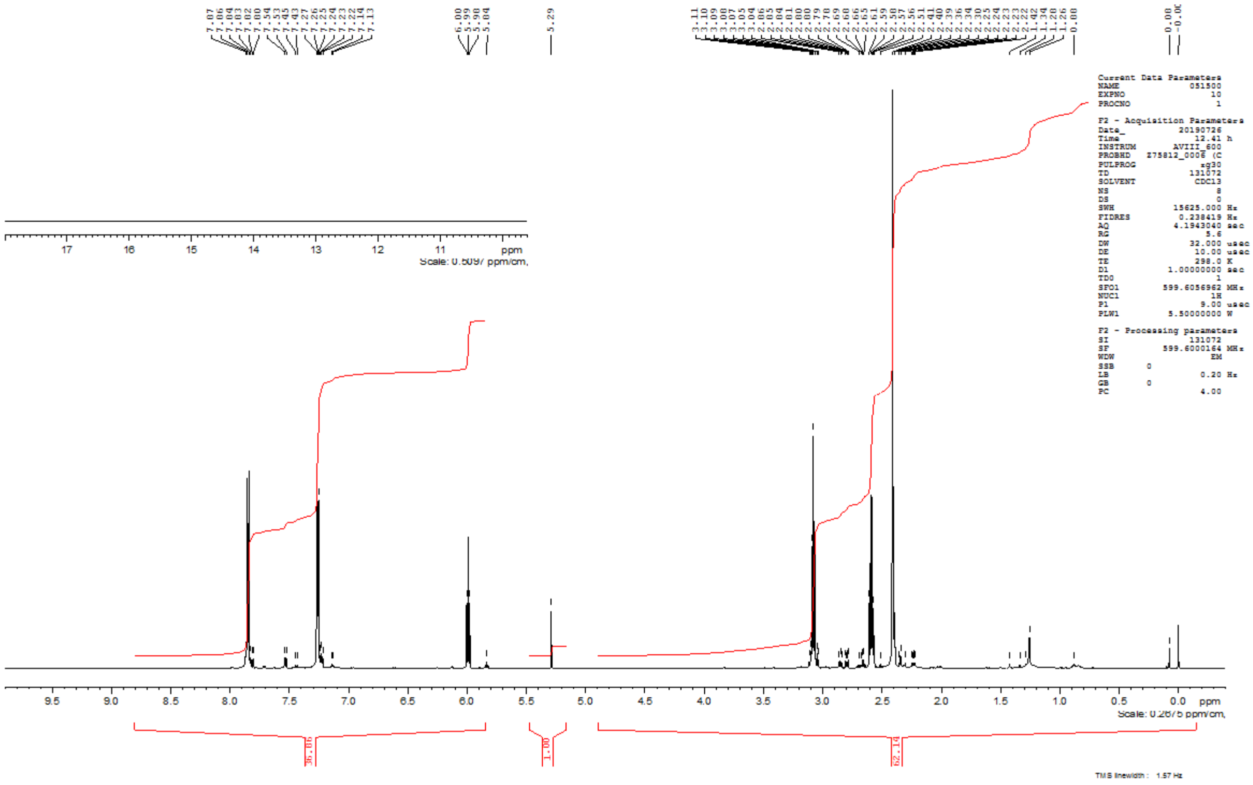

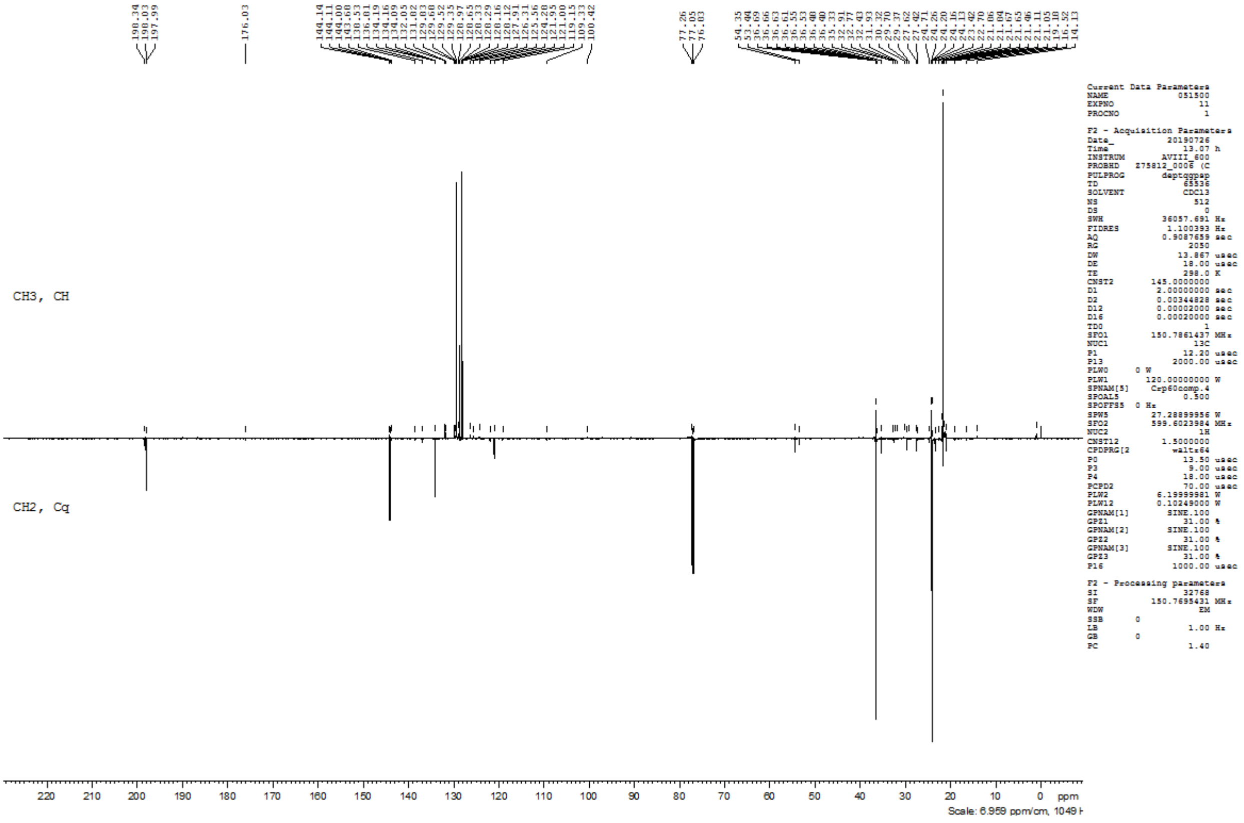

5,5-Dichloro-1-(4-bromophenyl)pent-4-en-1-one (6g)

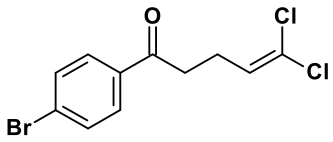

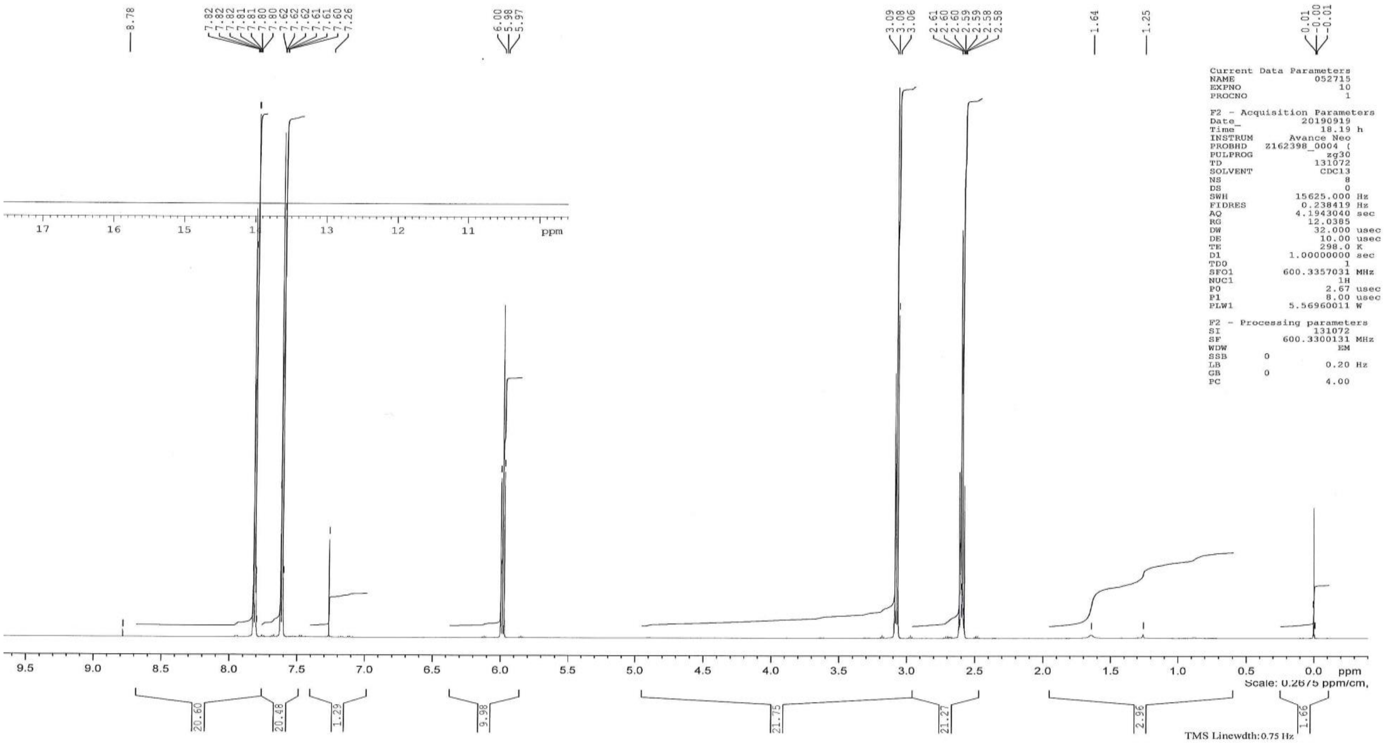

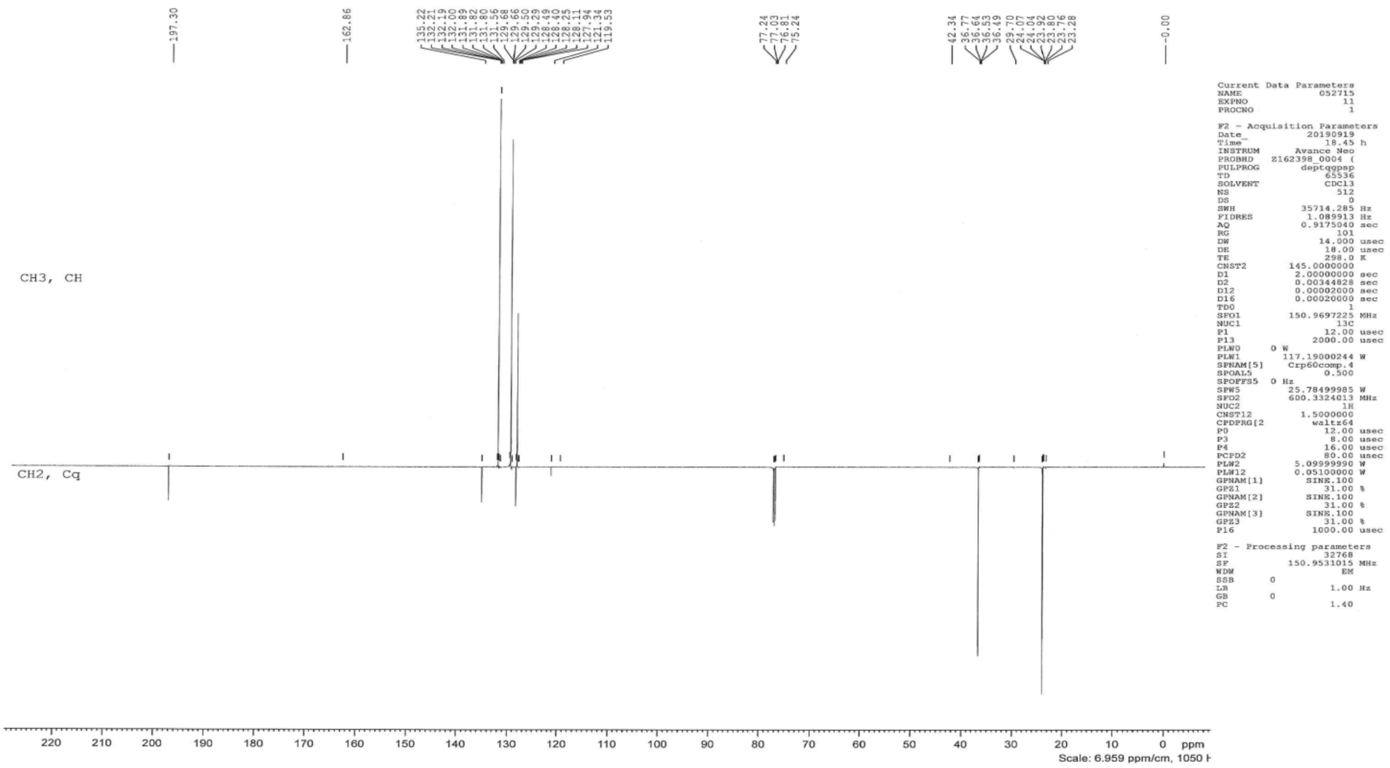

5,5-Dichloro-1-(2-naphthyl)pent-4-en-1-one (6h)

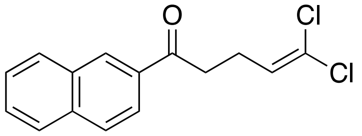

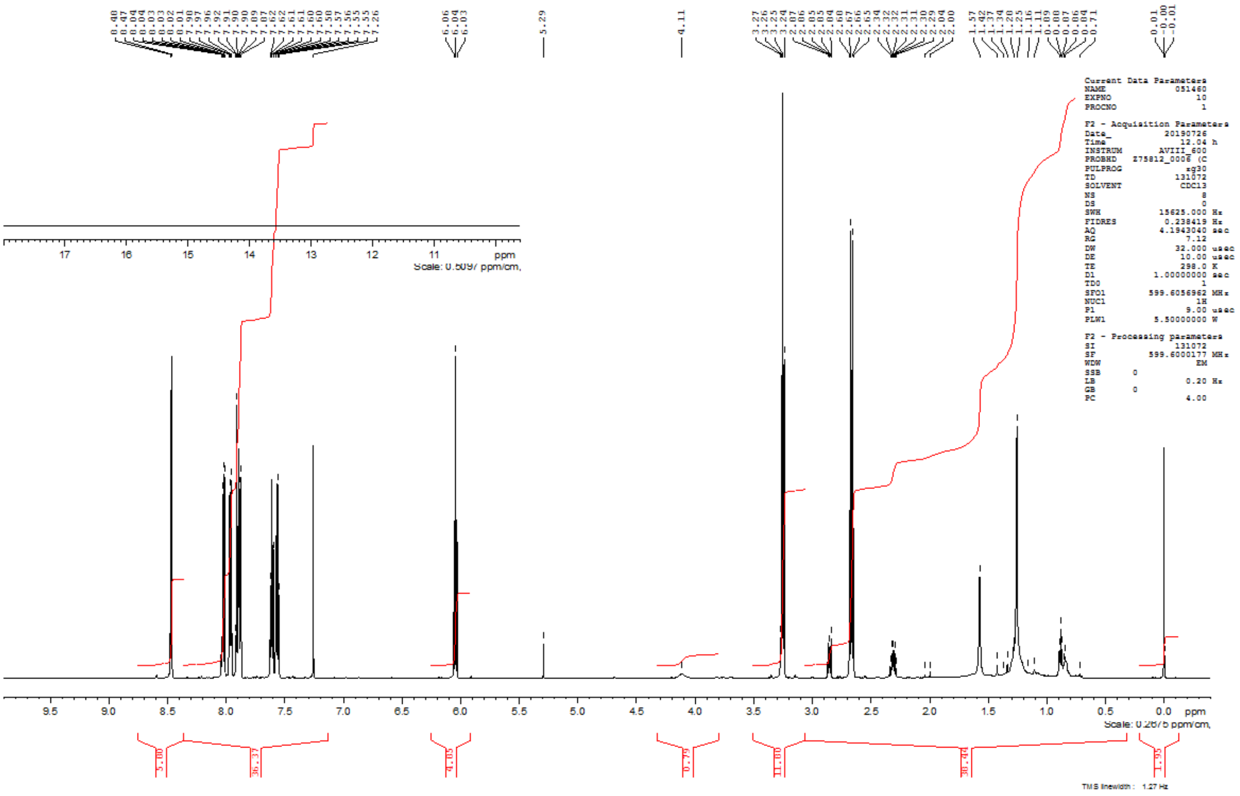

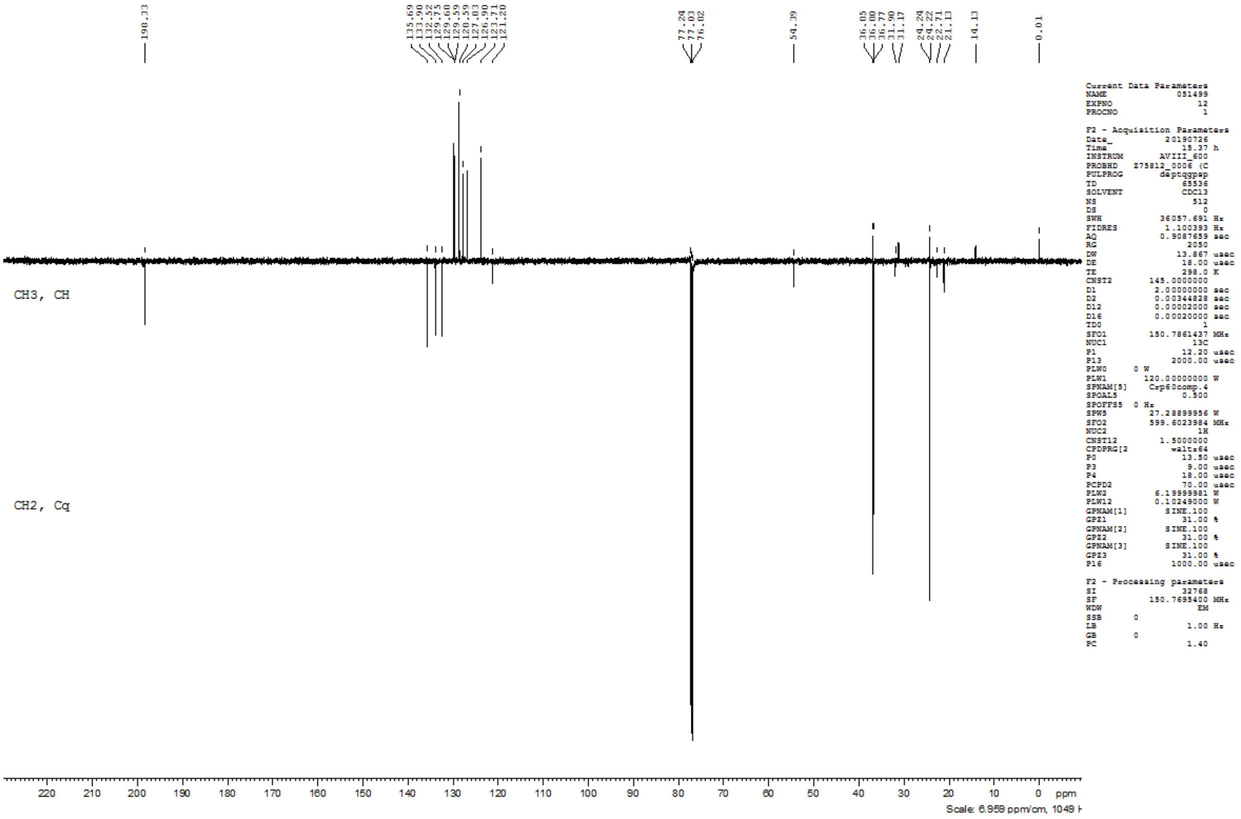

5,5-Dichloro-1-(2-pyridyl)pent-4-en-1-one (6i)

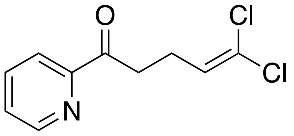

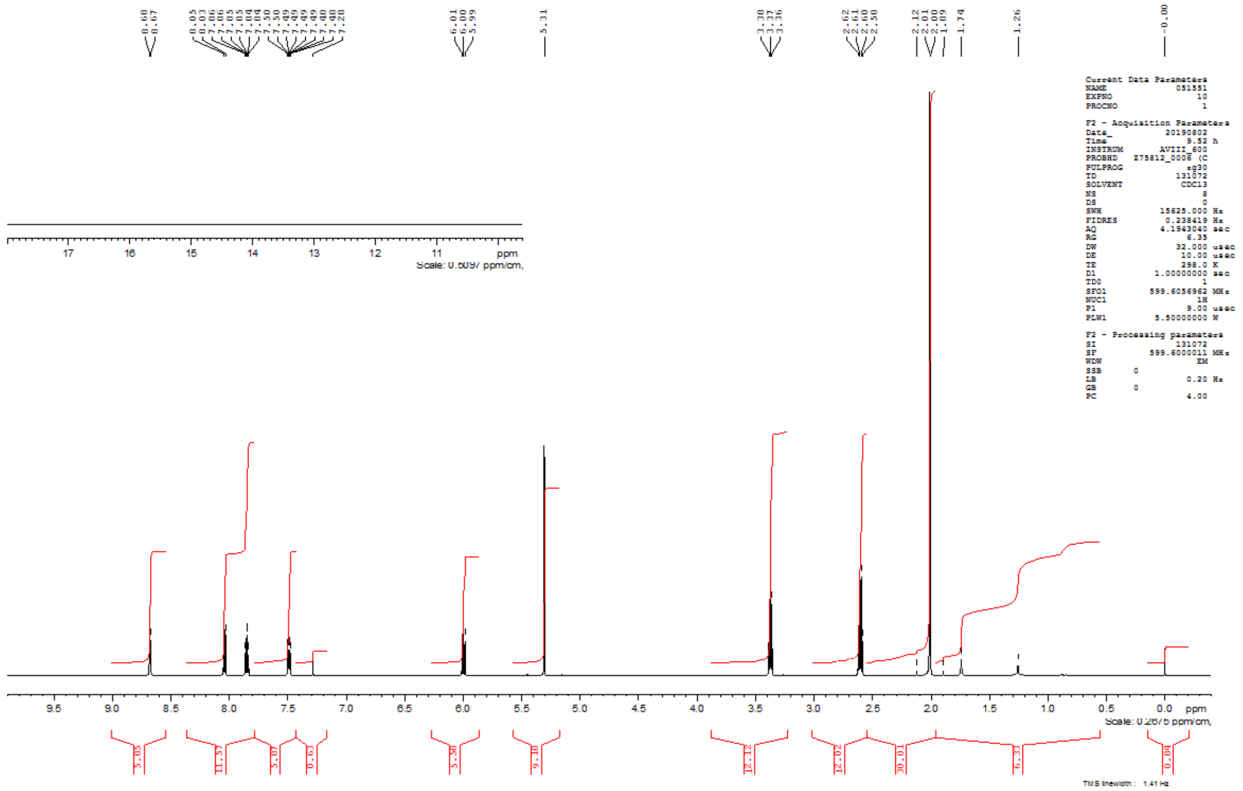

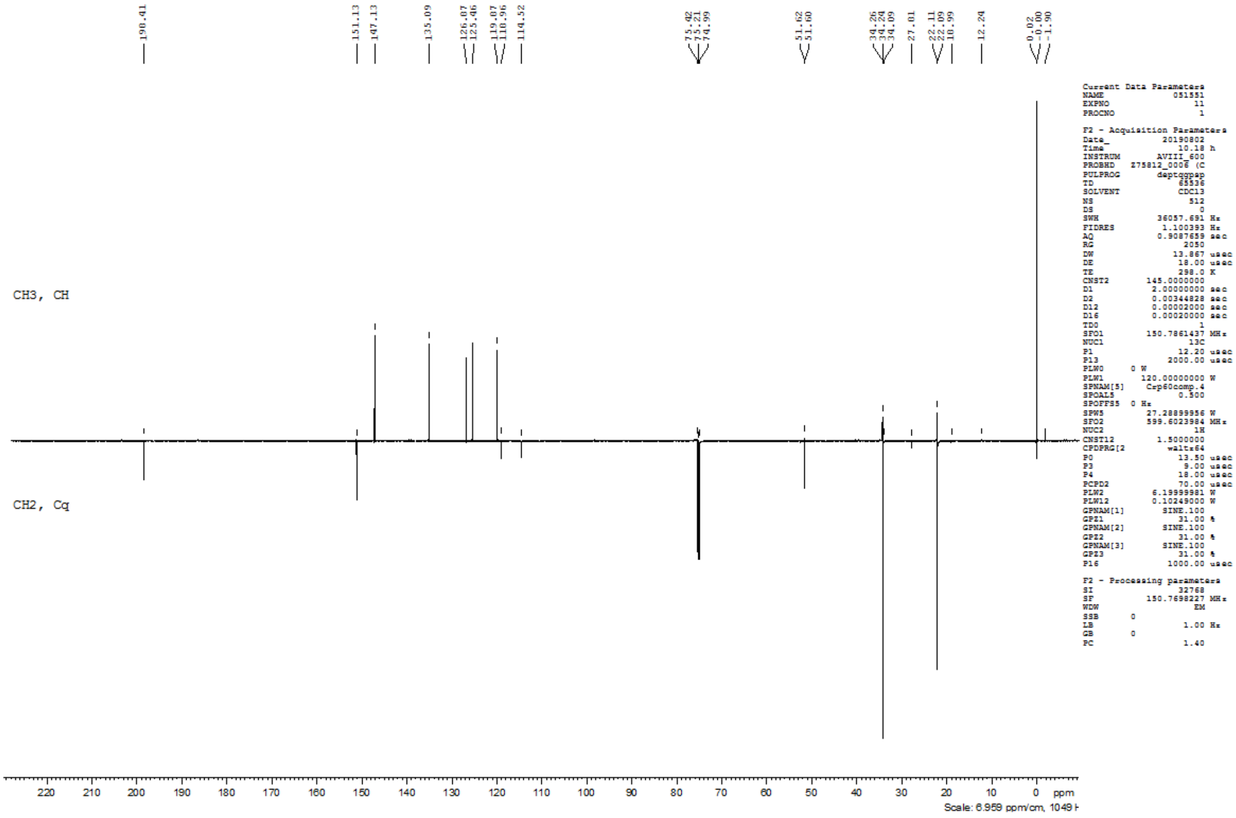

4,4-Dichloro-1-phenyl-but-3-en-1-one (6l)

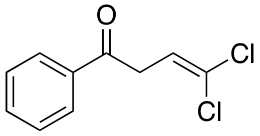

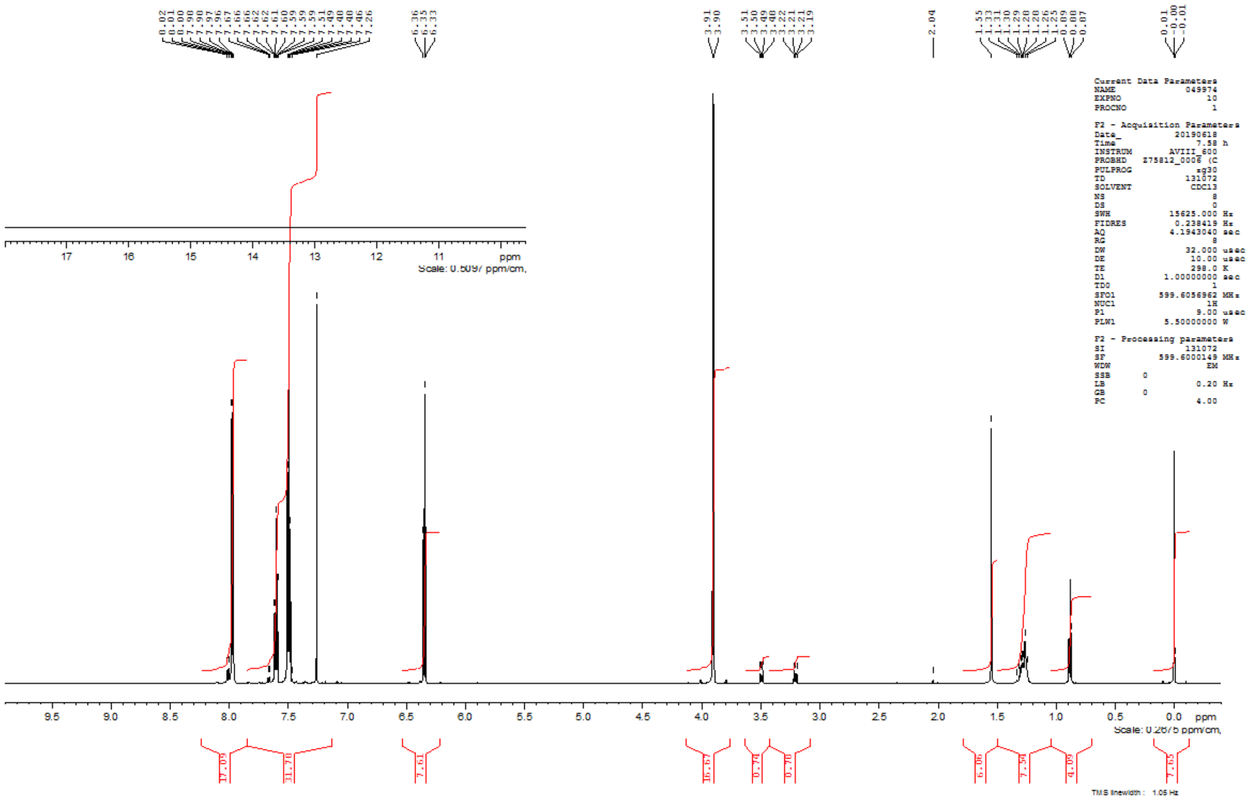

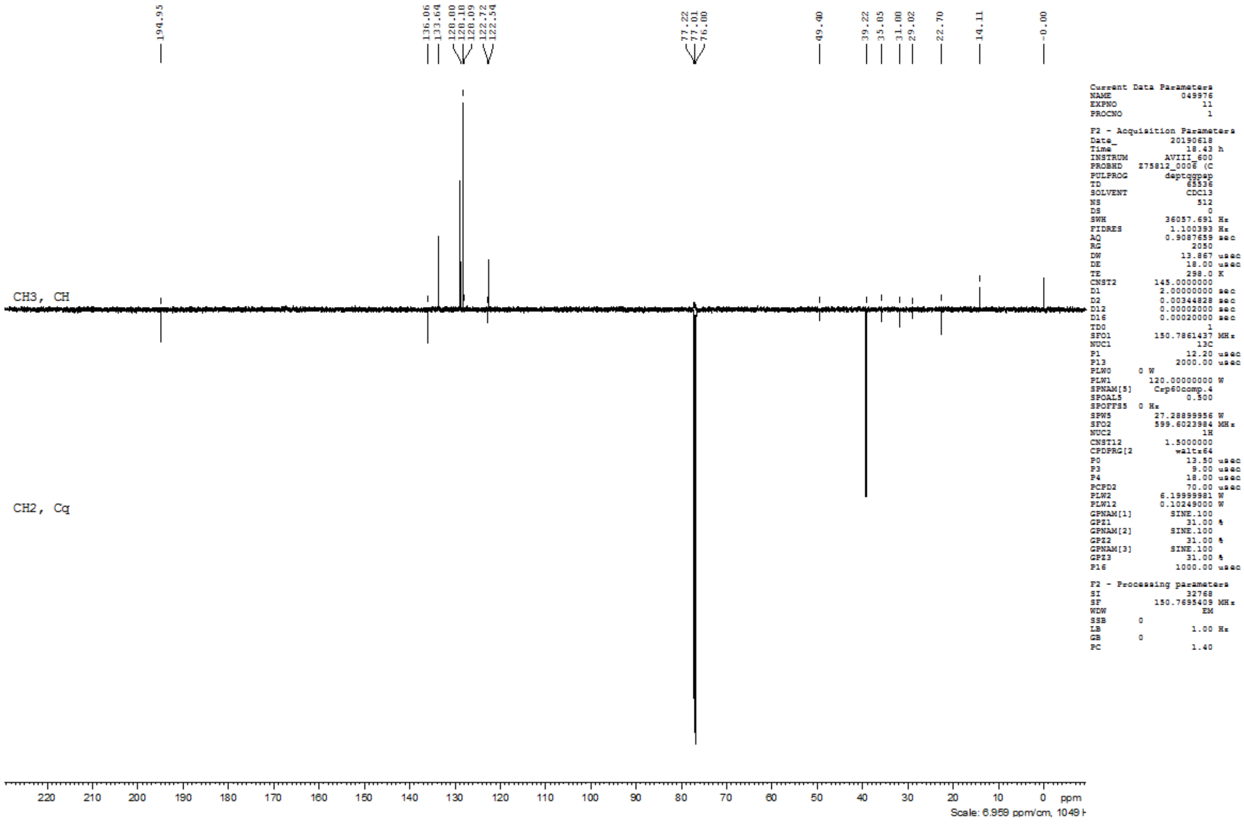

3,3-Dichloro-1-phenyl-prop-2-ene-1-one (6m)

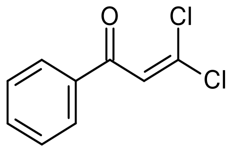

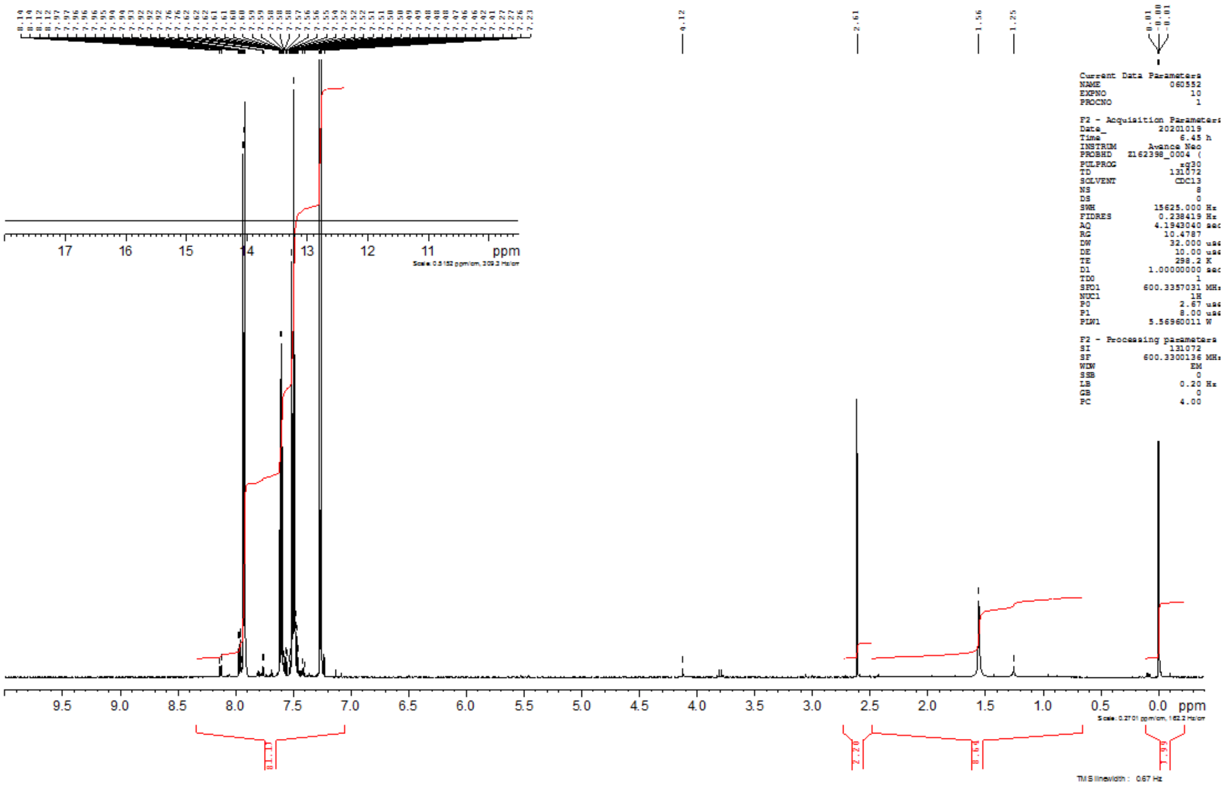

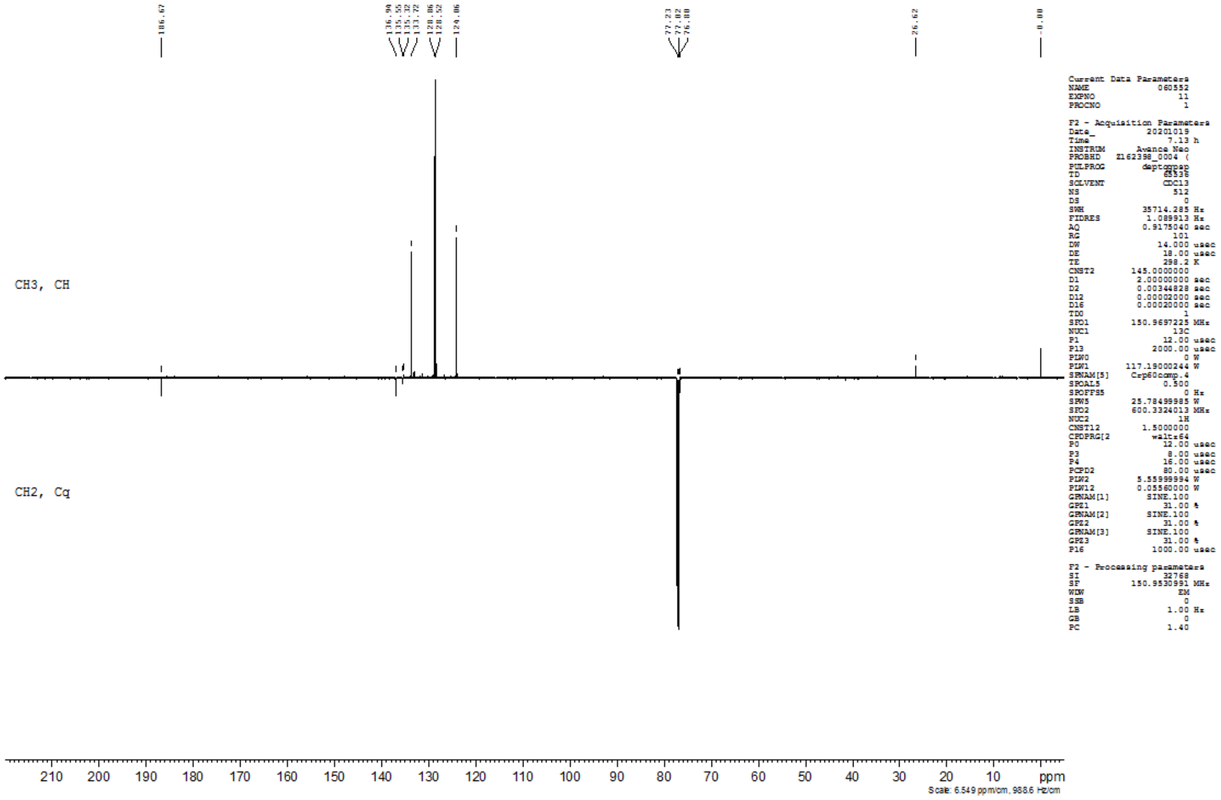

5-Chloro-4-methoxy-7,8-dihydrobenzo[7]annulen-9-one (9)

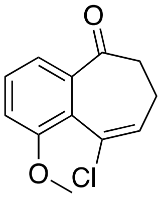

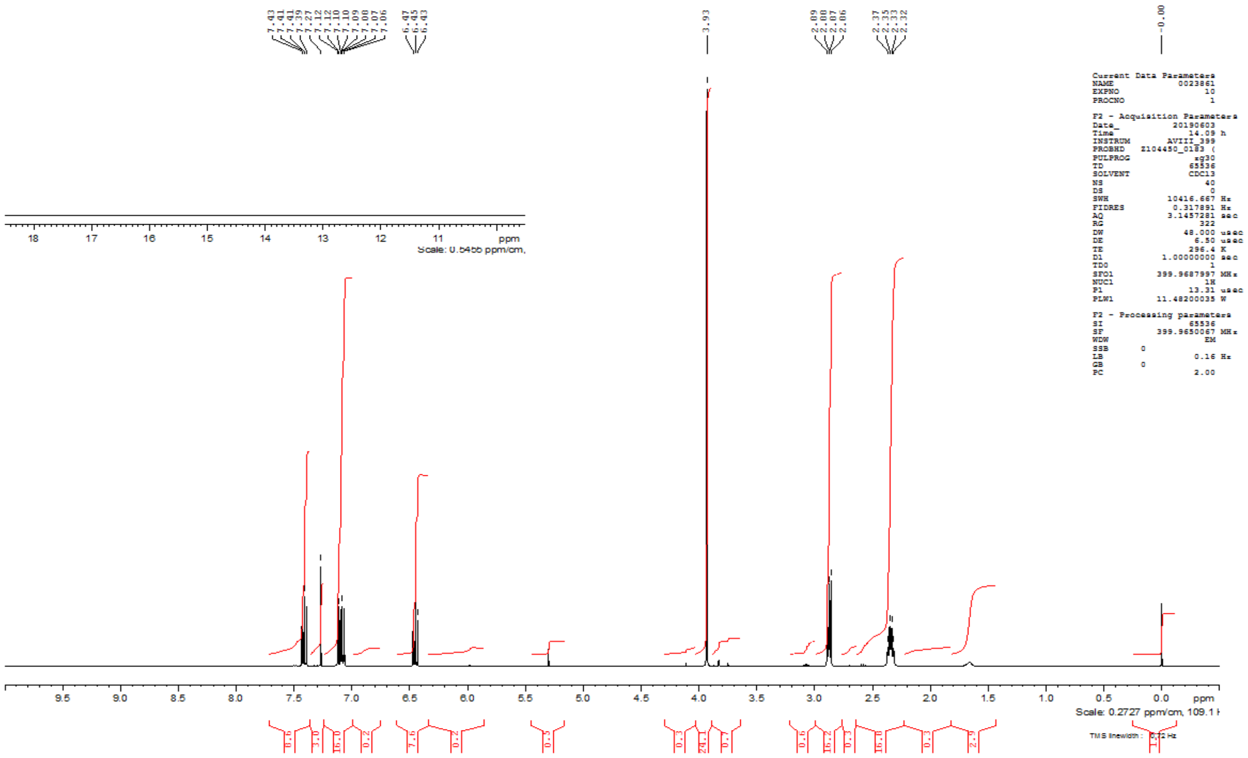

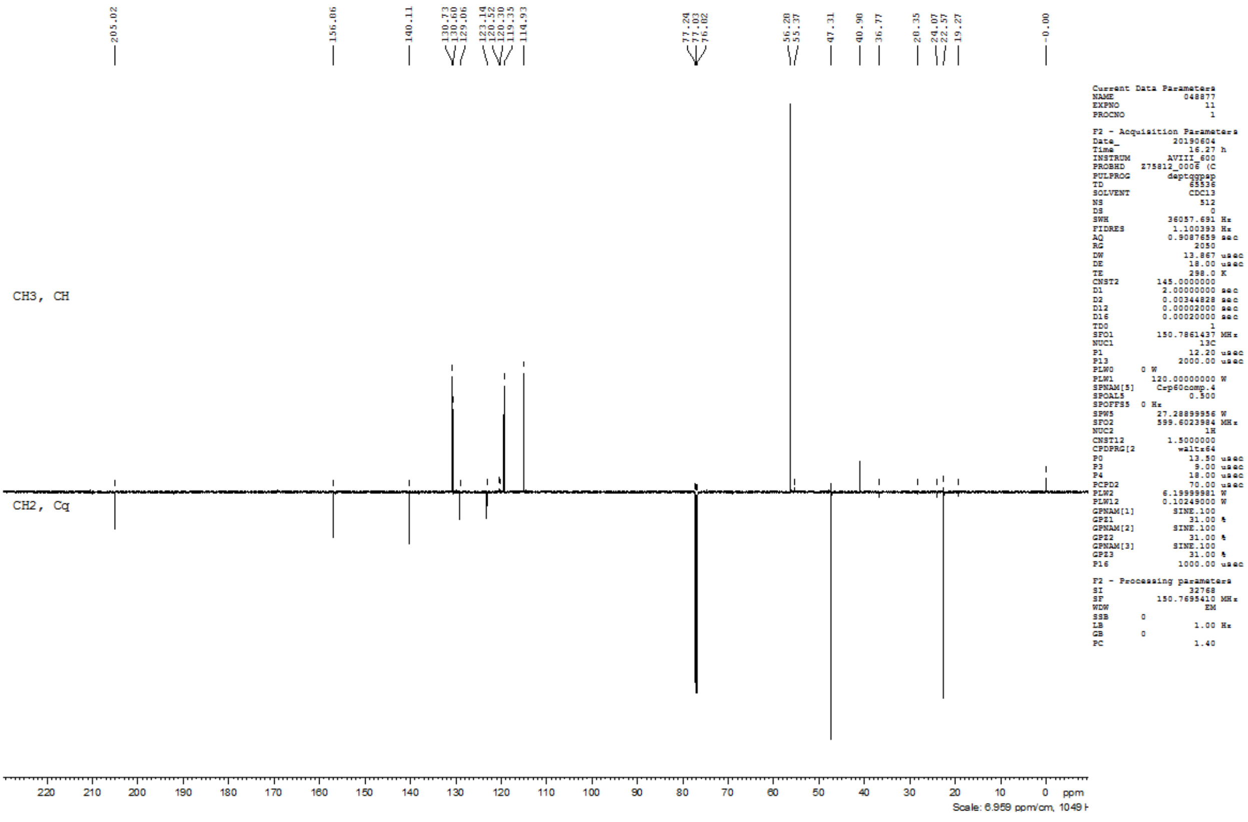

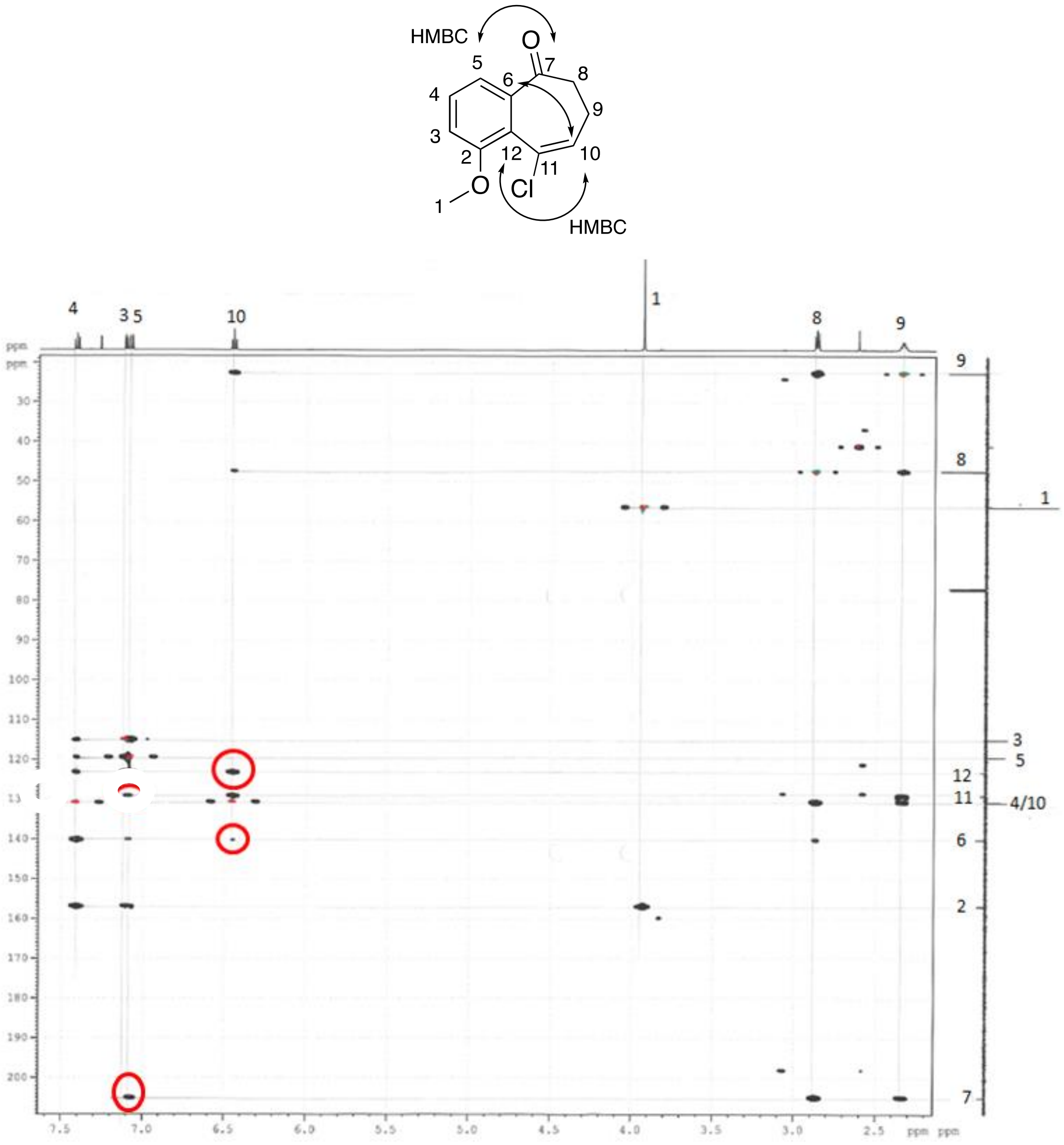

^1H-^{13}C-HSQCs (red and green) and ^1H-^{13}C-HMBCs (black) of 5-chloro-4-methoxy-7,8-dihydrobenzo[7]annulen-9-one (**9**).

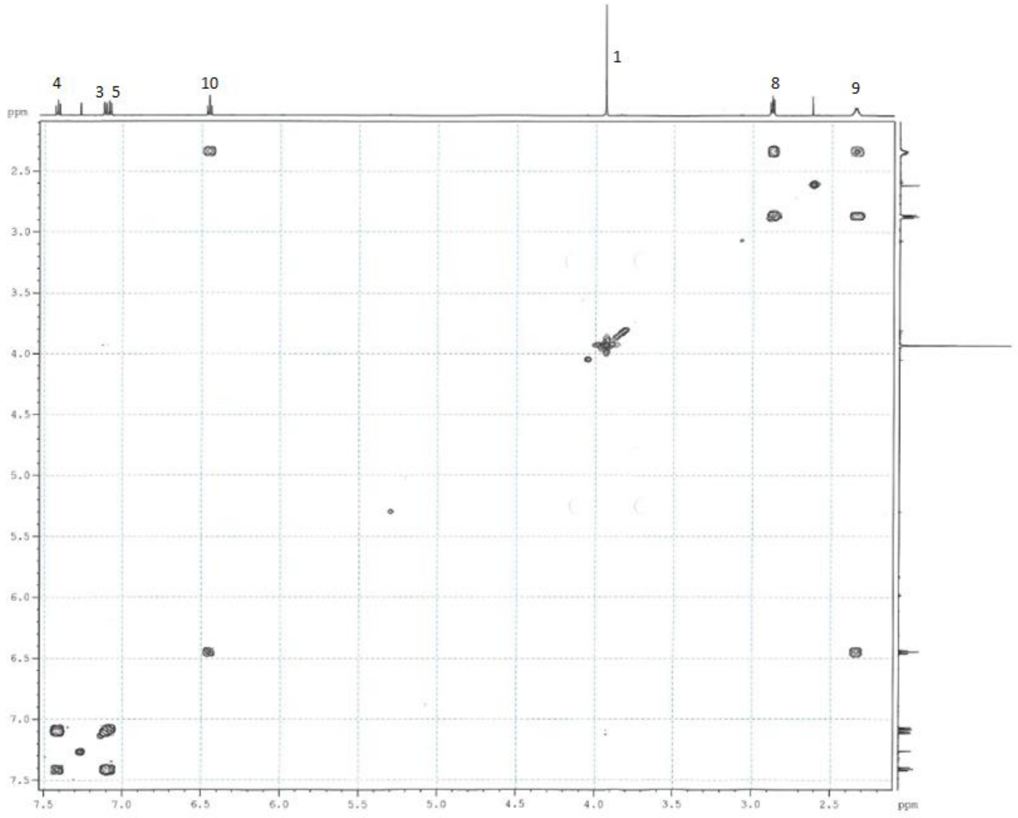

COSY of 5-chloro-4-methoxy-7,8-dihydrobenzo[7]annulen-9-one (**9**).

5-Chloro-2-methoxy-7,8-dihydrobenzo[7]annulen-9-one (10)

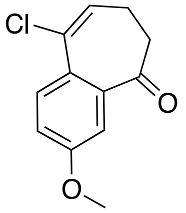

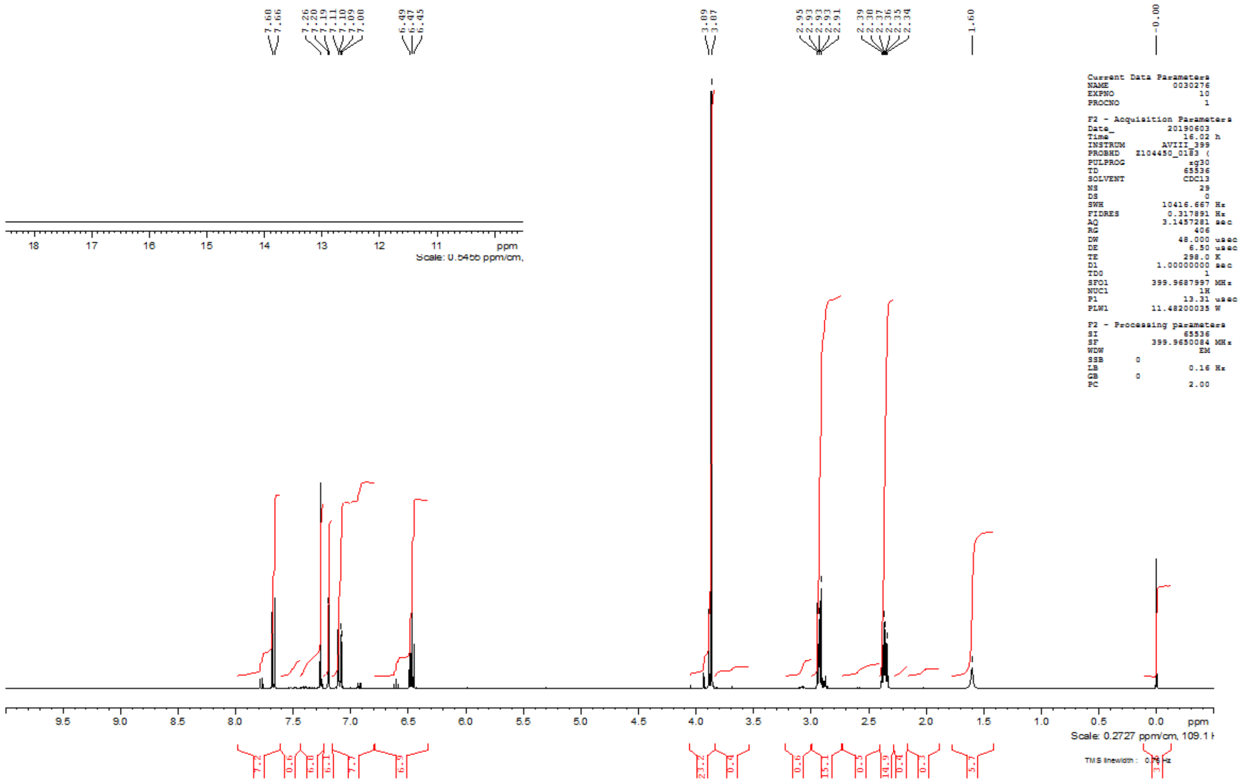

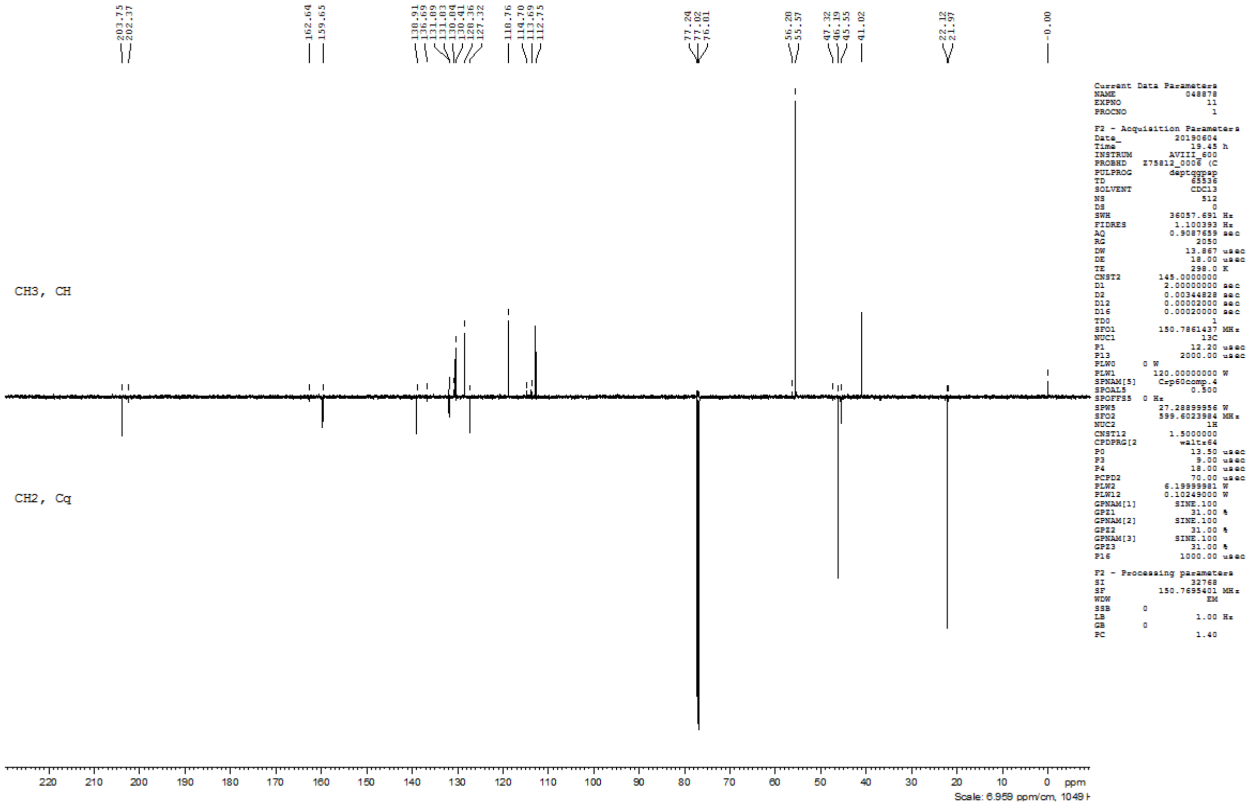

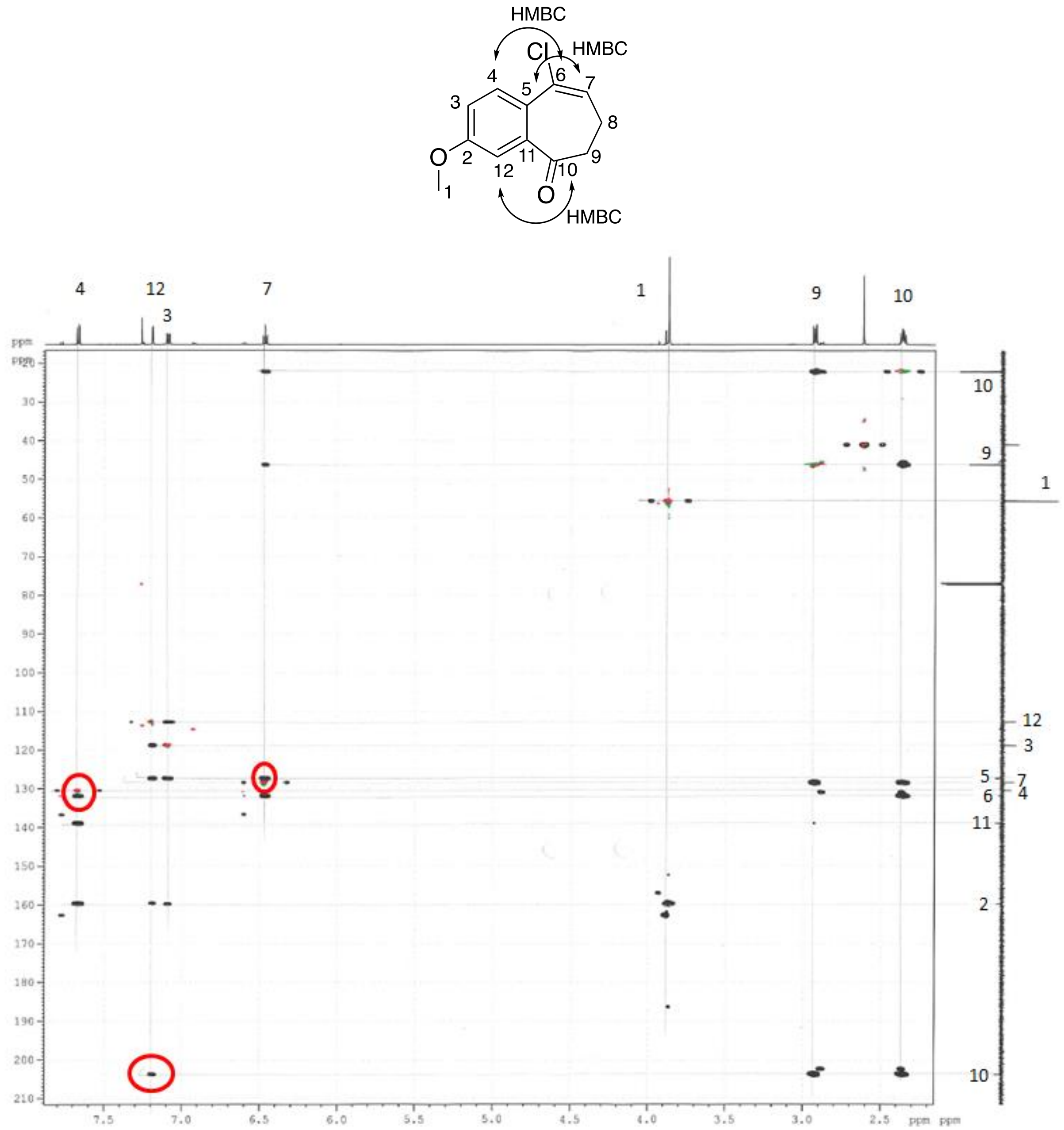

^{1}H-^{13}C-HSQCs (red and green) and ^{1}H-^{13}C-HMBCs (black) of 5-chloro-2-methoxy-7,8-dihydrobenzo[7]annulen-9-one (**10**).

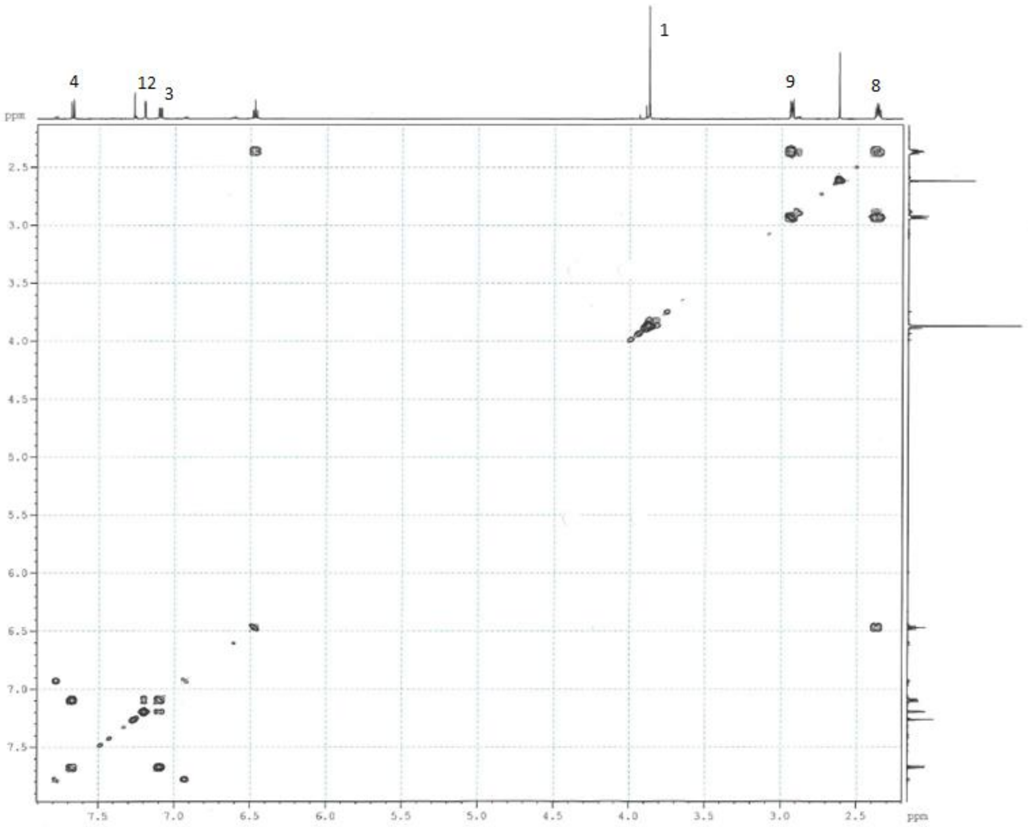

COSY of 5-chloro-2-methoxy-7,8-dihydrobenzo[7]annulen-9-one (**10**).

(*Z*)-5-Chloro-5-(4-methoxyphenyl)pent-4-enoyl chloride (13)

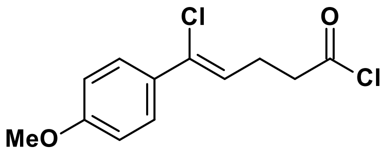

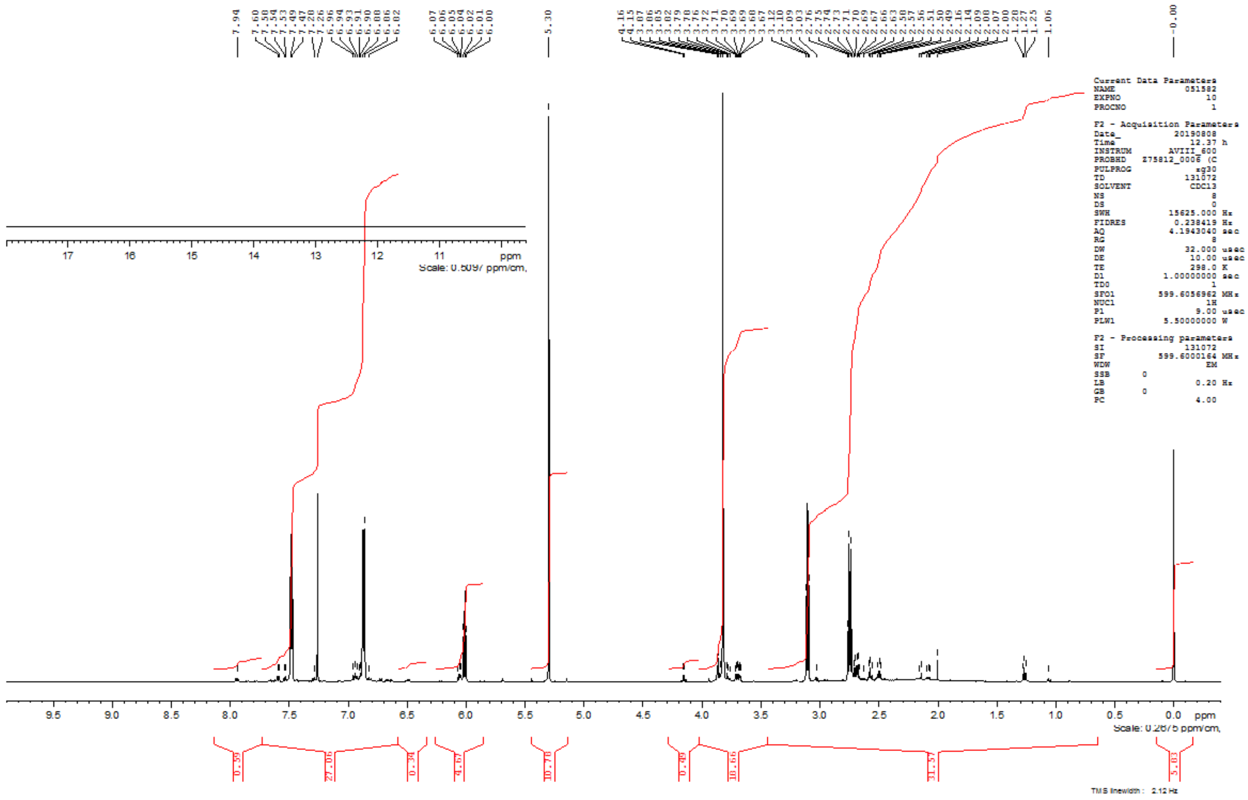

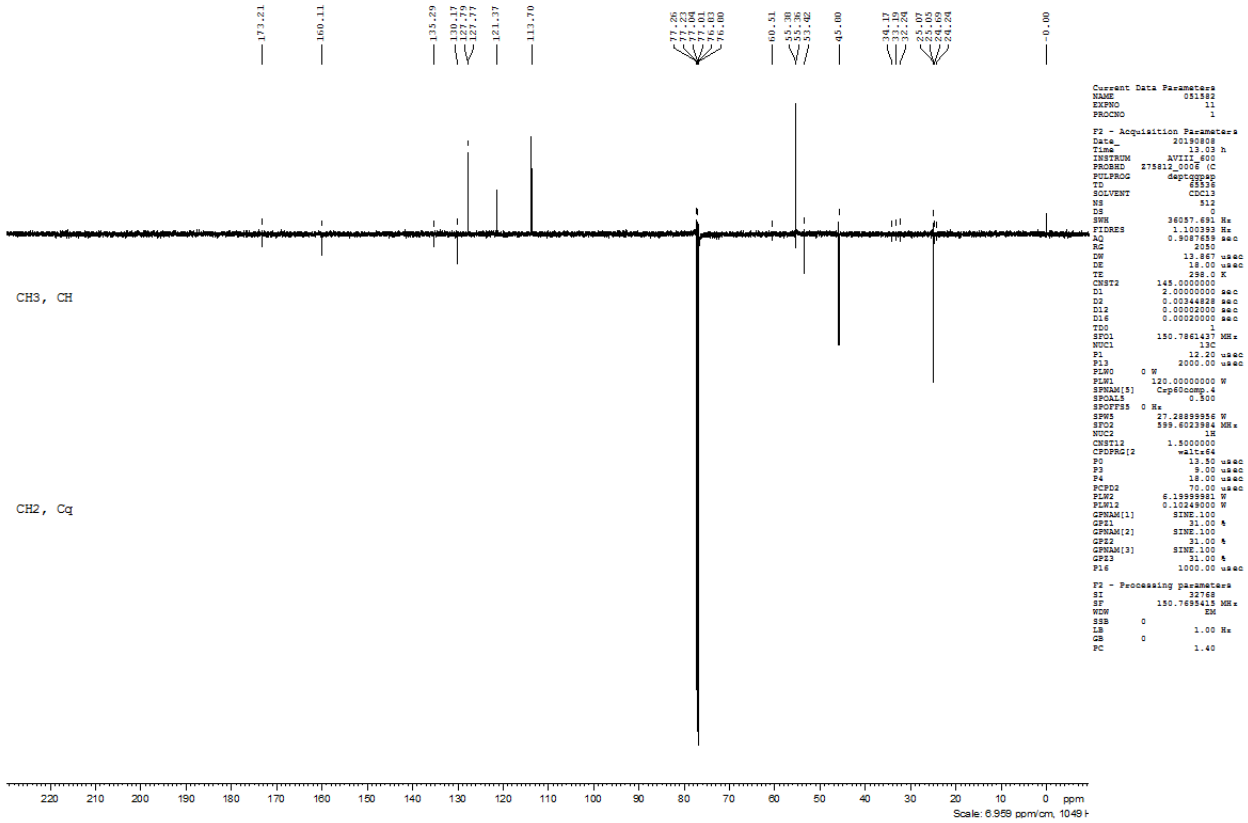

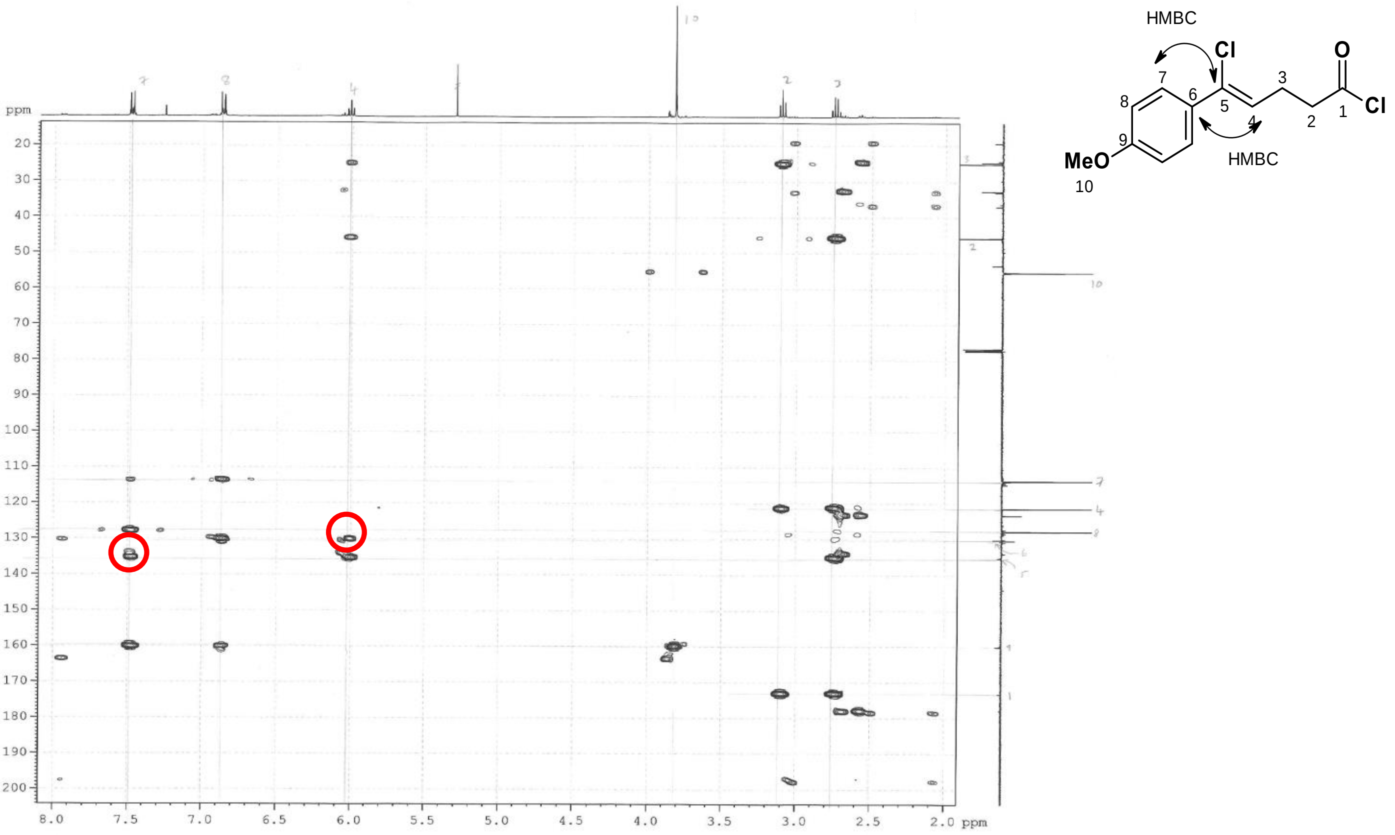

HMBC of **13**

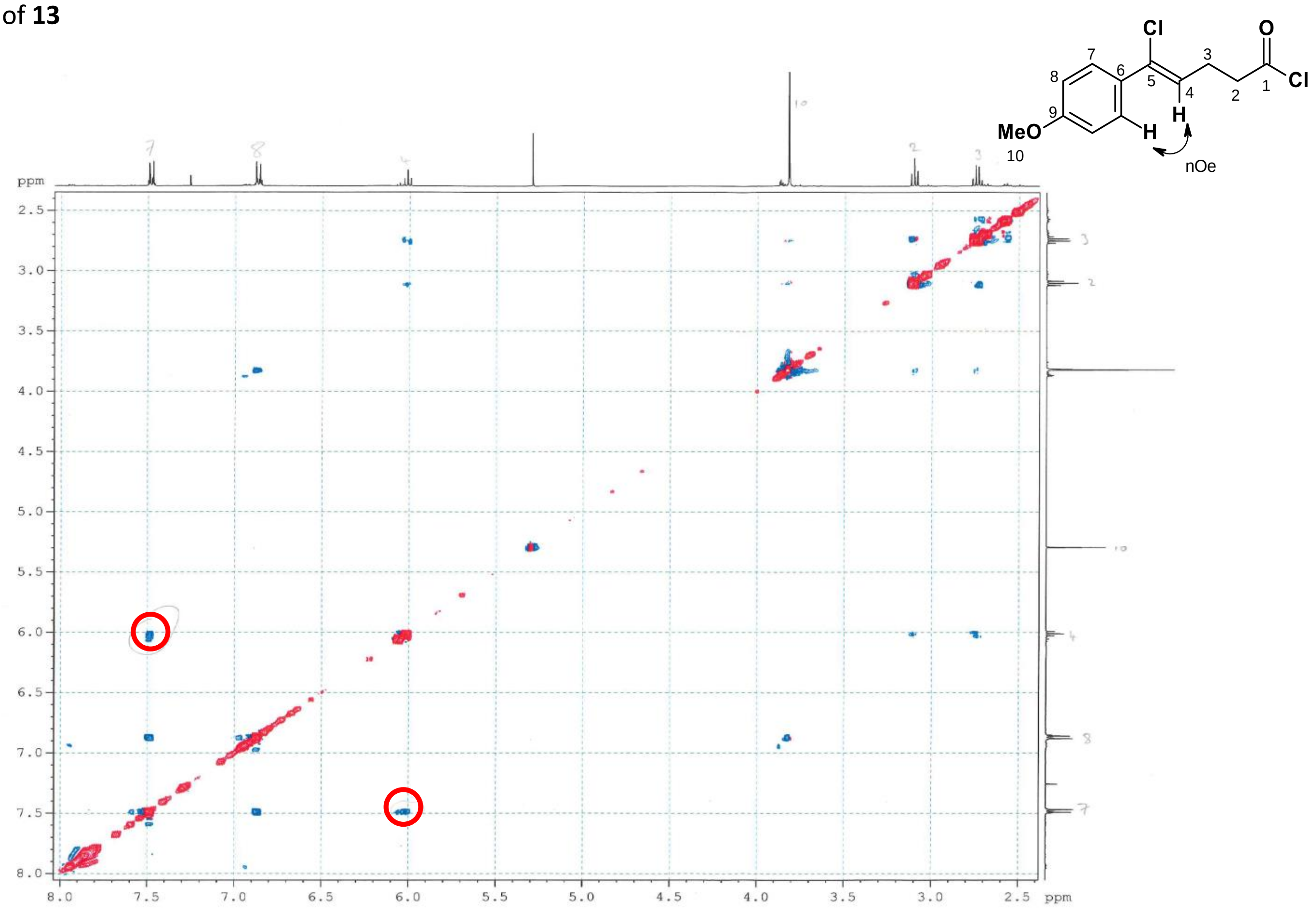

NOESY of **13**

(*Z*)-5-Chloro-5-(4-methoxyphenyl)pent-4-enoic acid (14)

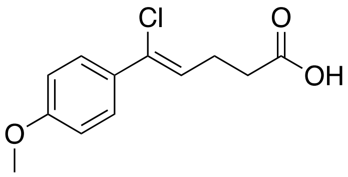

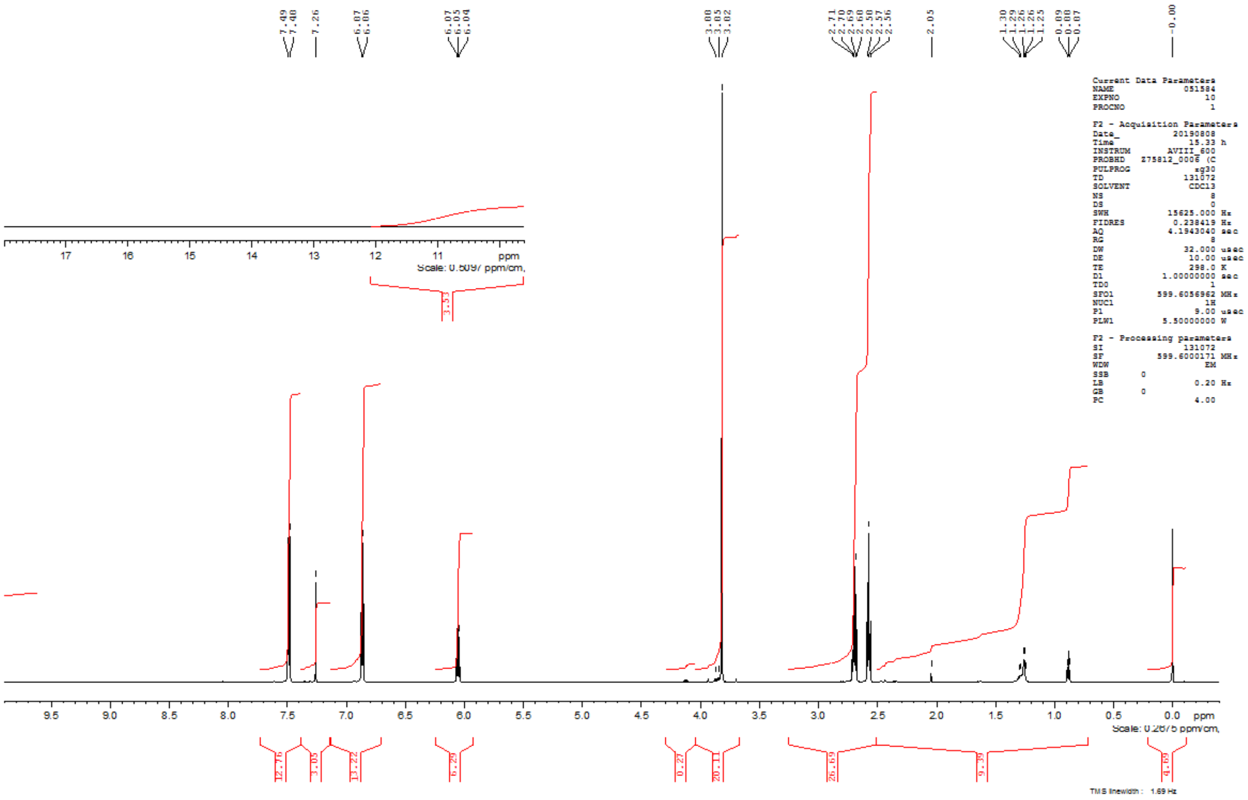

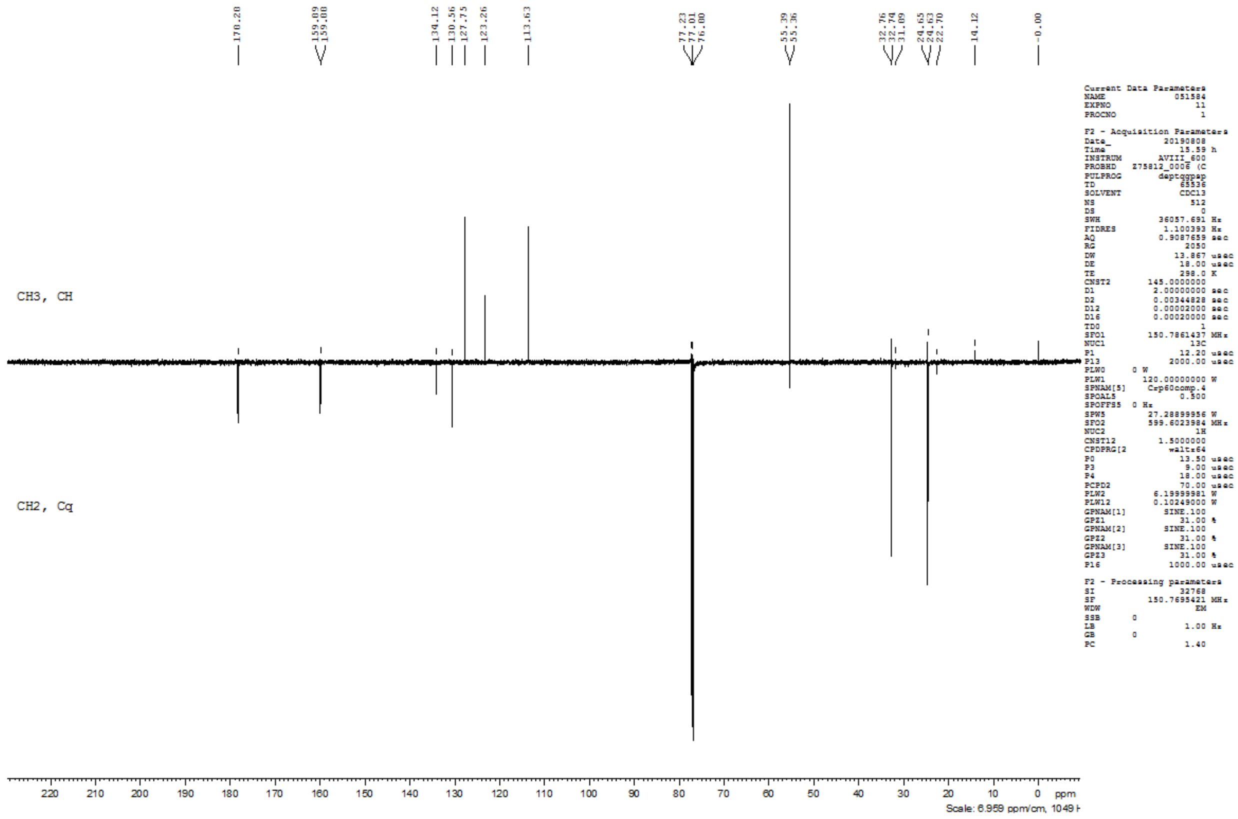

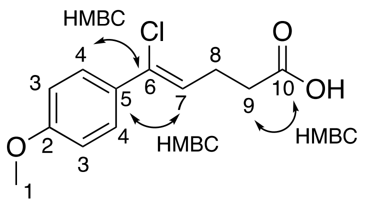

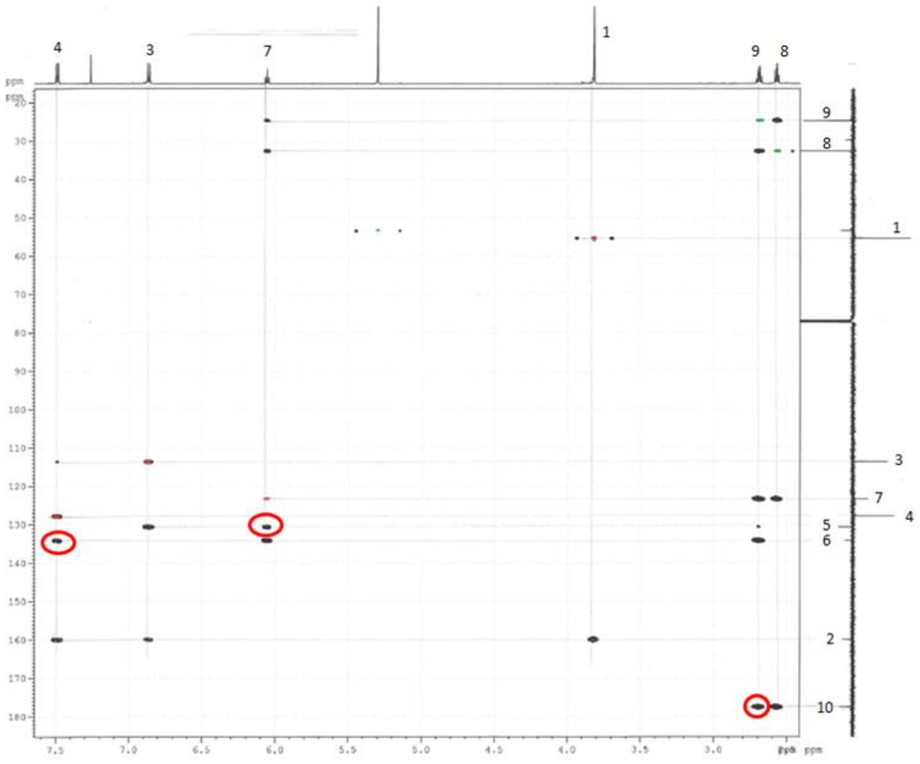

^{1}H-^{13}C-HSQCs (red and green) of ^{1}H-^{13}C-HMBCs (black) of (*Z*)-5-chloro-5-(4-methoxyphenyl)pent-4-enoic acid (**14**).

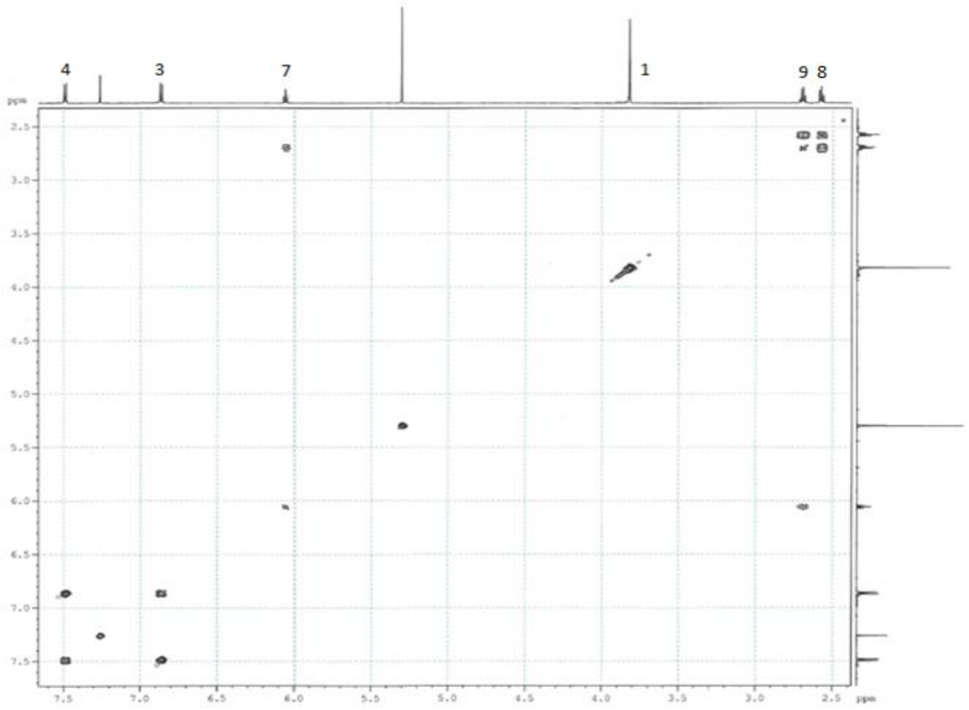

COSY of (*Z*)-5-chloro-5-(4-methoxyphenyl)pent-4-enoic acid (**14**).

5-(4-Methoxyphenyl)-5-oxo-pentanoic acid (15)

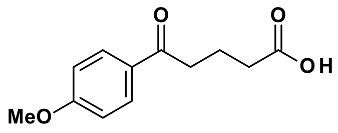

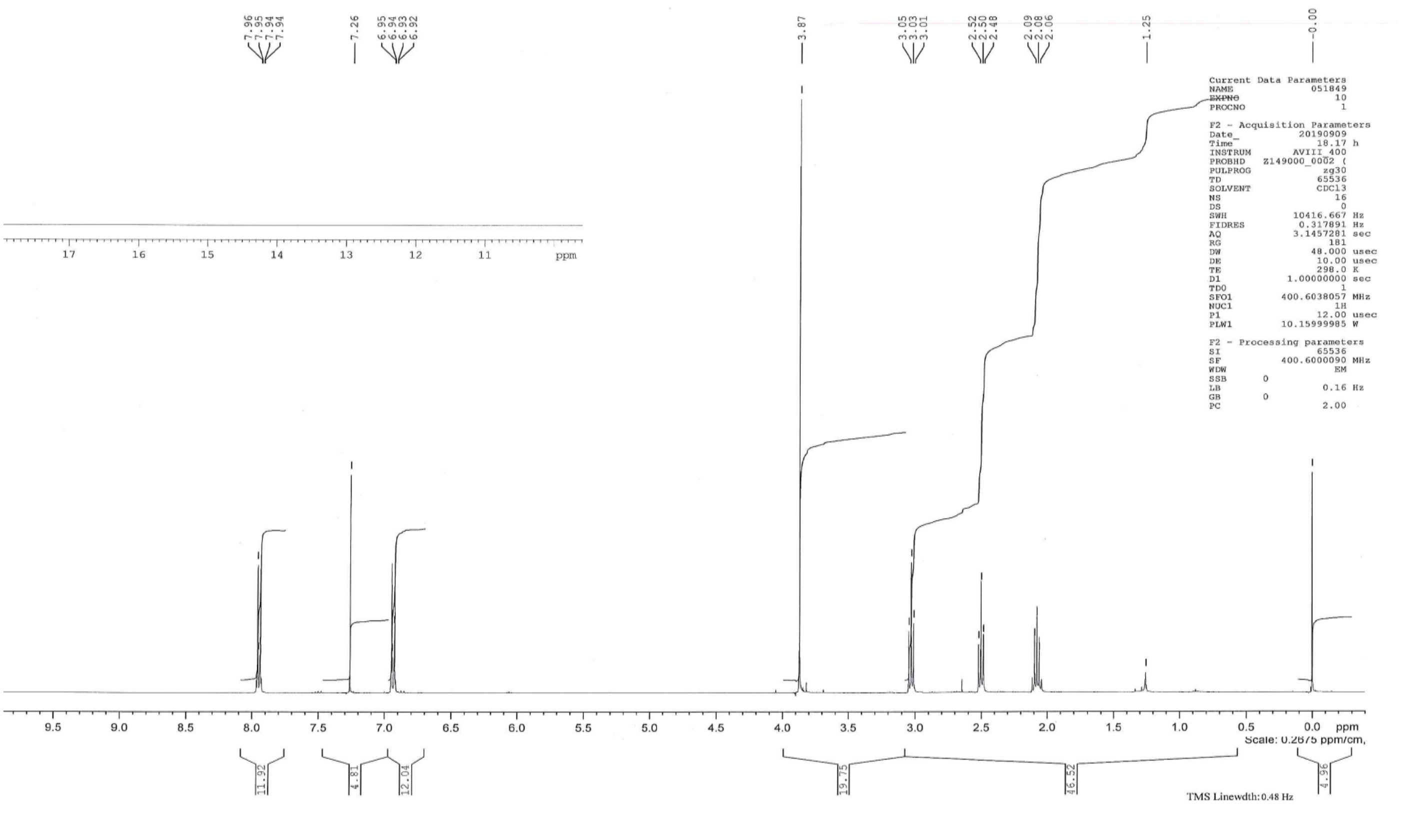

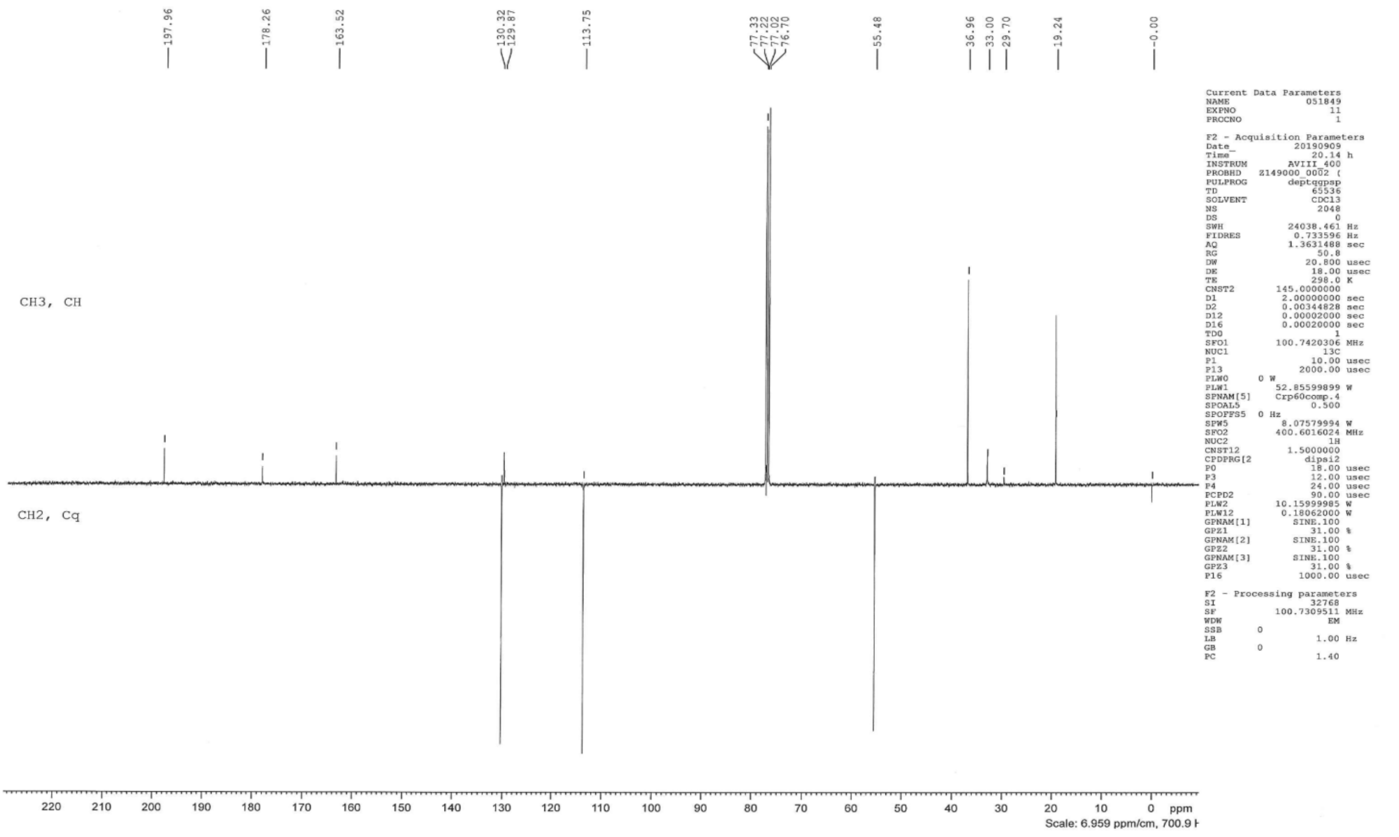

1-(4-Chlorophenyl)-5,5,5-trifluoro-pentan-1-ol (S-1)

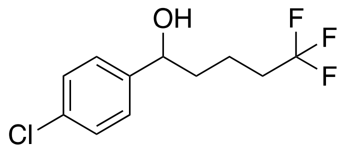

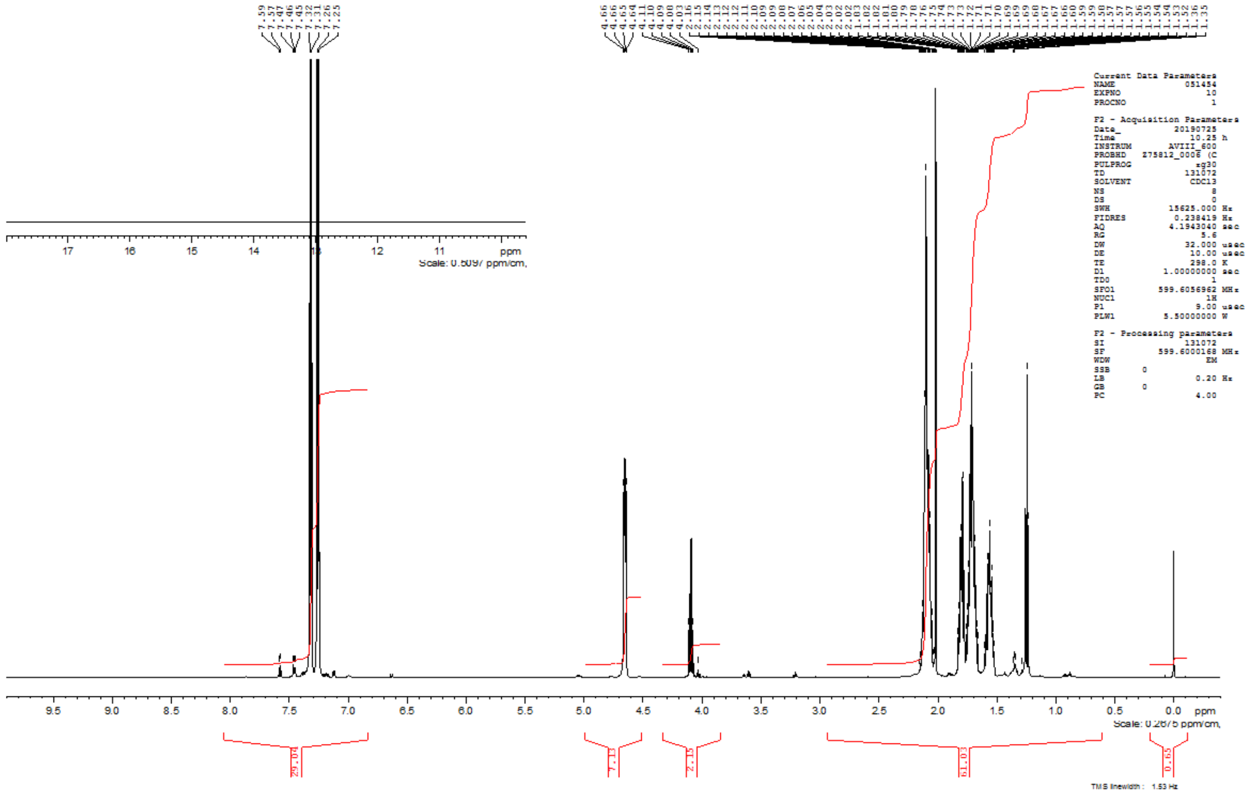

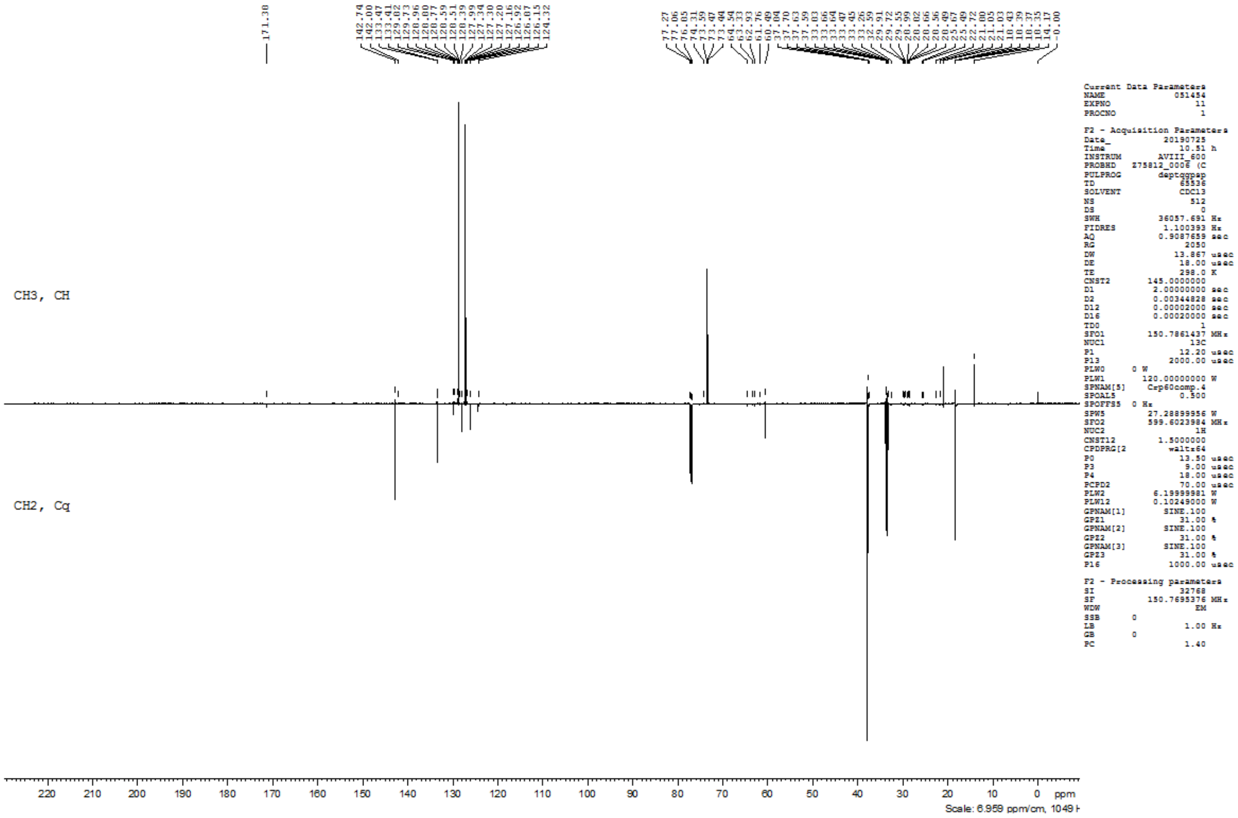

4,4,4-Trifluorobutylsulfanylbenzene (S-2)

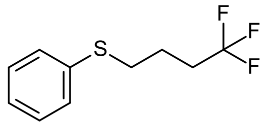

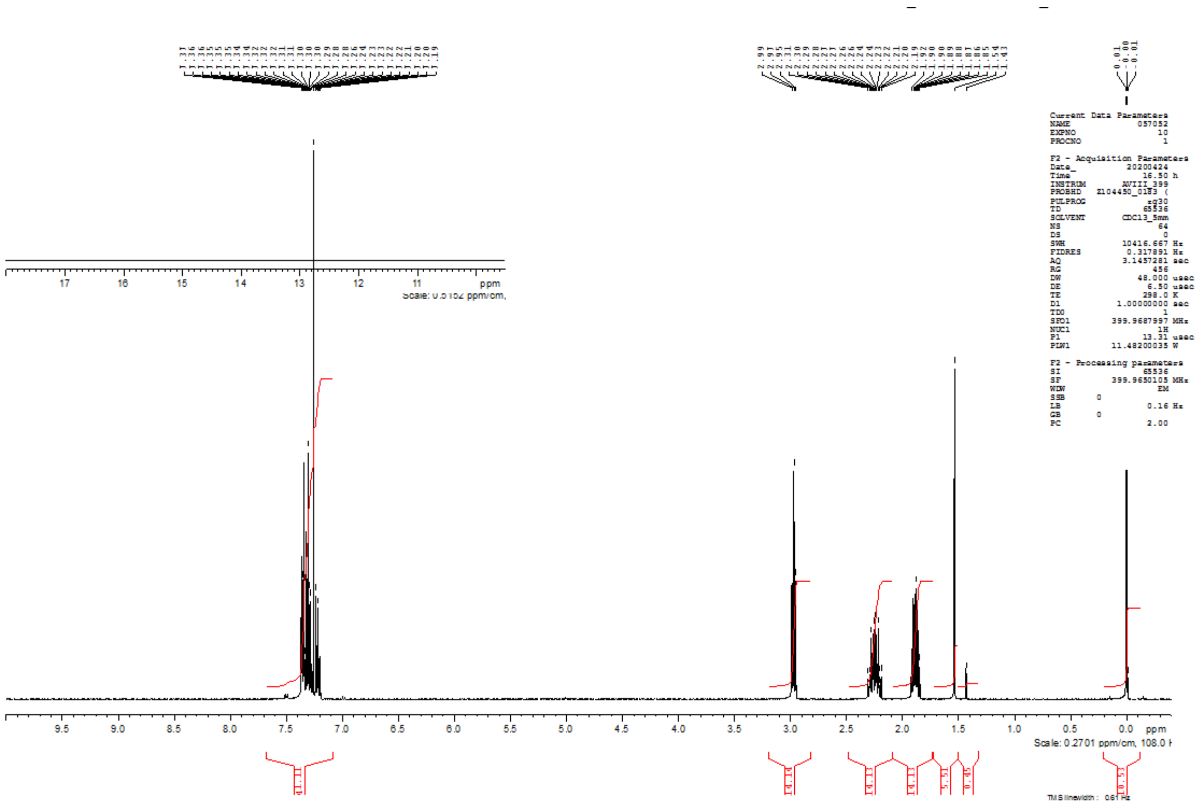

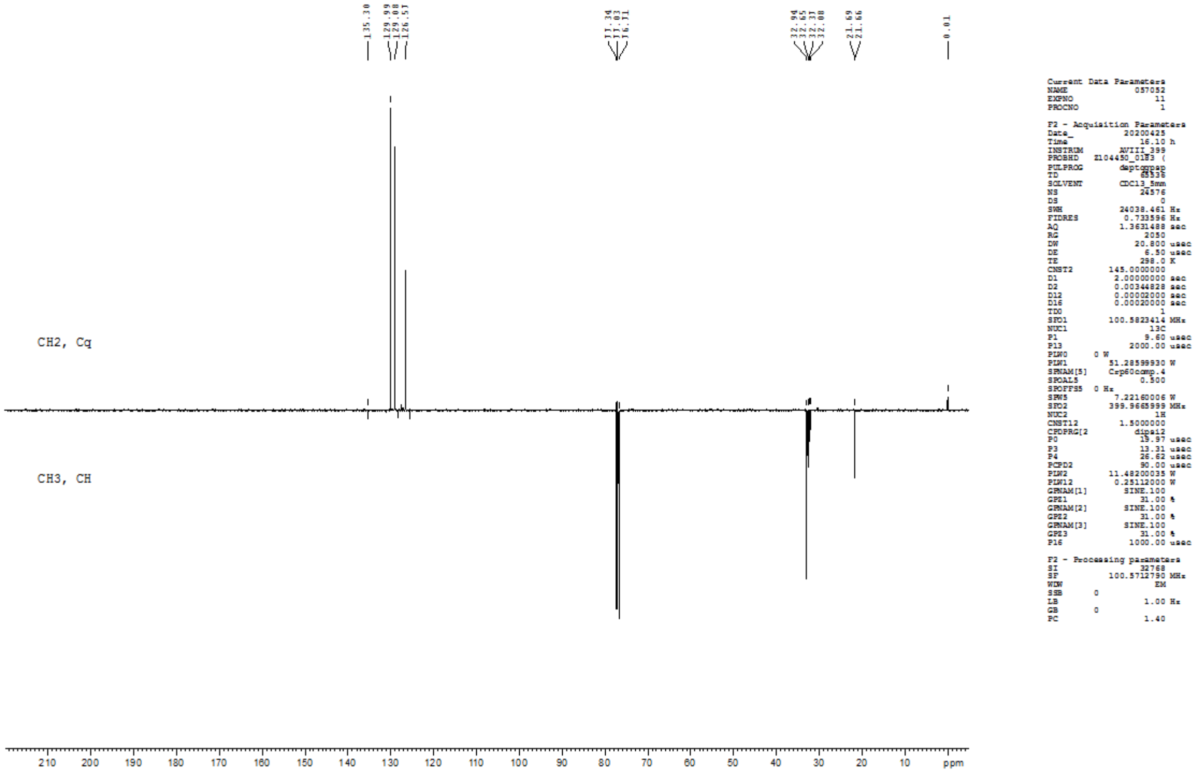

1-Methoxy-4-(4,4,4-trifluorobutylsulfanyl)benzene (S-3)

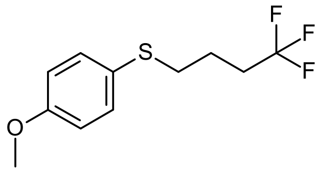

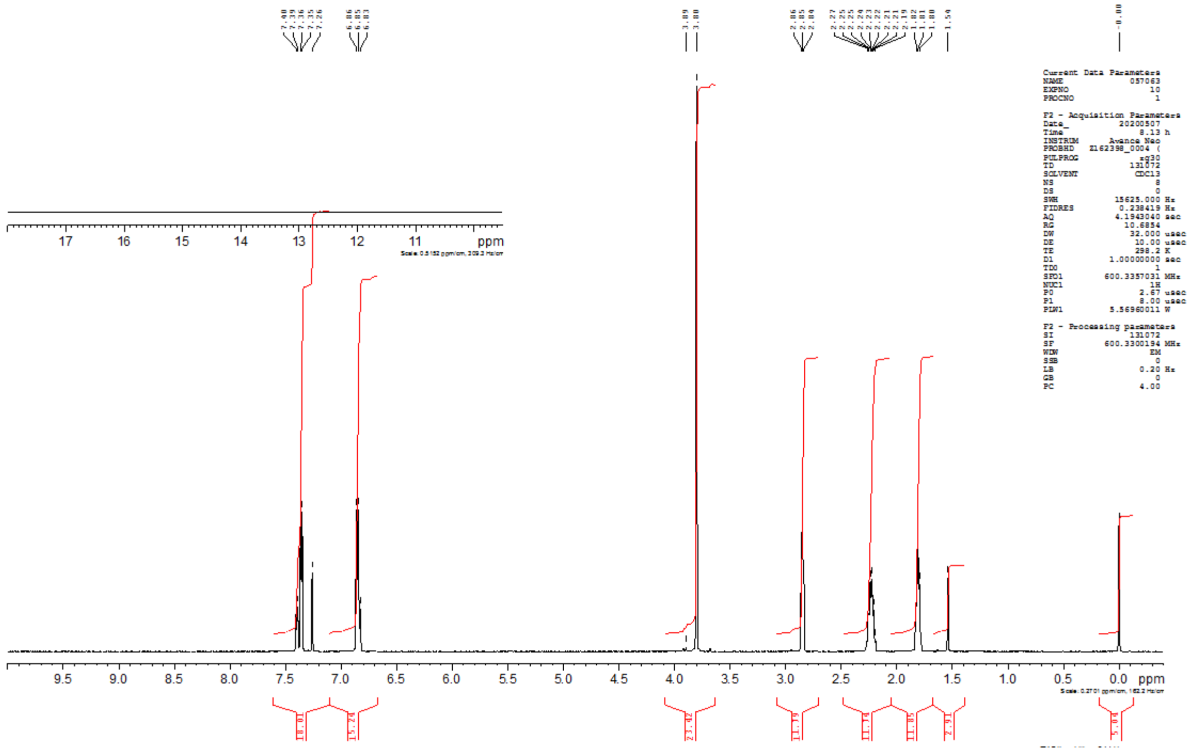

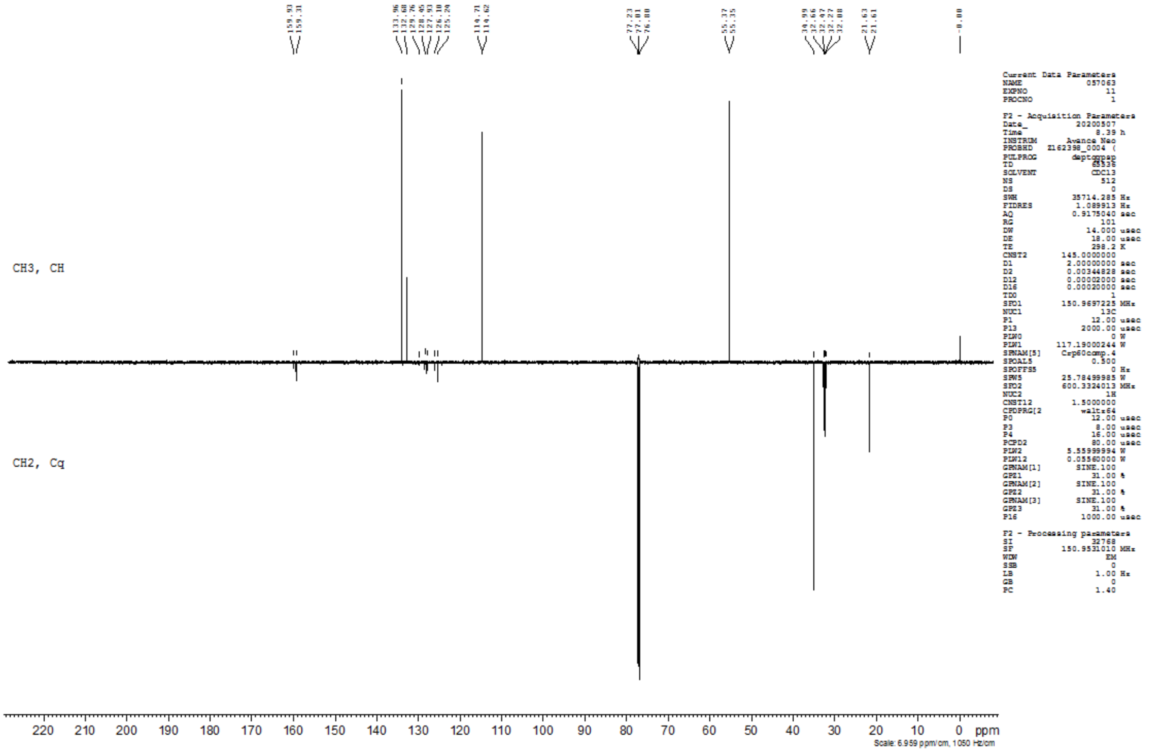

1-Bromo-4-(4,4,4-trifluorobutoxy)benzene (S-4)

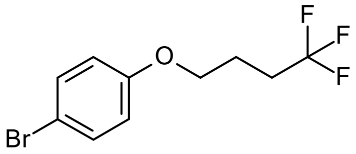

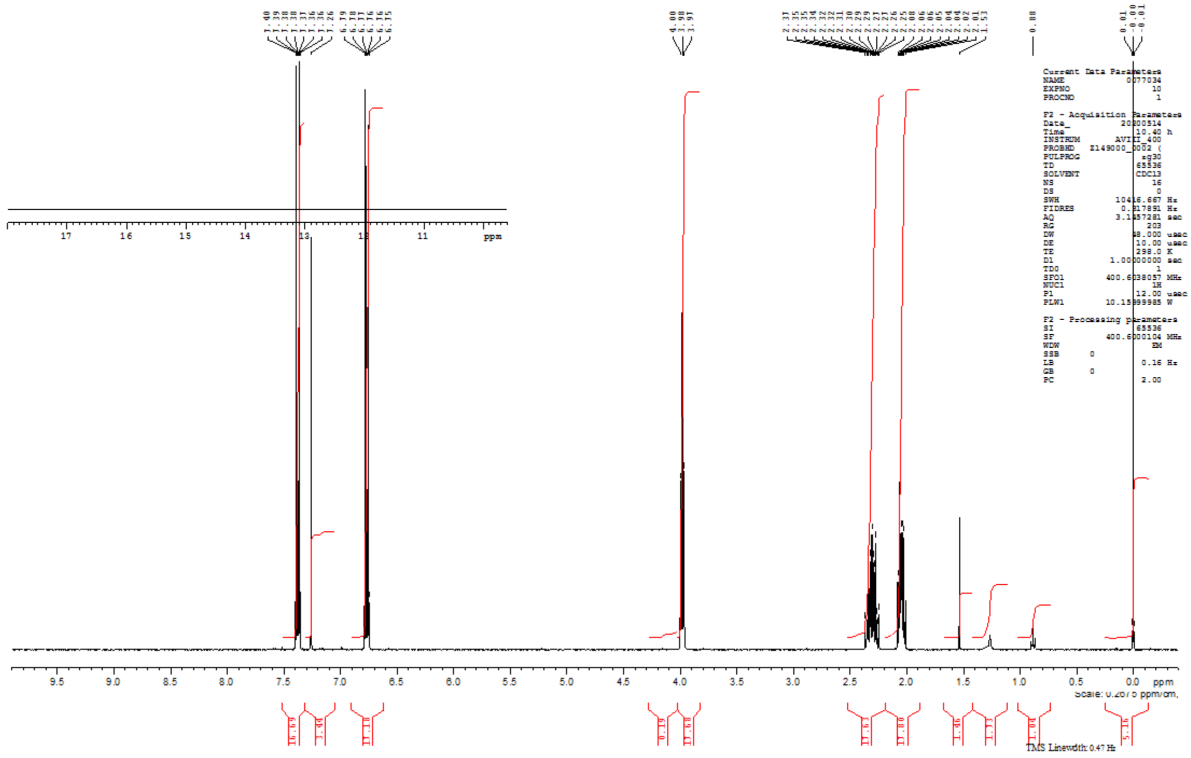

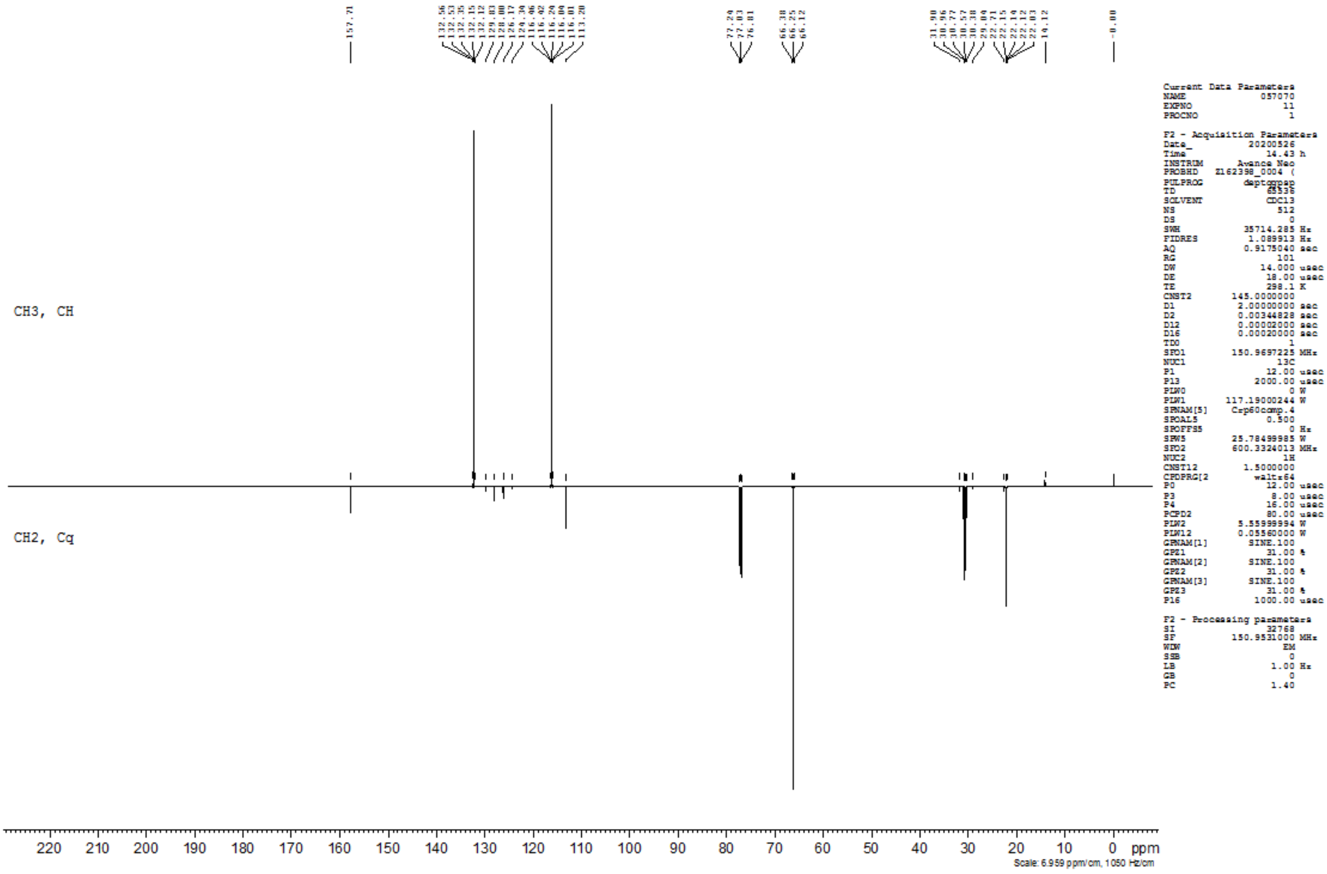

1-Chloro-4-(5,5,5-trifluoropentyl)benzene (16)

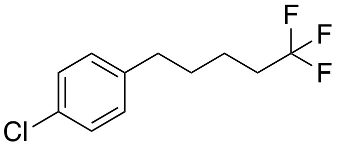

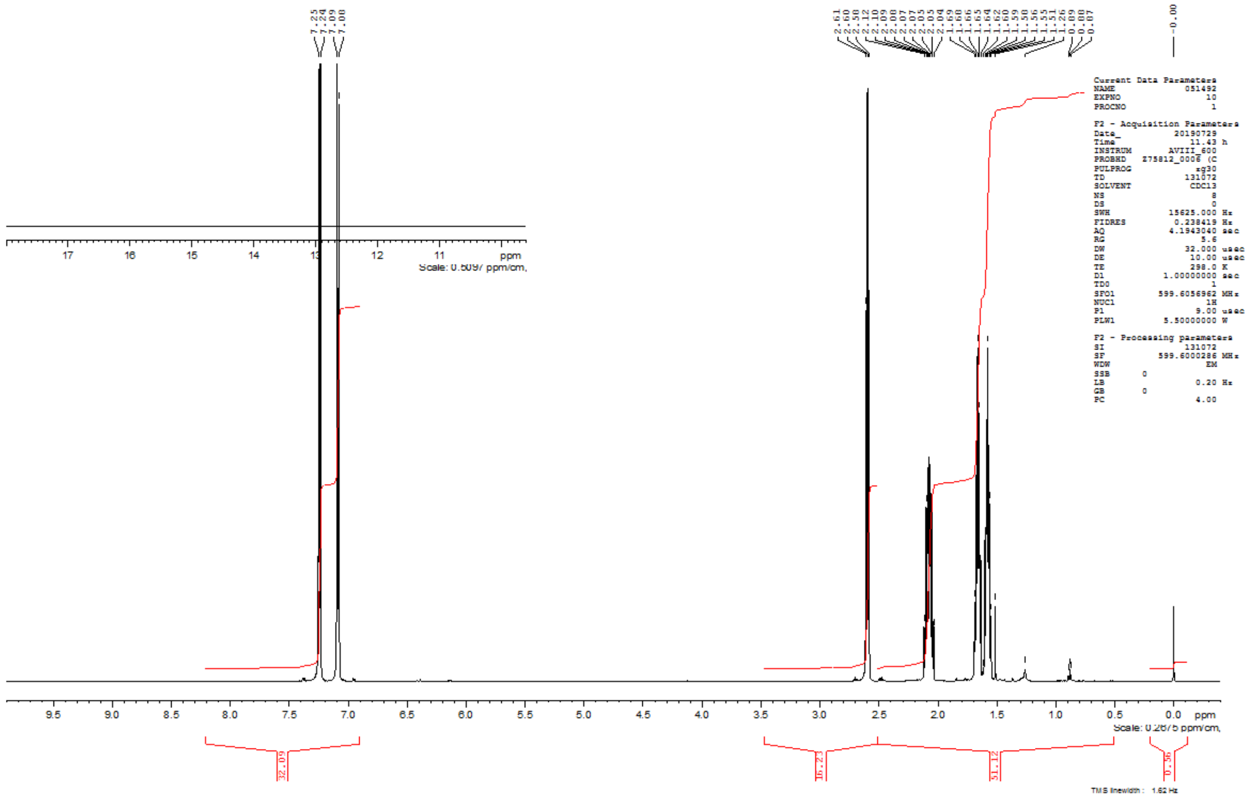

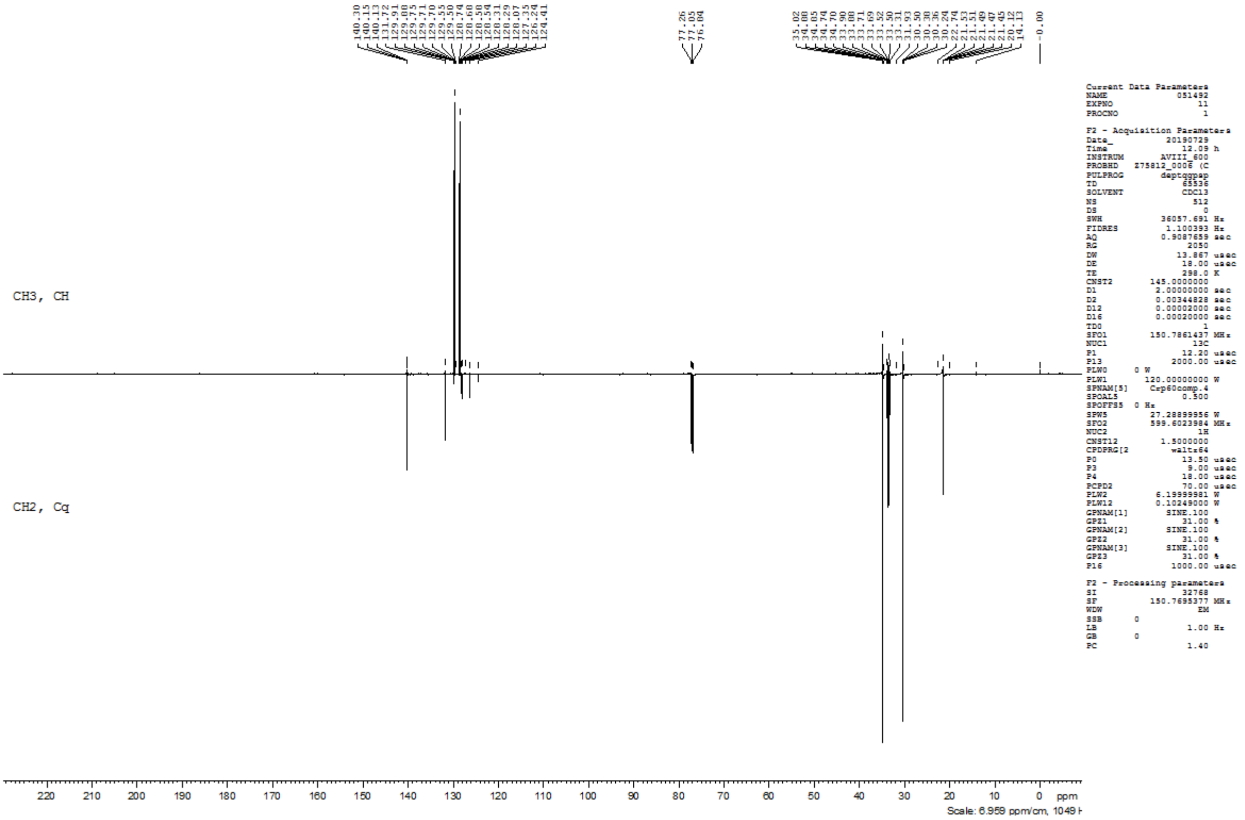

2,5-Dichloro-8,9-dihydro-7*H*-benzo[7]annulene (17)

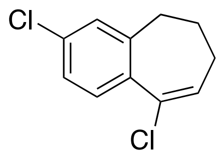

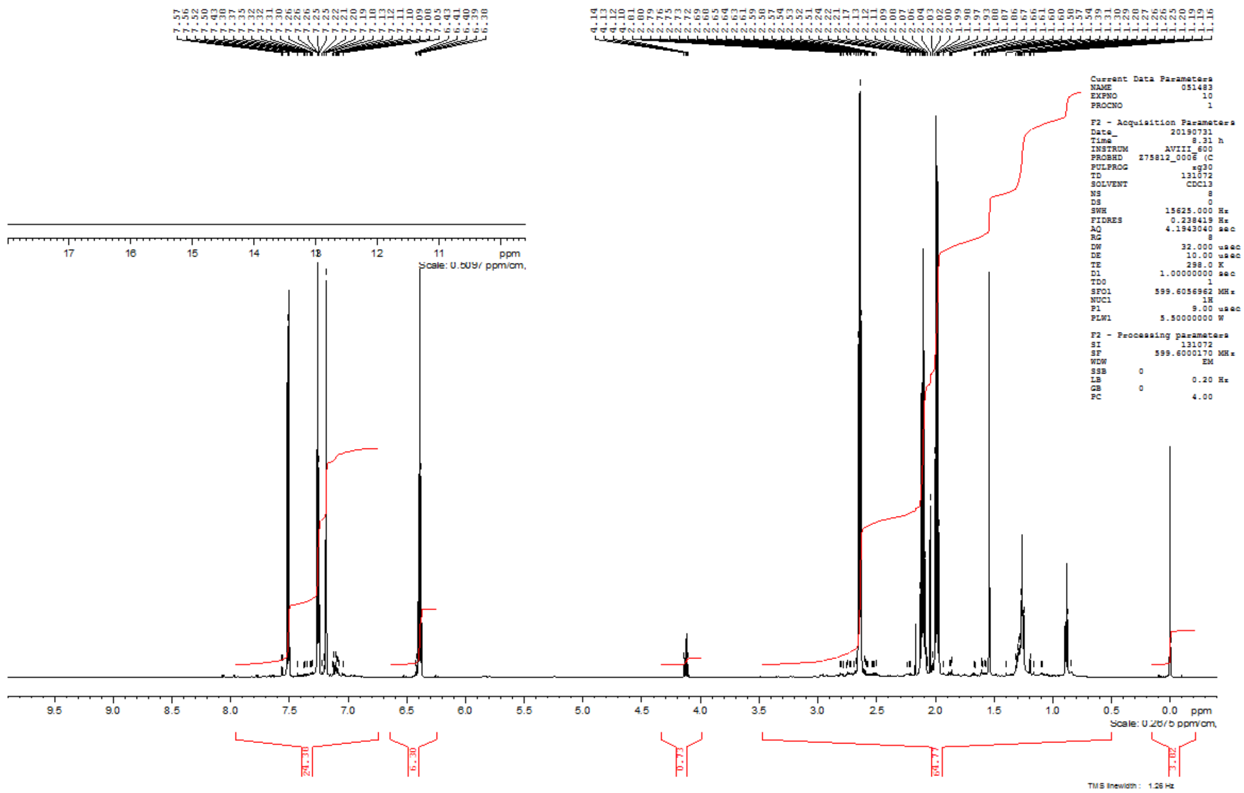

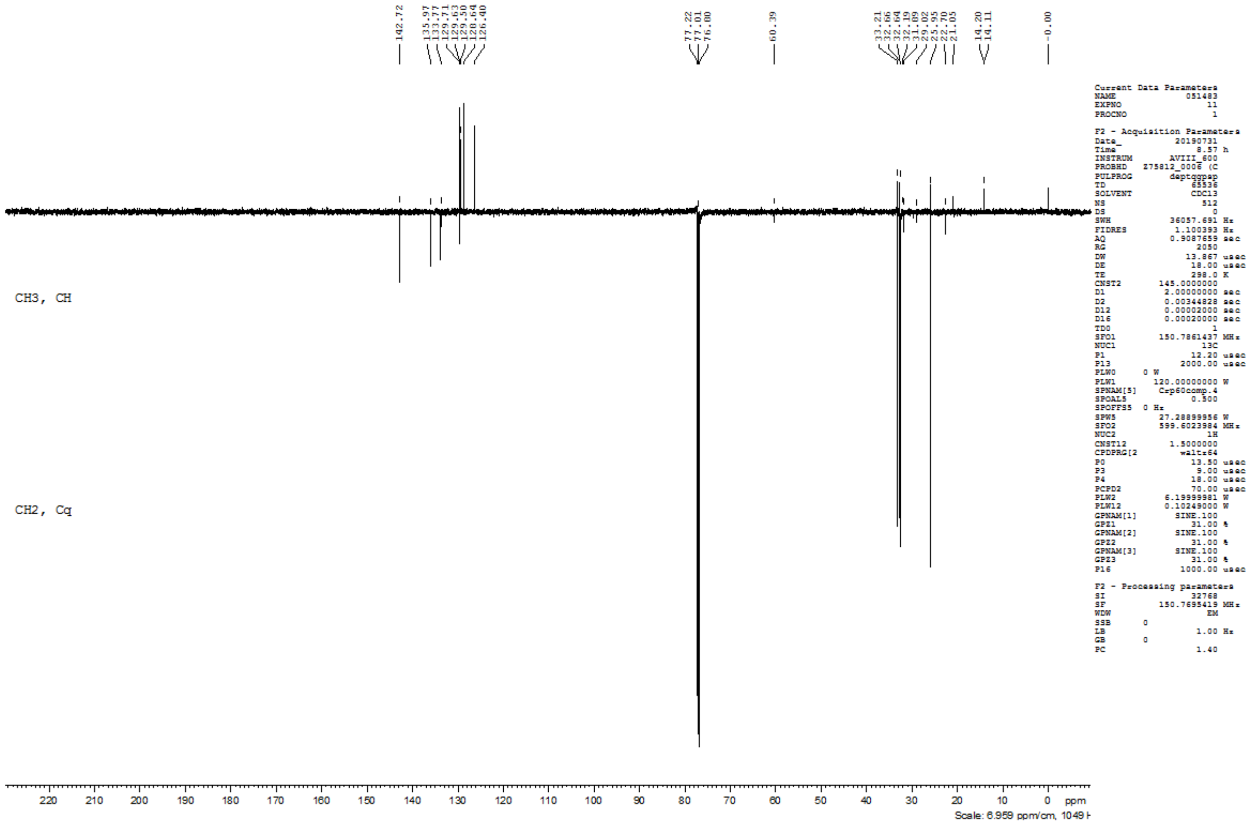

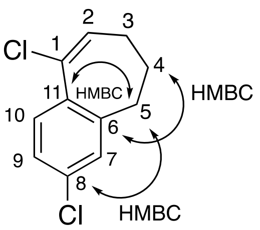

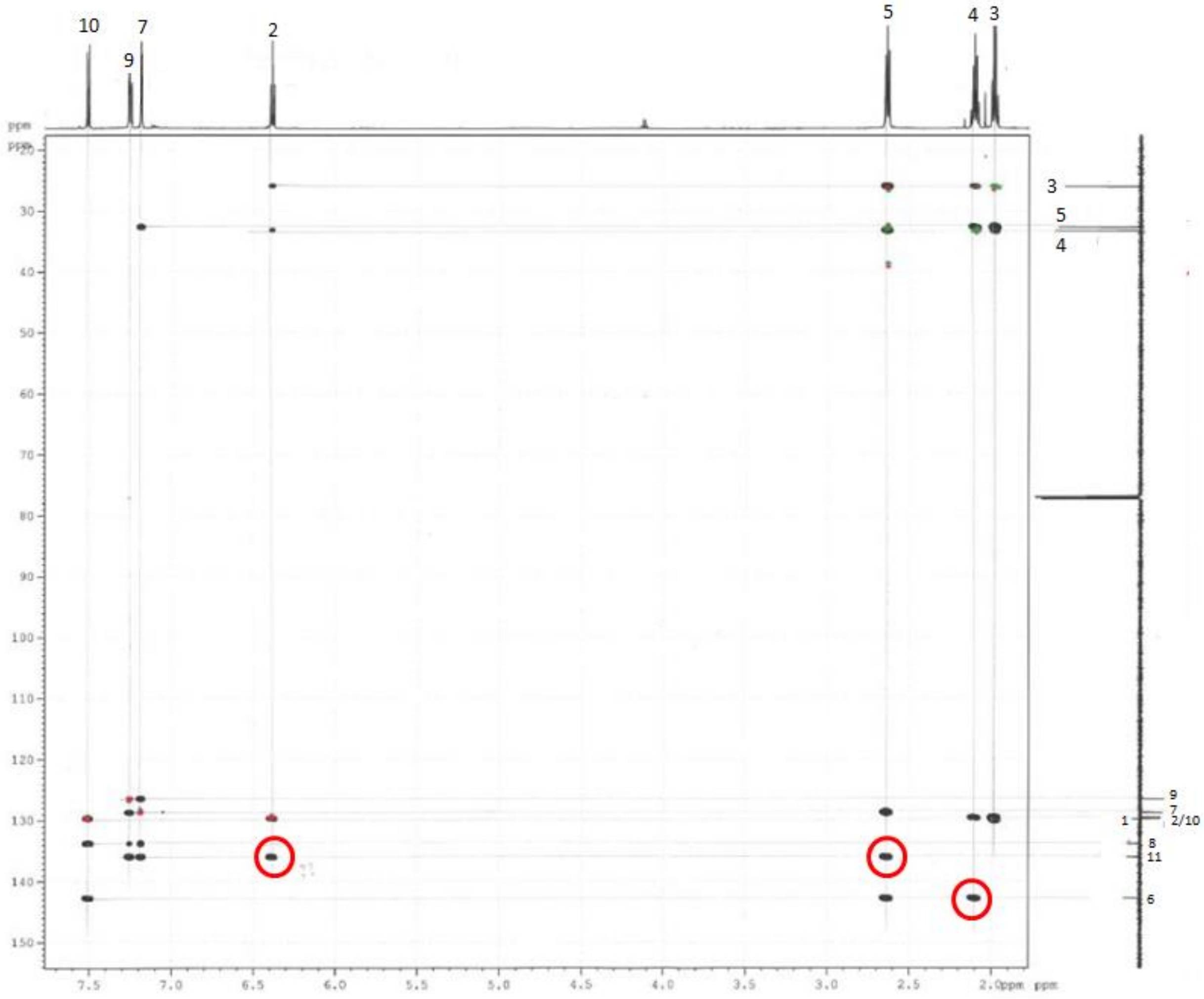

^{1}H-^{13}C-HSQCs (red and green) and ^{1}H-^{13}C-HMBCs (black) of 2,5-dichloro-8,9-dihydro-7*H*-benzo[7]annulene (**17**).

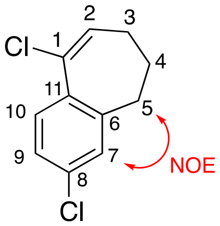

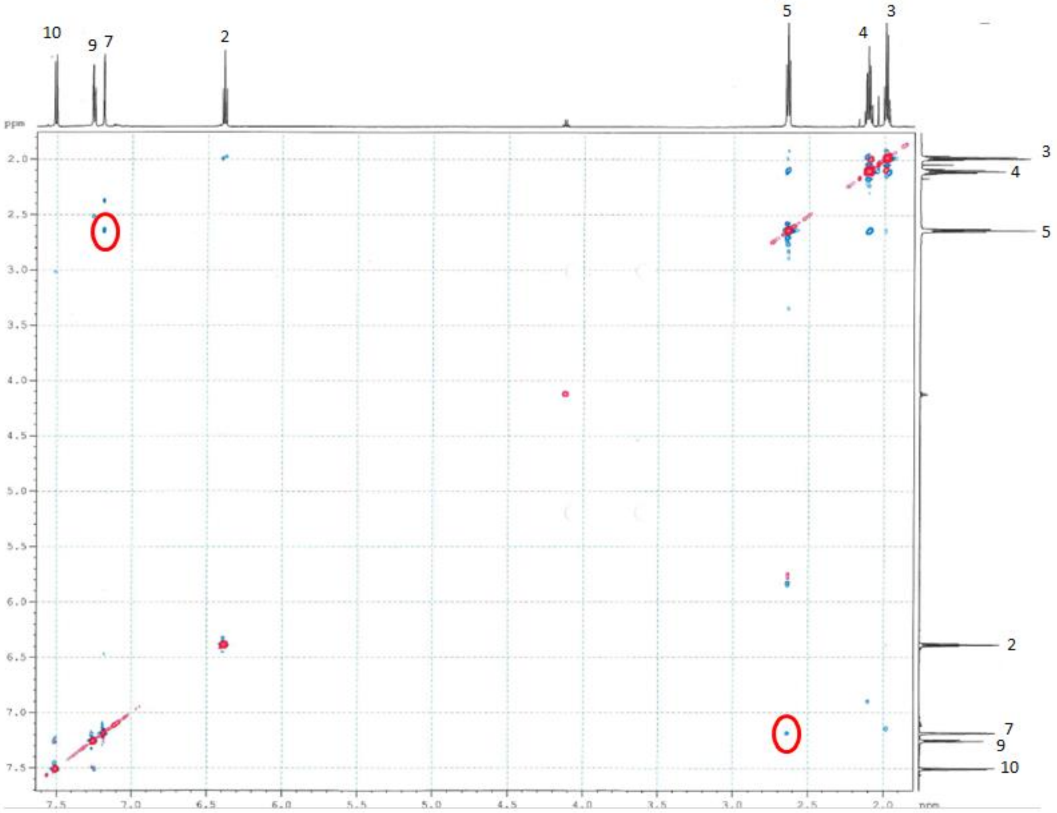

NOESY of 2,5-dichloro-8,9-dihydro-7*H*-benzo[7]annulene (**17**)with cross peaks in red and dia peaks in blue.

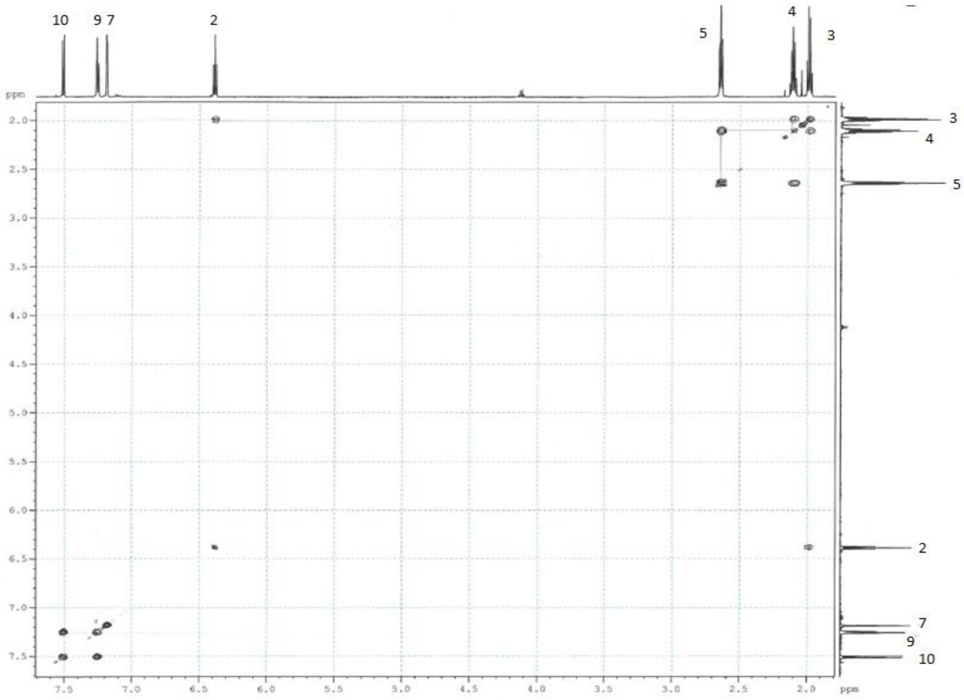

COSY of 2,5-dichloro-8,9-dihydro-7*H*-benzo[7]annulene (**17**).
